# Vitamin D and lumisterol derivatives can act on liver X receptors (LXRs)

**DOI:** 10.1038/s41598-021-87061-w

**Published:** 2021-04-13

**Authors:** Andrzej T. Slominski, Tae-Kang Kim, Shariq Qayyum, Yuwei Song, Zorica Janjetovic, Allen S. W. Oak, Radomir M. Slominski, Chander Raman, Joanna Stefan, Carlos A. Mier-Aguilar, Venkatram Atigadda, David K. Crossman, Andriy Golub, Yaroslav Bilokin, Edith K. Y. Tang, Jake Y. Chen, Robert C. Tuckey, Anton M. Jetten, Yuhua Song

**Affiliations:** 1 Department of Dermatology, University of Alabama at Birmingham, 1670 University Blvd, Rm 476, Birmingham, AL 35249 USA; 2 Comprehensive Cancer Center, Cancer Chemoprevention Program, University of Alabama at Birmingham, Birmingham, AL 35249 USA; 3 Pathology and Laboratory Medicine Service, VA Medical Center, Birmingham, AL 35249 USA; 4 Department of Oncology, Nicolaus Copernicus University Medical College, Romanowskiej str. 2, 85-796 Bydgoszcz, Poland; 5 Department of Chemistry, University of Alabama at Birmingham, Birmingham, AL 35249 USA; 6 Department of Genetics, Genomics Core Facility, University of Alabama at Birmingham, Birmingham, AL 35249 USA; 7 OTAVA LTD, Vaughan, ON L4K 0C3 Canada; 8 School of Molecular Sciences, The University of Western Australia, Perth, WA Australia; 9 Informatics Institute, University of Alabama at Birmingham, Birmingham, AL 35249 USA; 10 Cell Biology Section, National Institute of Environmental Health Sciences, National Institutes of Health, Research Triangle Park, NC 27709 USA; 11 Department of Biomedical Engineering, University of Alabama at Birmingham, Shelby 803, Birmingham, AL 35249 USA

**Keywords:** Biochemistry, Chemical biology

## Abstract

The interactions of derivatives of lumisterol (L3) and vitamin D3 (D3) with liver X receptors (LXRs) were investigated. Molecular docking using crystal structures of the ligand binding domains (LBDs) of LXRα and β revealed high docking scores for L3 and D3 hydroxymetabolites, similar to those of the natural ligands, predicting good binding to the receptor. RNA sequencing of murine dermal fibroblasts stimulated with D3-hydroxyderivatives revealed LXR as the second nuclear receptor pathway for several D3-hydroxyderivatives, including 1,25(OH)_2_D3. This was validated by their induction of genes downstream of LXR. L3 and D3-derivatives activated an LXR-response element (LXRE)-driven reporter in CHO cells and human keratinocytes, and by enhanced expression of LXR target genes. L3 and D3 derivatives showed high affinity binding to the LBD of the LXRα and β in LanthaScreen TR-FRET LXRα and β coactivator assays. The majority of metabolites functioned as LXRα/β agonists; however, 1,20,25(OH)_3_D3, 1,25(OH)_2_D3, 1,20(OH)_2_D3 and 25(OH)D3 acted as inverse agonists of LXRα, but as agonists of LXRβ. Molecular dynamics simulations for the selected compounds, including 1,25(OH)_2_D3, 1,20(OH)_2_D3, 25(OH)D3, 20(OH)D3, 20(OH)L3 and 20,22(OH)_2_L3, showed different but overlapping interactions with LXRs. Identification of D3 and L3 derivatives as ligands for LXRs suggests a new mechanism of action for these compounds.

## Introduction

Vitamin D3 is a product of ultraviolet B (UVB)-induced photochemical transformation of 7-dehydrocholestrol (7DHC), of which the intermediate, pre-vitamin D3, can also isomerize to lumisterol and tachysterol^1–4^. It has pleotropic activities that, in addition to regulation of body calcium homeostasis and musculoskeletal system, include a vast spectrum of actions including stimulation of differentiation and inhibition of proliferation of cells of different lineages, anti-cancerogenic effects and stimulation of innate and inhibition of adaptive immunity and inflammation, and photoprotection^2,5–8^. It also regulates endocrine and central nervous systems and plays an important role in development, and regulates various skin functions in a heterogeneous manner^2,3,9,10^. It was believed that these various, sometime contradictory, activities were mainly mediated by 1,25(OH)_2_D3/2, a product of the sequential hydroxylation of vitamin D3, through interaction with the nuclear vitamin D receptor (VDR)^7,11–14^. However, the interaction of other non-vitamin D low affinity ligands such as lithocholic acid, docosahexaenoic acid, and curcumin with the VDR have been reported^13,15^. It was also assumed that lumisterol neither affects calcium metabolism nor has any significant biological activity, except that UVB-led phototransformation of pre-D3 into lumisterol explained the lack of systemic intoxication by vitamin D3^2^.

We have challenged these dogmas by questioning whether these diverse and sometime opposite effects are regulated by only one receptor (VDR) and one molecule (1,25(OH)_2_D3). This challenge is based on the discovery of an alternative pathway of D3 activation by CYP11A1 with involvement of other CYPs producing at least 15 hydroxyderivatives ((OH)_n_D3) with 20(OH)D3 being the main product of the pathway, which is present in human serum and the epidermis, and in adrenals^16,17^. CYP11A1 also produces 7-dehydropregnenolone (7DHP), which can be modified by steroidogenic enzymes generating Δ7-steroids that upon UVB action, phototransform to novel secosteroids^16–19^. Lastly, CYP11A1 and CYP27A1 act on lumisterol leading to production of at least 9 biologically active derivatives^20–22^. Thus, new pathways generating a large number of biologically active secosteroids and lumisterol-derivatives have now been discovered.

The phenotypic effects of these new hydroxyvitamin D and hydroxylumisterol compounds are elicited by their interactions with specific nuclear receptors (NRs)^23,24^. Recent evidence from our laboratory has shown that CYP11A1-derived (OH)_n_D3, in addition of acting as biased agonists on VDR^25–27^, can, together with lumisterol hydroxyderivatives, act as inverse agonists of retinoic acid-related orphan receptors (ROR) α and γ^26,28^ and as agonists on the arylhydrocarbon receptor (AhR)^29^. This breaks the dogma that VDR serves as the only NR for active forms of vitamin D3.

Liver X receptors (LXR) α and β are NRs for oxysterols (oxygenated derivatives of cholesterol) which after binding of the ligand heterodimerize with the retinoid X receptor (RXR), translocate to the nucleus and activate transcriptional activity of genes containing LXR response elements (LXREs)^30–35^. While being widely distributed in the body and regulating various physiological functions, these receptors show marked tissue selectivity in their expression levels^32,36–39^. Functional LXRs are expressed in the skin^40–43^, the site of lumisterol and vitamin D3 production and also a site of their metabolism^5,10,24^.

Guided by the information above, the similarity of chemical structures of lumisterols and 7DHC (pro-vitamin D3) to cholesterol/oxysterols and retrospective analysis of microarray data deposited at the NCBI GEO (GSE117351)^29,44^ identifying LXR/RXR as a potential NR for 1,25(OH)_2_D3 and 20,23(OH)_2_D3, we have performed extensive experimental and in silico analyses to define LXRα and β as the nuclear receptors for vitamin D3 and lumisterol derivatives.

## Results and discussion

### RNA sequencing (RNA-Seq), bioinformatics and molecular analyses

**Figure 1:**
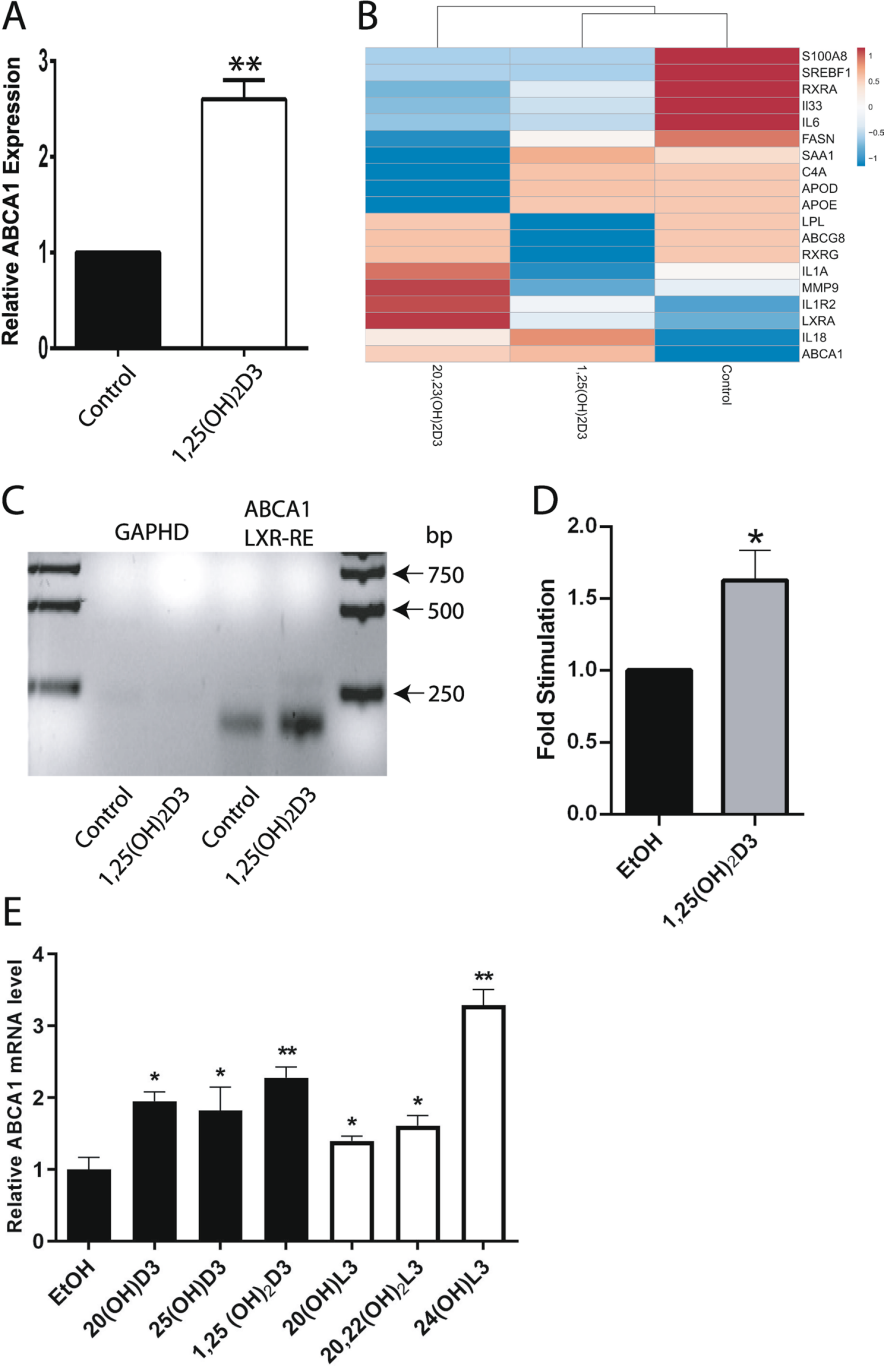
1,25(OH)_2_D3 and CYP11A1-derived secosteroids stimulate LXR activated *ABCA1* gene expression in HaCaT keratinocytes. Relative expression of *ABCA1* gene after treatment with 10^–7^ M 1,25(OH)_2_D3 and 20,23(OH)_2_D3 for 6 (**A**) and 24 h (**B**). Values were retrieved from microarray data deposited at the NCBI GEO (GSE117351)^44^. ChIP analysis performed on isolated nuclei from HaCaT keratinocytes treated with 1,25(OH)_2_D3 using antibody against both LXRα and β, showed significant stimulation of the LXRα/β binding to the LXR-RE of the *ABCA1* (**C**) but not the control gene, *GADPH*. Means ± SD from 3 independent experiments (**D**). QPCR quantification of *ABCA1* in RNA from HaCaT keratinocytes (n = 3) treated with 20(OH)D3, 25(OH)D3, 1,25(OH)_2_D3, 20(OH)L3, 20,22(OH)_2_L3 and 24(OH)L3 (E). *p < 0.5 and **p < 0.01 by student t-test. The experiment was repeated 3 times. The heatmap was prepared using ClustVis software (https://biit.cs.ut.ee/clustvis/).

Retrospective analysis of the microarray data deposited at the NCBI GEO (GSE117351)^44^, identified LXR/RXR as the second nuclear receptor complexes after VDR/RXR, based on ranking in the canonical (p = 0.0039) and toxicity-related (p = 0.0041) pathways activated in primary human keratinocytes treated with 1,25(OH)_2_D3 for 24 h. For treatment with 20,23(OH)_2_D3, LXR/RXR was identified as the fourth nuclear receptor complex base on ranking in the canonical and toxicity-related pathways, p = 0.0174 and p = 0.02, respectively. LXR/RXR was also implicated in the activation of these pathways (p = 0.0085 p = 0.0089, respectively) in cells treated with 1,25(OH)_2_D3 for 6 h. Analysis of the relative expression of the known LXR target gene, *ABCA1,* showed that 1,25(OH)_2_D3 and 20,23(OH)_2_D3 stimulated *ABCA1* expression 2.4- and 2.3-fold, respectively, in immortalized human epidermal keratinocytes (HaCaT cells) after 24 h of treatment and 2.4 and 2.8 times after 6 h treatment with 1,25(OH)_2_D3 (Fig. 1A,B). The regulation of LXR targets by these secosteroids was supported by chromatin immunoprecipitation (ChIP) analysis with chromatin isolated from HaCaT keratinocytes treated with 1,25(OH)_2_D3 and antibodies against LXRα and β, which showed significant stimulation of LXRα/β binding to the LXRE within the *ABCA1* promoter region (Fig. 1C,D). In addition, qPCR analysis confirmed the stimulation of *ABCA1* RNA expression in HaCaT keratinocytes treated with 20(OH)D3 (precursor of 20,23(OH)_2_D3) and 1,25(OH)_2_D3 and its precursor 25(OH)D3. Increased expression was also observed with CYP11A1-derived hydroxylumisterols (Fig. 1E).

**Figure 2:**
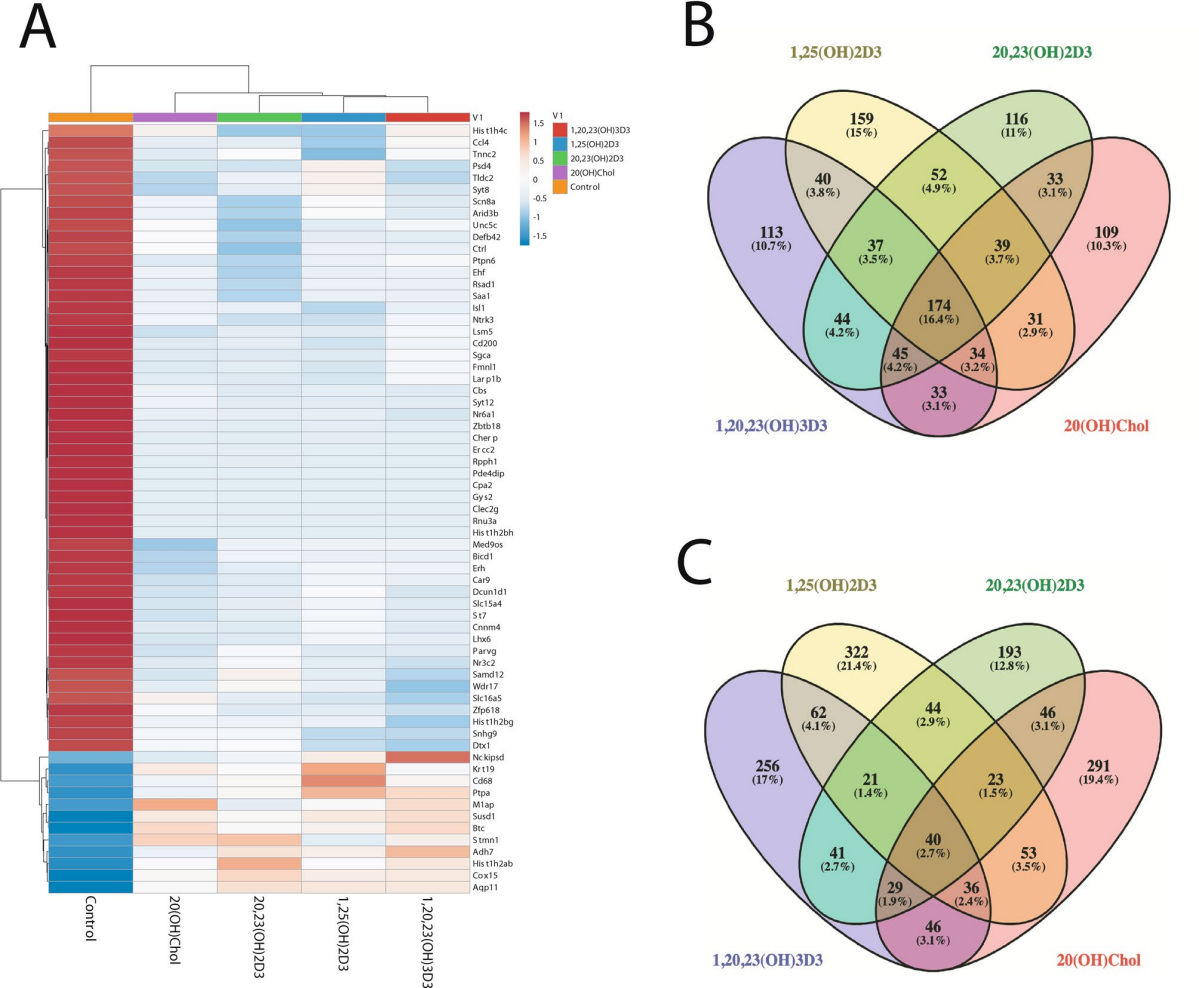
RNAseq analysis of changes in gene expression in murine dermal fibroblasts treated with 10^–7^ M 1,25(OH)_2_D3, 20,23(OH)_2_D3, 1,20,23(OH)_3_D3 or 20(OH)cholesterol (20(OH)C, a native LXR ligand) for 24 h. Heat map of the gene expression pattern (**A**) with corresponding Venn diagrams shown for down (**B**) or upnregulated (**C**) protein coding genes for absolute fold change ≥ 2 cutoff. The Venn diagrams were prepared using Venny version 2.1.0: https://bioinfogp.cnb.csic.es/tools/venny/index.html. The heatmap was prepared using ClustVis: https://biit.cs.ut.ee/clustvis/.

RNA-Seq analysis was performed on RNA from newborn murine dermal fibroblasts cultured for 24 h in the presence or absence of 10^−7^ M 1,25(OH)_2_D3, 20,23(OH)_2_D3, 1,20,23(OH)_3_D3 or a natural ligand for LXR, 20-hydroxycholesterol (20(OH)C), which served as the positive control. The raw data are deposited at the NCBI GEO (GSE145818). The relative gene expression patterns for one time point (24 h) and one fixed concentration of the ligand (10^−7^ M) were normalized vs vehicle (negative control) and their hierarchical clustering revealed distinct, opposite or overlapping patterns (Fig. 2). The common up- and downregulated protein coding genes using FC ≥ 2 are shown in the heat map (Fig. 2A) and Supplementary Table 1. A list of all genes with their raw values is accessible at the NCBI GEO (GSE145818). The Venn diagrams (Fig. 2B,C) show that the number of genes upregulated by either 20,23(OH)_2_D3, 1,20,23(OH)_3_D3, 1,25(OH)_2_D3 or 20(OH)C, using FC ≥ 2, was 437, 531, 601 and 564, respectively, while the number of genes downregulated was 540, 520, 566 and 498, respectively. The number of common genes either up- or downregulated by all compounds was 40 (2.7%) and 174 (16.4%), respectively, with the corresponding number of genes affected by only the D3 derivatives tested being 61 (4.1%) and 211 (19.9%). The number of genes solely upregulated and downregulated by 20,23(OH)_2_D3, 1,20,23(OH)_3_D3, 1,25(OH)_2_D3 or 20(OH)C were 193 (12.8%), 256 (17%), 322 (21.4%) or 291 (19.4%) and 116 (11%), 113 (10.7%), 159 (15%) or 109 (10.3%), respectively. The number of genes up and down-regulated by structurally related 20,23(OH)_2_D3, 1,20,23(OH)_3_D3, and 1,20,23(OH)_3_D3, 1,25(OH)_2_D3, were 131 (8.7%), 300 (28.3%) and 159 (10.6%), 285 (26.9%), respectively. The number of genes up- and down-regulated by 20,23(OH)_2_D3 and 1,25(OH)_2_D3 were 128 (8.5%) and 302 (28.5%), respectively. The number of genes up- and down-regulated for structurally distant molecules 20,23(OH)_2_D3 vs 20(OH)C, 1,20,23(OH)_3_D3 vs 20(OH)C and 1,25(OH)_2_D3 vs 20(OH)C were 138 (9.2%) and 291 (27.4%), 151 (10.1%) and 286 (26.9%), and 152 (10.1%), and 278 (26.2%), respectively. The hypergeometric test for all pairwise upregulated genes in the intersection was significant (p < 0.05). For down-regulated genes among conditions B (20,23(OH)_2_D3), C (1,20,23(OH)_3_D3), and D (20(OH)C), the non-intersection regions of the three pairwise comparisons (B+C−/B−C+, B+D−/B−D+, and C+D−/C−D+) were found to be significant.

Ingenuity Pathway Analysis (IPA) using an absolute fold change ≥ 2 cutoff showed that after VDR/RXR, LXR/RXR was the next top ranked nuclear receptor pathway activated by 1,25(OH)_2_D3 in its regulation of canonical (p = 0.02) and toxicity-related (p = 0.022) responses (Supplementary Tables 2, 3). With respect to 20,23(OH)_2_D3 and 1,20,23(OH)_3_D3, respectively, LXR/RXR was ranked as the top nuclear receptor pathway implicated in the induction of canonical (p = 0.013 and 0.016) and toxicity-related (p = 0.014 and 0.048) responses (Supplementary Tables 4–7). Interestingly, estrogen-mediated S-phase entry was identified for 20,23(OH)_2_D3 as the top ingenuity signaling pathway (Supplementary Table 4). Moreover, LXR/RXR was not identified as a significant target for 20(OH)C used at concentration 10^–7^ M in the canonical pathway (p = 0.30) and represented the third receptor complex in the toxicity pathway (p = 0.31) after RAR and VDR (Supplementary Tables 8, 9).

In addition, Gene Ontology Enrichment Analysis (GO, Panther Platform) has indicated relations between LXR and VDR signaling regulated by 20,23(OH)_2_D3 (one of the most active and promising, non-toxic vitamin D3- hydroxyderivative) in mice fibroblasts (Supplementary Table 10). Similarly to IPA analysis, GO indicated that 20,23(OH)_2_D3 is involved in enrichment of vitamin D response element binding- and vitamin D receptor binding-gene sets, as well as influencing the expression of genes involved in the regulation of metabolic transformations of vitamin D compounds. The main gene sets overrepresented by 20,23(OH)_2_D3 and involved in regulation of the LXR/RXR pathway included low-density lipoprotein particle receptor activity, lipoprotein lipase activity, fatty acid synthase activity, retinoid X receptor binding, S100 protein binding, ABC-type transmembrane transporter activity, retinoic acid-responsive element binding, low-density lipoprotein particle binding, high-density lipoprotein particle receptor activity, high-density lipoprotein particle binding, oxidized low-density lipoprotein particle receptor activity, oxysterol binding, cholesterol transfer activity, apolipoprotein A-I binding, peroxisome proliferator activated receptor binding, interleukin-1 type II receptor antagonist activity, hydroxymethylglutaryl-CoA synthase activity, hydroxymethylglutaryl-CoA reductase (NADPH) activity, fatty-acyl-CoA binding, apolipoprotein receptor activity, very-low-density lipoprotein particle receptor activity, farnesyl-diphosphate farnesyltransferase activity, chylomicron binding, bile acid receptor activity, nitric-oxide synthase regulator activity, cholesterol binding, sterol response element binding, nitric-oxide synthase binding and acetyl-CoA binding (Supplementary Table 10).

**Figure 3:**
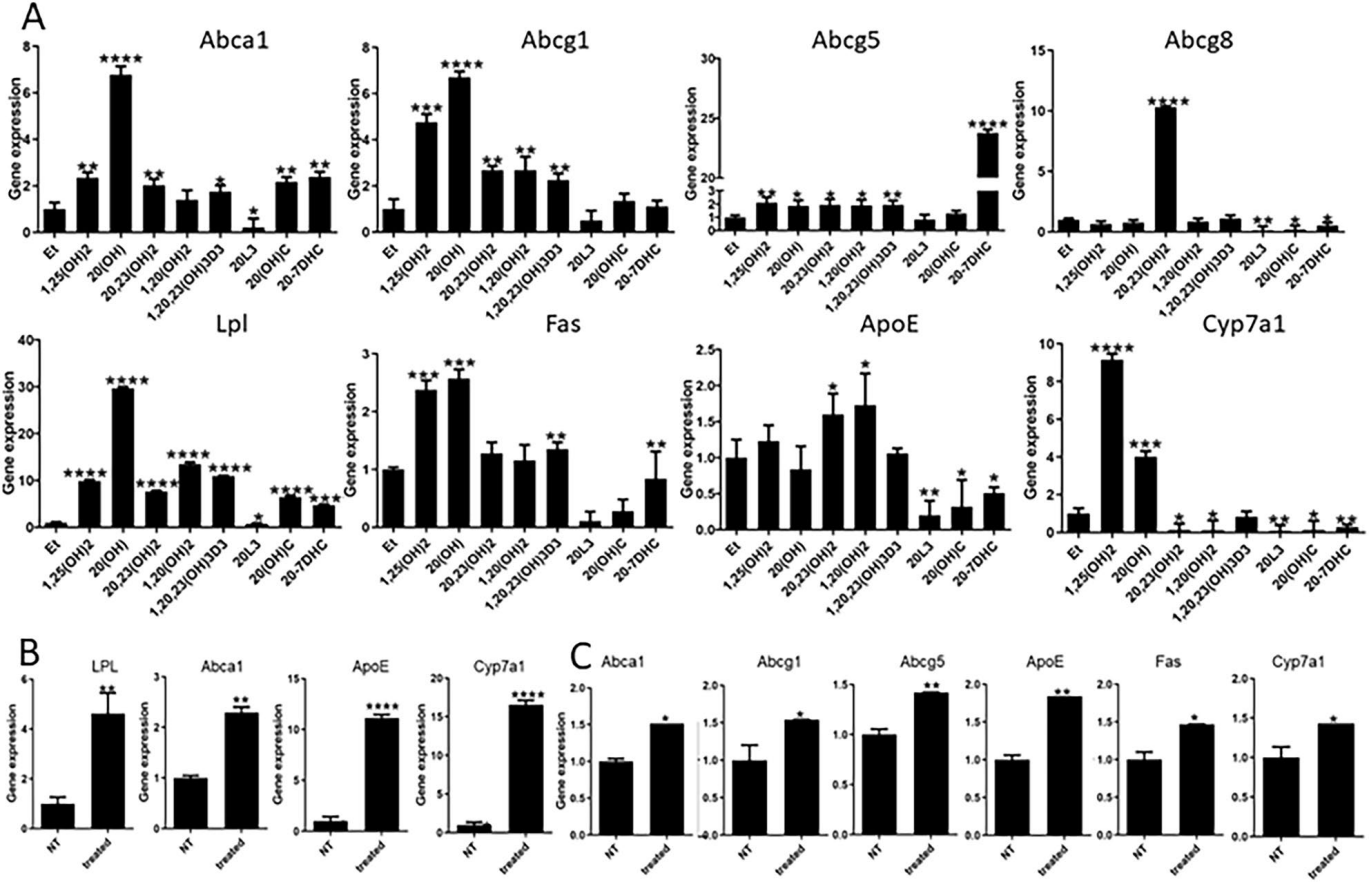
Vitamin D and lumisterol hydroxyderivatives stimulate the expression of LXR-dependent genes. (**A**) QPCR analysis of changes in expression of genes downstream of LXR in murine dermal fibroblasts treated with 10^–7^ M 1,25(OH)_2_D3, 20(OH)D3, 1,20(OH)_2_D3, 20,23(OH)_2_D3, 1,20,23(OH)_3_D3, 20(OH)7DHC, 20(OH)L3 or 20(OH)C, or ethanol (control) for 24 h. (**B**) Stimulation of LXR-dependent gene expression in the brain of SKH-1 (**B**) or Ptch^**+/−**^/SKH-1 (**C**) mice (n = 3 per group) treated with 20 µg/kg of 20(OH)D3, respectively, for 6 h. Data are presented as means ± SD, n = 3. Statistical analysis was done using the t-test: *p < 0.05, **p < 0.01, ***p < 0.001 or ****p < 0.0001 versus control (ethanol).

Since RNA-Seq analysis identified LXR as a possible target for D3-hydroxyderivatives, we compared the effects of several D3-hydroxyderivatives (1,25(OH)_2_D3, 20(OH)D3, 20,23(OH)_2_D3, 1,20(OH)_2_D3, 1,20,23(OH)_3_D3) and a representative hydroxylumisterol (20(OH)L3) on the expression of genes downstream of LXR^32,36^ with those of 20(OH)C and its precursor 20(OH)7DHC, both natural ligands of LXR that serve as positive controls (Fig. 3A). These analyses showed that all compounds, except for 1,20(OH)_2_D3, stimulated *Abca1* expression in murine dermal fibroblasts, whereas 20(OH)L3 inhibited its expression. All D3-hydroxyderivatives, but not 20(OH)C or 20(OH)L3, enhanced the expression of *Abcg1* and *Abcg5*, while 20(OH)7DHC stimulated only *Abcg5*. The expression of *Abcg8* was induced only by 20,23(OH)_2_D3 and was inhibited by 20(OH)C, 20(OH)7DHC and 20(OH)L3. Most of the compounds stimulated *Lpl* (lipoprotein lipase) expression except for 20(OH)L3, which inhibited it. 1,25(OH)_2_D3, 20(OH)D3, 1,20,23(OH)_3_D3 and 20(OH)7DHC stimulated the expression of *Fas* (fatty acid synthase). 1,25(OH)_2_D3 and 20(OH)D3 stimulated *Cyp7a1* (cytochrome P450 isoform 7A1) expression, but was inhibited by 1,20(OH)_2_D3, 1,20,23(OH)_3_D3, 20(OH)C, 20(OH)7DHC and 20(OH)L3. *ApoE* (apolipoprotein E) expression was enhanced by 20,23(OH)_2_D3 and 1,20(OH)_2_D3, but inhibited by 20(OH)C, 20(OH)7DHC, and 20(OH)L3. Importantly, subcutaneous injection of 20(OH)D3 (20 µg/kg) stimulated expression of *Lpl*, *Abca1*, *ApoE* and *Cyp7a1* in brains of SKH-1-M mice (Fig. 3B), while 20(OH)D3 at 10 µg/kg stimulated expression of *Abca1, Abcg1*, *Abcg5, ApoE, Fas* and *Cyp7a1* in brains of SKH-1-M mice (Fig. 3C).

### Molecular docking studies of a series of ligands potentially targeting LXRα and LXRβ based on LXR crystal structures

7DHC and lumisterol (L3) derivatives have very similar structures to those of oxysterols^10,31^. Tachysterol (T3) and L3 are photoderivatives of pre-D3 formed after extended exposure to UVB^4^. To obtain insights into the nature of ligand–LXR interaction, two LXRα (PDBID 5AVI, 3IPQ) and three LXRβ (PDBID 5HJP, 1PQC, 1UPV) structures with the most diverse conformations were selected for the docking studies (Supplementary Fig. S1) to predict the binding poses of 84 D3 and L3 hydroxyderivatives with LXRα and LXRβ (Supplementary Tables 11–15). Docking scores were determined to evaluate the potential binding of each tested compound with LXRα and LXRβ (Supplementary Table 16).

To test the reliability of our docking studies, we re-docked the co-crystalized ligands with LXRα and LXRβ. All the corresponding ligand pairs (docked VS co-crystalized) displayed root mean square deviation (RMSD) in the range 0.5 Å–0.7 Å (acceptable values are ≤ 2.0 Å), which demonstrated that the docking reproduced the co-crystalized (experimental) ligand poses with high precision (Supplementary Fig. S2).

Overall, all 84 compounds were predicted to bind tightly to the LXRs due to significant hydrophobic interactions and intermolecular hydrogen bond formation. Due to the hydrophobic nature and similar size of the compounds tested, they all fit well into the hydrophobic cavity of the binding site (Supplementary Figs. S3 and S4). In addition to the strong hydrophobic interactions in the central region of the ligand binding domain (LBD), two hydrogen bonding regions on both ends of the LBP contribute to the binding stability of the ligand–LXR complex. Detailed information about the hydrogen bonds and hydrophobic interaction for all 84 ligands is shown in Supplementary Tables 11–15. 3-D representations for the example ligands occupying the hydrophobic cavity of the LXRα LBD (PDBID 5AVI, 3IPQ) and LXRβ LBD (PDBID 5HJP, 1UPV) are shown in Supplementary Figs. S5, S6, S7 and S8 of the Supplementary file. Although all these compounds can bind to the LBD of LXRs, different ligands could have different binding affinities with LXRs and induce different conformational and dynamical motion changes of LXRs to have different effects on gene expression. This is supported by detailed structural, conformation and dynamical motion analyses shown in “Materials and methods”. Binding thermodynamics analyses in “Materials and methods” further supports ligand binding specificity with LXRs.

Glide XP docking scores of top ranking poses for the tested ligands for each LXR conformation (Supplementary Table 16) show that novel secosteroidal, 7DHC, L3 and T3 derivatives with a full-length or short side chain have similar docking scores to the natural ligands, 20(OH)C and 22(OH)C (positive controls), for LXRs. Since T3 compounds are unstable^4^, and undergo oxidative modification and 7DHC is reduced to cholesterol by the action of Δ7reductase, we focused our subsequent studies on D3 and L3 hydroxyderivatives.

### Functional studies

#### Activation of transcriptional functions of LXRα and LXRβ

**Figure 4:**
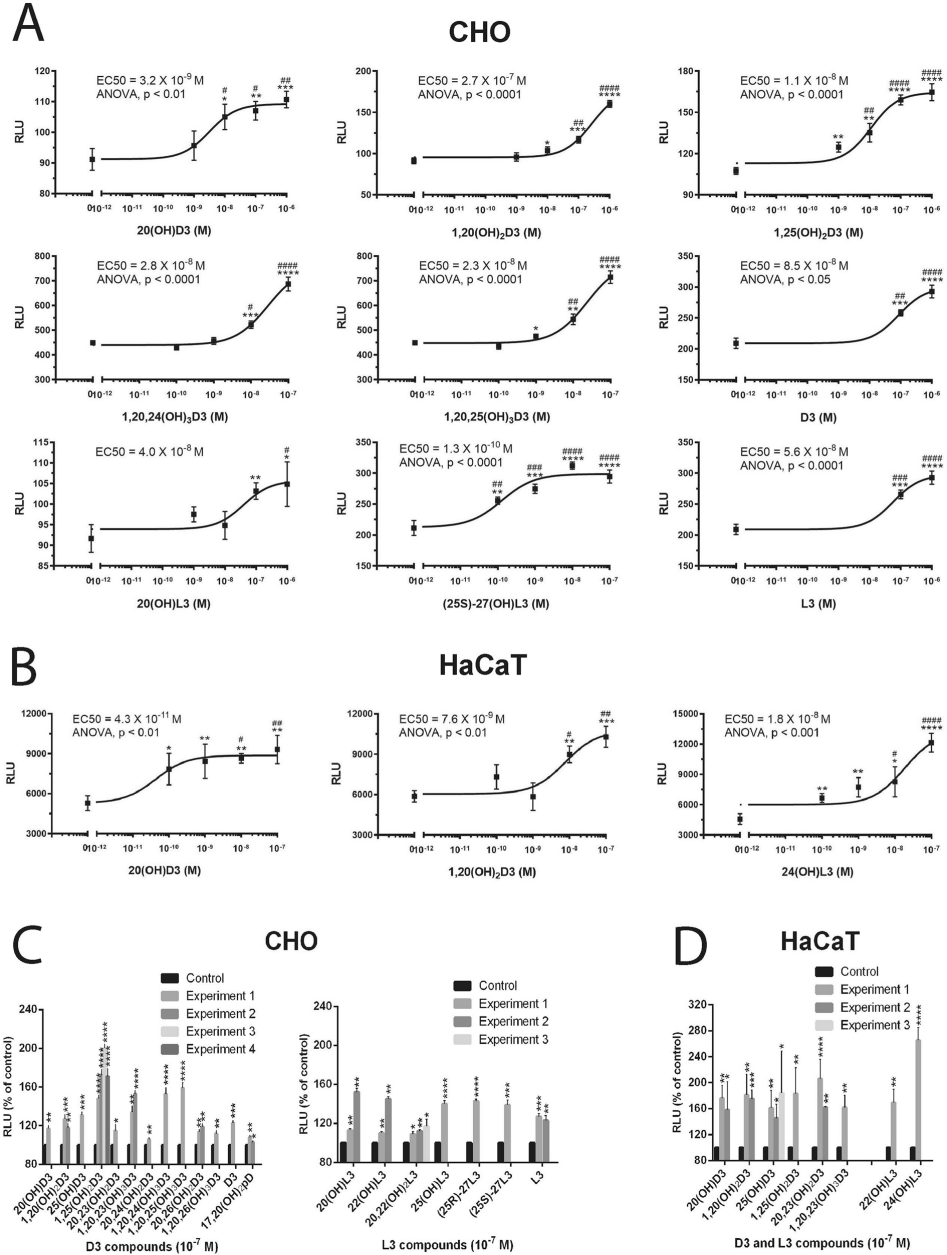
L3 and D3-derivatives activate a LXR-response element (LXRE)-driven reporter in CHO cells (**A**,**C**) and human HaCaT keratinocytes (**B**,**D**). Representative dose response curves are in (**A**) and (**B**), while a summary of assays performed with 10^–7^ M ligands is presented for each experiment separately for CHO cells (**C**) and HaCaT keratinocytes (**D**). Data are presented as means ± SE, (n = number of assays). Analysis was done using one-way ANOVA for dose responses and the t-test: *p < 0.05, **p < 0.01, ***p < 0.001 or ****p < 0.0001 versus control (ethanol).

Using the luciferase reporter gene containing the LXR-response element (LXRE), we measured the induction of transcriptional activity in CHO cells and HaCaT keratinocytes by a series of vitamin D3 and L3 compounds (Fig. 4). Figure 4A,B showed a dose-dependent activation of luciferases activity by hydroxyderivatives of D3 and L3, as well as their precursors, with EC_50_ values ranging from 10^–7^ to 10^–10^ M. Figure 4C showed the stimulation of luciferase activity in CHO and HaCaT cells with L3- and D3-derivatives at a concentration of 10^–7^ M. While all compounds tested stimulated the transcriptional activity, 1,25(OH)_2_D3, 1,20,23(OH)_3_D3, 1,20,24(OH)_3_D3 and 1,20,25(OH)_3_D3 showed the strongest stimulatory effects among the D3 derivatives. 17,20(OH)2pD, which has a 2C-side chain, displayed the weakest stimulant in CHO cells. These results are in agreement with higher docking scores for the former compounds in comparison to the short side chain derivatives (Supplementary Table 16). L3-hydroxyderivatives showed stronger stimulatory activity in HaCaT cells than in CHO cells, which indicated a degree of cell type specificity.

#### Ligand binding in LanthaScreen TR-FRET LXRα and β coactivator assays

**Figure 5:**
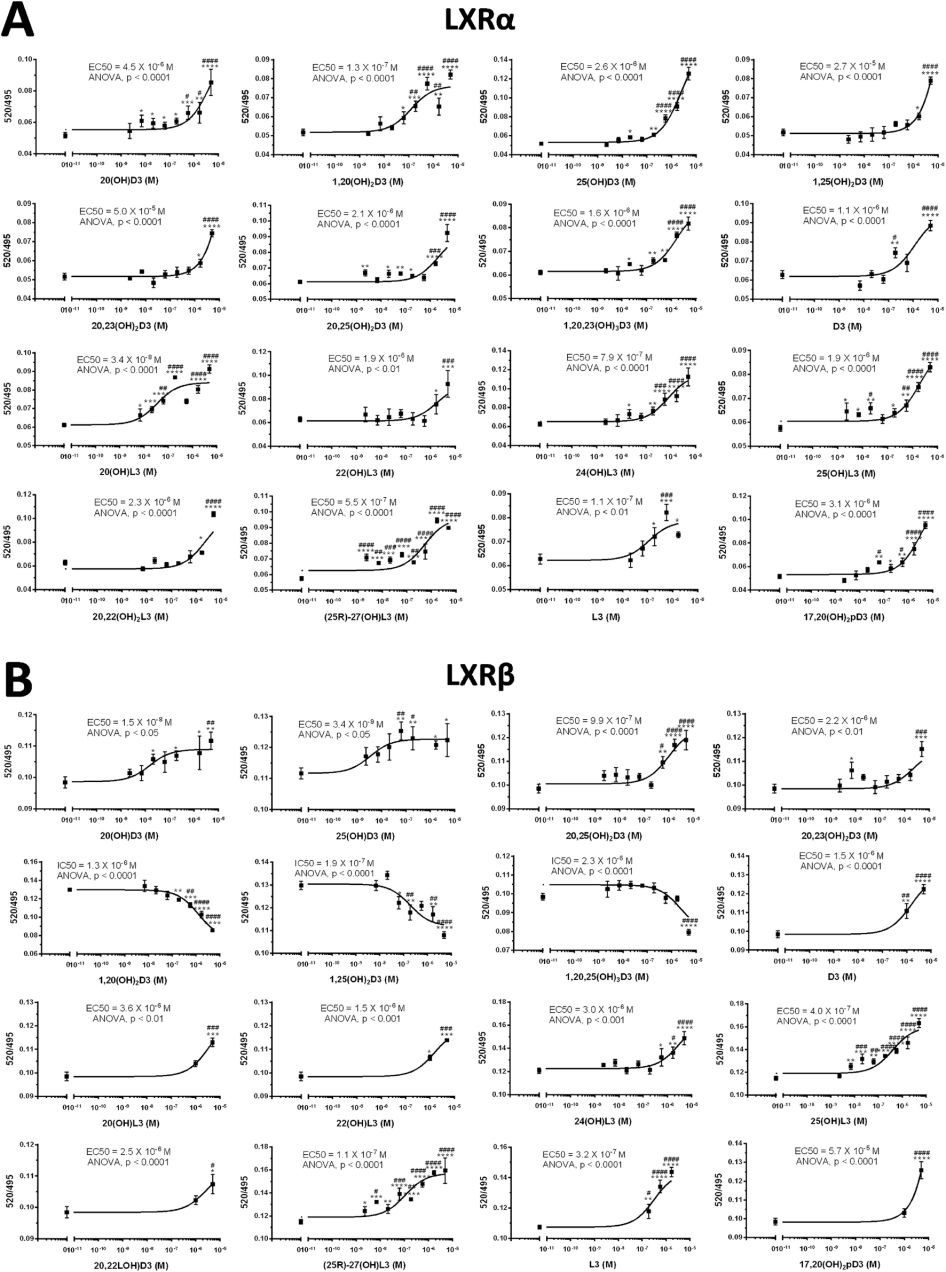
Binding of L3 and D3 derivatives to the LBD of the LXRα (**A**) and β (**B**) in LanthaScreen TR-FRET LXRα and β coactivator assays. (**A**) and (**B**): representative binding curves with values presented as means ± SE, (n = 4). Analysis was done using one-way ANOVA with significance defined as *p < 0.05, **p < 0.01, ***p < 0.001 or ****p < 0.0001.

Figure 5 shows dose dependent binding of L3 and D3 derivatives to the LBD of the LXRα and β. While the majority of metabolites including 20(OH)D3, 25(OH)D3, 20,23(OH)_2_D3, D3, 20(OH)L3, 22(OH)L3, 24(OH)L3, 20,22(OH)_2_L3, 25(OH)L3, (25*R*)27(OH)L3, (25*S*)27(OH)L3 and L3 acted as LXRα agonists with EC_50_ values ranging from 10^–8^ to 10^−6^ M, 17,20(OH)_2_pD displayed an EC50 of only 3 × 10^−5^ M. 1,20,25(OH)_3_D3, 1,25(OH)_2_D3, 1,20(OH)_2_D3 and 25(OH)D3 acted as inverse agonists of LXRα with IC50 values around 10^–6^ M. Notably, all compounds acted as agonists of LXRβ with EC50 values ranging from 10^–9^–10^–5^ M. The average EC_50_ and IC_50_ values are shown in Table 1.

**Table 1:** Characteristics of binding of L3 and D3 derivatives to the LBD of LXRα and β.

Compounds		LXRα		LXRβ
		EC50 (M)	IC50 (M)	EC50 (M)
D3 derivative	20(OH)D3	1.5 × 10^–8^ ± 7.1 × 10^–10^ (n = 2)	N/A	2.6 × 10^–6^ ± 2.7 × 10^–6^ (n = 2)
	25(OH)D3	1.1 × 10^–8^ ± 1.1 × 10^–8^ (n = 2)	N/A	3.8 × 10^–6^ ± 1.7 × 10^–6^ (n = 2)
	20,23(OH)_2_D3	1.1 × 10^–6^ ± 1.5 × 10^–6^ (n = 2)	N/A	2.6 × 10^–5^ ± 3.4 × 10^–5^ (n = 2)
	20,25(OH)_2_D3	3.5 × 10^–7^ ± 5.5 × 10^–7^ (n = 3)	N/A	2.1 × 10^–6^
	1,20,23(OH)_3_D3	N/A	2.1 × 10^–6^	1.6 × 10^–6^
	1,20,25(OH)_3_D3	N/A	2.3 × 10^–6^ ± 7.1 × 10^–8^ (n = 2)	3.8 × 10^–9^
	1,20(OH)_2_D3	N/A	3.5 × 10^–6^ ± 3.1 × 10^–6^ (n = 2)	2.7 × 10^–7^ ± 2.0 × 10^–7^ (n = 2)
	1,25(OH)_2_D3	N/A	1.1 × 10^–6^ ± 1.3 × 10^–6^ (n = 2)	1.4 × 10^–5^ ± 1.8 × 10^–5^ (n = 2)
	D3	7.6 × 10^–7^ ± 1.0 × 10^–6^ (n = 2)	N/A	8.9 × 10^–7^ ± 3.0 × 10^–7^ (n = 2)
L3 derivative	20(OH)L3	1.8 × 10^–6^ ± 2.5 × 10^–6^ (n = 2)	N/A	2.1 × 10^–8^ ± 2.4 × 10^–8^ (n = 2)
	22(OH)L3	6.4 × 10^–7^ ± 7.6 × 10^–7^ (n = 3)	N/A	1.4 × 10^–5^ ± 1.7 × 10^–5^ (n = 2)
	24(OH)L3	3.6 × 10^–6^ ± 8.5 × 10^–7^ (n = 2)	N/A	1.3 × 10^–6^ ± 7.1 × 10^–7^ (n = 2)
	20,22(OH)_2_L3	1.5 × 10^–6^ ± 1.4 × 10^–6^ (n = 2)	N/A	2.0 × 10^–6^ ± 4.2 × 10^–7^ (n = 2)
	25(OH)L3	1.4 × 10^–7^ ± 2.3 × 10^–7^ (n = 3)	N/A	1.9 × 10^–6^
	(25R)-27(OH)L3	8.2 × 10^–7^ ± 1.2 × 10^–6^ (n = 3)	N/A	5.5 × 10^–7^
	(25S)-27(OH)L3	2.6 × 10^–8^ ± 2.0 × 10^–8^ (n = 3)	N/A	1.3 × 10^–9^
	L3	2.5 × 10^–7^ ± 9.9 × 10^–8^ (n = 2)	N/A	1.1 × 10^–7^
pD derivative	17,20(OH)_2_pD	3.0 × 10^–5^ ± 3.9 × 10^–5^ (n = 2)	N/A	2.6 × 10^–6^ ± 7.1 × 10^–7^ (n = 2)

#### Ligand induced translocation of LXRα/β to the nucleus

**Figure 6:**
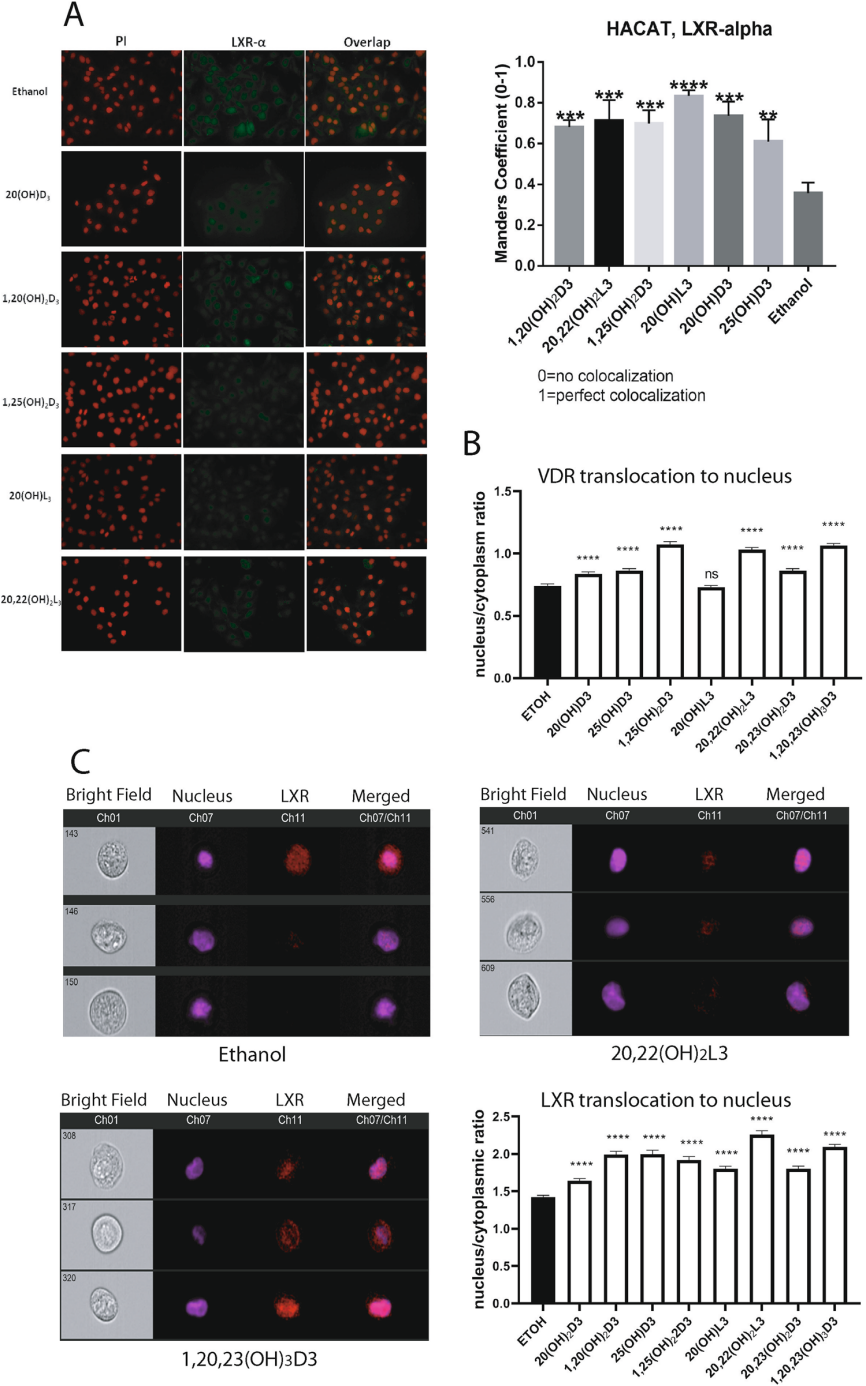
Ligand induced translocation of LXR to the nucleus. (**A**) Colocalization analysis of LXRα and PI (nuclear counterstain) in HaCaT cells treated with 10^–7^ M of 25(OH)D3, 1,25(OH)_2_D3, 20(OH)D3, 1,20(OH)_2_D3, 20(OH)L3 or 20,22(OH)_2_L3 or ethanol (control) for 24 h. Manders’ coefficient (0–1) (right panel) was significantly higher for cells treated with D3 and L3-hydroxyderivatives than cells treated with vehicle only. Data are presented as means ± SD, (n = 2). (**B**) Imaging flow cytometry analysis of HaCaT cells treated with ethanol or 10^–7^ M 20(OH)D3, 1,20(OH)_2_D3, 25(OH)D3, 1,25(OH)_2_D3, 20(OH)L3, 20,22(OH)_2_L3, 20,23(OH)_2_L3 or 1,20,23(OH)_3_D3 for 12 h. Fixed cells were stained with Hoechst and immunostained with antibodies against VDRR. Ratios of nuclear (co-localization with Hoechst) vs cytoplasmic localization of VDR were determined following analysis of 515 to 2339 individual cells. (**C**) Imaging cytometry images of individual HaCaT cells showing LXR localization in cytoplasm or nucleus following treatment with ethanol, 20,22(OH)_2_L3 or 1,20,23(OH)_3_D3. Bar graphs represent quantitative analysis of images acquired by imaging cytometry. HaCaT cells treated with ethanol or 10^–7^ M 20(OH)D3, 1,20(OH)_2_D3, 25(OH)D3, 1,25(OH)_2_D3, 20(OH)L3, 20,22(OH)_2_L3, 20,23(OH)_2_L3 or 1,20,23(OH)_3_D3 for 12 h were fixed, permeabilized cells and immunostained with Hoechst and antibodies against LXR. Ratios of nuclear vs cytoplasmic localization of LXR were determined following analysis of 515–2339 individual cells. The data in bar graphs (**A**–**C**) show significant differences between ligand -treated and control (ethanol treated) cells. Analysis was done using t-test: **p < 0.01, ***p < 0.001 or ****p < 0.0001 versus control (ethanol). For part A the slides were examined using a KEYENCE America BZ-X710 Fluorescence Microscope (Itasca, IL) and captured using KEYENCR BZ-X viewer (version 1.3.0.5, https://www.keyence.com/products/microscope/fluorescence-microscope/bz-x700/index_pr.jsp). The images were subsequently analyzed using the JACoP plugin (version 2.1.1, https://imagejdocu.tudor.lu/doku.php?id=plugin:analysis:jacop_2.0:just_another_colocalization_plugin:start) for colocalization analysis^1^ with ImageJ (version 1.52a, http://imageJ.nih.gov/ij). For part C, images were captured using an Amnis ImageStreamX Mk II Imaging Flow Cytometer (Luminex Corporation) and IDEAS software version 6.2.

To study ligand-induced translocation of LXRα to the nucleus, HaCaT keratinocytes were treated with 10^−7^ M 25(OH)D3, 1,25(OH)_2_D3, 20(OH)D3, 1,20(OH)_2_D3, 20(OH)L3 or 20,22(OH)_2_L3 or ethanol (vehicle control) for 24 h, and translocation was calculated based on the immunofluorescence stain using anti-LXRα antibodies. As shown in Fig. 6A, all compounds induced translocation of LXRα to the nucleus. As an independent approach, HaCaT keratinocytes were exposed to 20(OH)D3, 1,20(OH)_2_D3, 25(OH)D3, 1,25(OH)_2_D3, 20(OH)L3, 20,22(OH)_2_L3, 20,23(OH)_2_L3 or 1,20,23(OH)_3_D3 for 12 h, harvested, fixed and incubated with polyclonal antibodies against both LXRα and β or antibodies against VDR along Hoechst dye and analyzed by imaging flow cytometry ImageStream II. Figure 6B,C showed increased translocation of LXRα/β and VDR (which serves as a positive control for secosteroids) from the cytoplasm to the nucleus following ligands treatment.

### Investigation of LXRα and LXRβ dynamic interactions with the selected D3 and L3 derivatives using MD simulations and binding free energy analyses

The interactions of D3 hydroxyderivatives with LXR ligand binding domain (LBD) could affect secondary structure, conformation and dynamical motion changes of functionally relevant regions of LXR, further affecting LXR/RXR induced cellular activities. We performed molecular dynamics simulations to provide further insight from conformation, dynamical motion and electrostatic potential perspectives to better understand the activation mechanism of LXRs by D3 and L3 derivatives. We also performed binding thermodynamics analyses based on MD simulation trajectories to demonstrate binding specificity of D3 and L3 derivatives with LXRs.

#### Identification of LXRα and LXRβ crystal structures in complex with the selected four D3 and two L3 derivatives for MD simulations, ligand force field and MD simulation equilibration

We selected 25(OH)D3, 1,25(OH)_2_D3, 20(OH)D3 and 1,20(OH)_2_D3 for the further studies, because the first two are the products of canonical pathway of D3 activation^2^, while 20(OH)D3 is the main product of CYP11A1 action on D3^16,19,45^ and 1,20(OH)_2_D3 represents a product of CYP27B1 hydroxylation, similar to 1,25(OH)_2_D3^46^. We also included 20(OH)L3 in these detailed analysis, because of its hydroxyl group at C20, and 20,22(OH)_2_L3 as representatives of a dihydroxy-lumisterol^20,47^.

**Figure 7:**
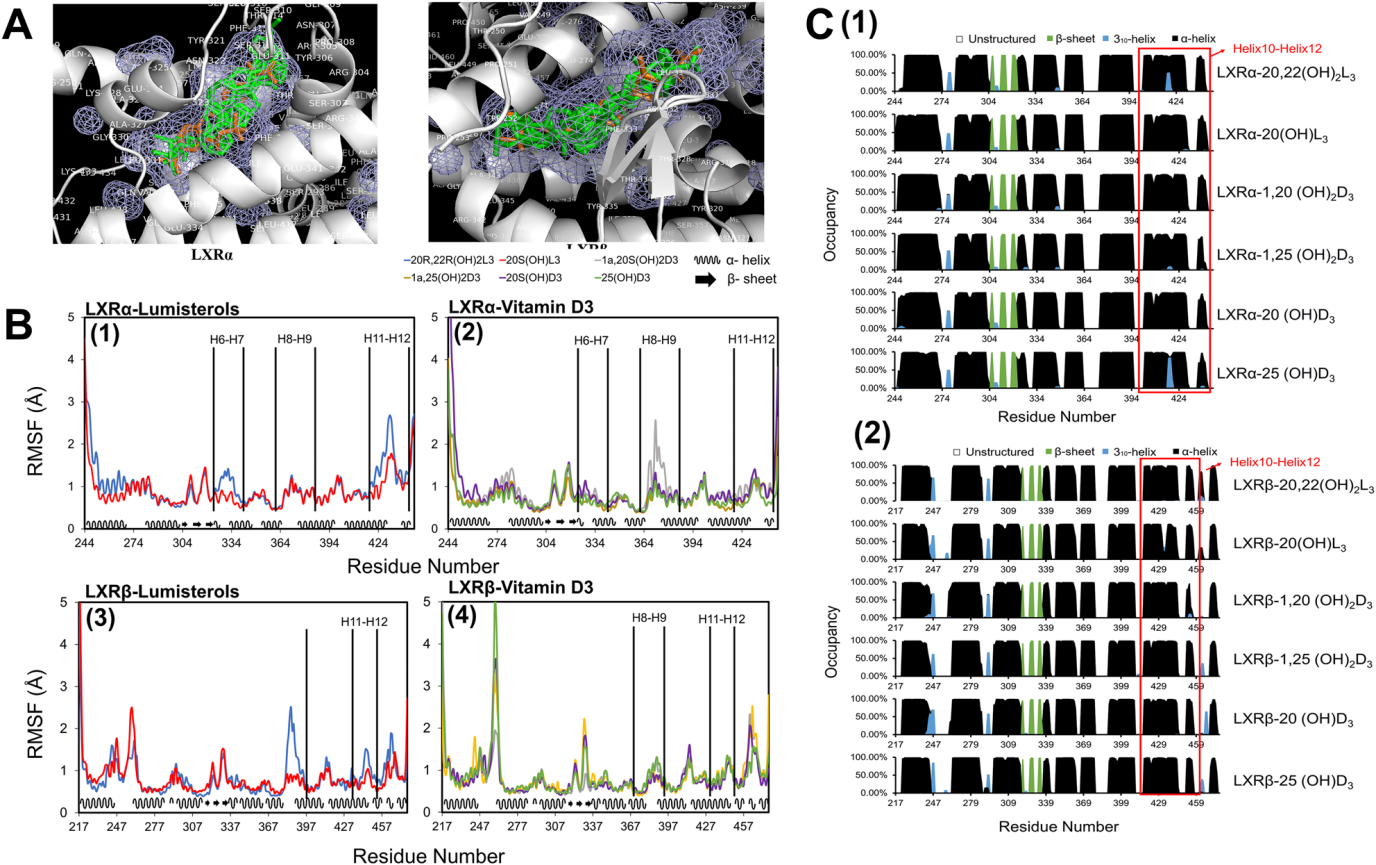
(**A**) Binding modes for the selected four D3 derivatives (1,20(OH)_2_D3, 1,25(OH)_2_D3, 20(OH)D3, and 25(OH)D3) and two L3 derivatives (20,22(OH)_2_L3, 20(OH)L3) and co-crystalized ligands in the ligand binding domain (LBD) of LXRα (PDBID:5AVI) and LXRβ (PDBID:5HJP). Docked poses of the studied ligands are shown in green and the co-crystallized ligands in LXRα and in LXRβ are shown in light brown. The mesh areas shown in the figure are hydrophobic binding regions in LXRs. (**B**,**C**) are based on last 150 ns of the equilibrated MD trajectories (**B**) Different L3 and D3 derivatives resulted in varied degrees of conformational fluctuation for the residues between helices in the LBDs of LXRα and LXRβ. (**C**) Different L3 and D3 derivatives could result in the small secondary structure changes of helix 10 to helix 12 for both LDB of LXRα and LXRβ. Image for (**A**) is made with PyMOL (v2.4.0, https://pymol.org/2/)^99^ based on our molecular docking results. Image for (**B**) is made with Microsoft Excel (v2019, https://office.microsoft.com/excel)^100^ based on the root mean square fluctuation (RMSF) analysis of our molecular dynamics simulation trajectories. Image for (**C**) is made with Microsoft Excel (v2019, https://office.microsoft.com/excel)^100^ based on the secondary structural analysis of our molecular dynamics simulation trajectories.

The comparison of the alignment parameters of the four D3 and two L3 ligands selected with co-crystalized ligands of LXRα (PDBID 5AVI, 3IPQ) and LXRβ (PDBID 5HJP, 1UPV) and the binding energy of the selected ligands with LXRα and LXRβ receptor are shown in Supplementary Tables 17 and 18. Based on alignment parameters and docking score, crystal structures of LXR $\alpha$ (PDBID 5AVI) and LXR $\beta$ (PDBID 5HJP) were chosen as receptors for the selected four D3 and two L3 compounds for the further investigations. Molecules of interest and the identified LXRα and LXRβ crystal structures are listed in Table 2. Docked poses for the selected D3 and L3 derivatives and co-crystallized ligands in ligand binding domain (LBD) of LXRα and LXRβ are shown as Fig. 7A. They aligned well with the co-crystallized ligand and share the same ligand binding pocket, further predicting ligand binding specificity with LXRs.

**Table 2:** The selected compounds and the identified crystal structures of LXRα (LBD) (PDBID 5AVI) and LXRβ (LBD) (PDBID 5HJP) for the MD studies and Glide XP docking scores for the ligands with receptors.

	Compound	Compound structure	Receptor chosen	Docking score
Vitamin D3	1^α^,20^S^(OH)_2_D3		LXRα (5AVI)	− 11.63
			LXRβ (5HJP)	− 12.144
	1^α^,25(OH)_2_D3		LXRα (5AVI)	− 11.466
			LXRβ (5HJP)	− 11.226
	20^S^(OH)D3		LXRα (5AVI)	− 10.918
			LXRβ (5HJP)	− 12.017
	25(OH)D3		LXRα (5AVI)	− 10.936
			LXRβ (5HJP)	− 11.705
Lumisterol	20^R^,22^R^(OH)_2_L3		LXRα (5AVI)	− 11.366
			LXRβ (5HJP)	− 13.028
	20^S^(OH)L3		LXRα (5AVI)	− 9.825
			LXRβ (5HJP)	− 10.632

Ligand force field and charge parameters (Supplementary Fig. S9–S14 and Supplementary Tables 19–24) and equilibration of MD simulations systems (Supplementary Fig. S15–S18) are detailed in the supplemental file. The equilibrated last 150 ns of MD simulation trajectories were used for the analyses of the binding thermodynamics of LXRα and LXRβ with the selected D3 and L3 derivatives, and the conformation and motion characteristics of LXRs binding with these ligands.

#### Binding thermodynamics analysis

Using the equilibrated last 150 ns MD simulations trajectories, we calculated the binding free energy and binding energy components of LXRα and LXRβ with the selected four D3 derivatives and two L3 derivatives (Table 3). Table 3 showed that the binding free energy of LXRα with the studied ligand: 20,22(OH)_2_L3, 20(OH)L3, 1,20 (OH)_2_D3, 1,25(OH)_2_D3, 20(OH)D3, or 25(OH)D3 was − 20.14 ± 4.40, − 19.94 ± 5.91, − 21.91 ± 4.65, − 18.15 ± 9.06, − 22.92 ± 4.09, or − 16.13 ± 4.50 kcal/mol, respectively. Table 4 showed that the binding free energy of LXRβ with the studied ligand: 20,22(OH)_2_L3, 20(OH)L3, 1,20 (OH)_2_D3, 1,25(OH)_2_D3, 20(OH)D3, or 25(OH)D3 is − 28.19 ± 3.88, − 20.87 ± 3.07, − 23.69 ± 4.30, − 27.08 ± 3.52, − 22.62 ± 6.40, or − 22.75 ± 6.22 kcal/mol, respectively. Thus, all the ligands were predicted to have favorable binding with both LXRα and LXRβ, which is consistent with the experimental binding assays (Table 1, Fig. 5). Although these ligands display favorable binding with LXRα and LXRβ, different binding energy components such as van der Waals energy, electrostatics energy and polar solvation energy were observed between different ligand in complex with LXRα and LXRβ. Different chemical structure and atomic charge parameters for the four D3 and two L3 derivatives studied (Supplementary Fig. S9–S14 and Supplementary Tables 19–24) could directly affect the interactions of each ligand with the ligand binding pocket of LXRα and LXRβ as shown in Fig. 7A. These different interactions could result in the varied hydrogen bond formation, van der Waals interactions and electrostatic interactions, further contributing to the observed different energy components for binding free energy between different ligand-LXR complexes, contributing to the observed differences in binding affinity seen experimentally (Table 1 and Fig. 5) and supporting ligand binding specificity with LXRs.

**Table 3:** Binding free energy between LXRα and D3 or L3 derivatives (Kcal/mol).

	LXRα-20,22(OH)_2_L3	LXRα-20(OH)L3	LXRα-1,20 (OH)_2_D3	LXRα-1,25 (OH)_2_D3	LXRα-20 (OH)D3	LXRα-25 (OH)D3
ΔE_vdW_	− 12.70 ± 3.11	− 13.32 ± 1.96	− 19.63 ± 3.82	− 11.72 ± 5.40	− 5.94 ± 0.31	− 11.27 ± 1.53
ΔE_electrostatic_	− 57.64 ± 1.49	− 46.15 ± 6.23	− 61.81 ± 3.01	− 56.35 ± 5.17	− 61.67 ± 2.99	− 56.62 ± 2.35
ΔG_nonpolar-solvation_	− 6.64 ± 0.11	− 5.26 ± 0.59	− 6.86 ± 0.13	− 6.92 ± 0.09	− 6.89 ± 0.11	− 6.87 ± 0.04
ΔG_polar-solvation_	33.86 ± 2.84	26.63 ± 3.62	43.55 ± 3.85	35.15 ± 4.37	28.09 ± 0.29	33.60 ± 4.02
ΔTS	− 22.50 ± 6.53	− 18.17 ± 8.13	− 22.96 ± 5.88	− 22.35 ± 6.61	− 23.56 ± 6.49	− 24.67 ± 7.24
ΔG_binding_	− 20.14 ± 4.40	− 19.94 ± 5.91	− 21.91 ± 4.65	− 18.15 ± 9.06	− 22.92 ± 4.09	− 16.13 ± 4.50

**Table 4:** Binding free energy between LXRβ and D3 or L3 derivatives (Kcal/mol).

	LXRβ-20,22(OH)_2_L3	LXRβ-20(OH)L3	LXRβ-1,20 (OH)_2_D3	LXRβ-1,25 (OH)_2_D3	LXRβ-20 (OH)D3	LXRβ-25 (OH)D3
ΔE_vdW_	− 18.34 ± 3.12	− 8.60 ± 3.78	− 21.50 ± 3.24	− 30.11 ± 2.67	− 16.43 ± 2.93	− 16.68 ± 1.05
ΔE_electrostatic_	− 58.95 ± 3.19	− 59.51 ± 2.73	− 64.54 ± 2.88	− 62.98 ± 3.11	− 61.06 ± 2.82	− 62.22 ± 2.91
ΔG_nonpolar-solvation_	− 6.59 ± 0.11	− 6.66 ± 0.11	− 6.79 ± 0.04	− 7.04 ± 0.12	− 6.99 ± 0.11	− 6.98 ± 0.10
ΔG_polar-solvation_	33.08 ± 2.00	31.03 ± 1.33	44.79 ± 2.52	49.50 ± 2.63	37.76 ± 0.38	39.35 ± 1.18
ΔTS	− 22.70 ± 5.18	− 22.91 ± 5.92	− 24.13 ± 6.11	− 23.65 ± 6.23	− 23.93 ± 11.65	− 23.80 ± 10.95
ΔG_binding_	− 28.19 ± 3.88	− 20.87 ± 3.07	− 23.69 ± 4.30	− 27.08 ± 3.52	− 22.62 ± 6.40	− 22.75 ± 6.22

All values in this table were expressed in term of Kcal/mol. ΔE_vdW_, van der Waals energy, ΔE_electrostatic_, electrostatic energy; ΔG_nonpolar-solvation_, nonpolar solvation energy; ΔG_polar-solvation_, polar solvation energy; ΔTS, energy contributed from solute entropy; ΔG_bingding_, binding free energy for the complex.

#### Effects of the selected D3 and L3 derivative on conformation, secondary structure, dynamical motion and electrostatic potential of LXRα and LXRβ

##### Hydrogen bond analyses

While the ligand binding pocket for LXRα and LXRβ is hydrophobic, there are polar or charged residues at the two ends of the cavity^48^. Table 5 shows a hydrogen bond occupancy of no less than 40% between LXRα and D3 or L3 derivatives. More than 80% hydrogen bond occupancy was predicted between 20,22(OH)_2_L3 and THR302 of LXRα. Table 6 shows the hydrogen bond occupancy of no less than 40% between LXRβ and D3 or L3 derivatives. For LXRβ-ligand complexes, more than 80% hydrogen bond occupancy was predicted between 1,20(OH)_2_D3 and SER278, between 1,20(OH)_2_D3 and HID435, between 1,25(OH)_2_D3 and HID435, between 20 (OH)D_3_ and SER278, between 20 (OH)D_3_ and HID435, between 25 (OH)D_3_ and HID435, between 20,22(OH)_2_L3 and THR316, and between 20,22(OH)_2_L3 and GLN438. Hydrogen bond occupancy of more than 10% between D3 or L3 derivatives and LXRα and LXRβ are shown in Supplementary Tables 25 and 26. The results showed that during the dynamic interactions procedure, in addition to the residues shown in Tables 5, 6, additional residues in LBD of LXRα and LXRβ were involved in forming hydrogen bond with D3 or L3 derivatives although the percentage of times that hydrogen bond exist over the equilibrated MD simulation trajectories was less than 40%. Supplementary Figs. S19 and S20 showed the 2D interaction map of D3 and L3 derivatives with ligand binding region of LXRα and LXRβ for the representative complex structure from the clustering analysis of the equilibrated MD simulation trajectories. The *dbscan* (density-based spatial clustering of applications with noise) program^49^ in Amber 14 was used for the clustering analyses of the equilibrated MD simulation trajectories, and the medoid structure in the largest cluster was chosen as representative structure. The different molecular structure of D3 or L3 derivatives leads to the different binding position and posture in the LXRα and LXRβ binding pocket, which results in different hydrophobic contacts and hydrogen bond occupancy. Thus, while the D3 and L3 derivatives studied were all shown to bind to LXRα and LXRβ (Tables 1 and 3, Fig. 5), the residues in LXRs involved with hydrogen bond formations with the ligands are predicted to be different, contributing to the observed different binding energy component, including electrostatic potential (Table 3). This provides a molecular basis to interpret the experimentally observed different binding characteristics of LXRs with D3 and L3 derivatives (Table 1, Fig. 5) and support ligand binding specificity with LXRs.

**Table 5:** Occupancy of hydrogen bond (no less than 40%) formed between LXRα and D3 or L3 derivatives.

	LXRα-20,22(OH)_2_L3	LXRα-20(OH)L3	LXRα-1,20 (OH)_2_D3	LXRα-1,25 (OH)_2_D3	LXRα-20 (OH)D3	LXRα-25 (OH)D3
LEU260			40%			
SER264			69%	54%	60%	
GLU301					42%	
THR302	84%		47%			
ARG305		43%			45%	56%
HID421		47%	78%	42%

**Table 6:** Occupancy of hydrogen bond (no less than 40%) formed between LXRβ and D3 or L3 derivatives.

	LXRβ-20,22(OH)_2_L3	LXRβ-20(OH)L3	LXRβ-1,20 (OH)_2_D3	LXRβ-1,25 (OH)_2_D3	LXRβ-20 (OH)D3	LXRβ-25 (OH)D3
THR272				57%		
LEU274			51%			
SER278		63%	86%		80%	
GLH281			70%			
GLU315				49%		
THR316	86%					
PHE329	40%	48%		57%	54%	50%
HID435			89%	82%	90%	93%
GLN438	88%					

##### Conformation and secondary structure analyses

The ligand-binding domain of LXRα and LXRβ is a three layered α-helical sandwich which includes two β-sheets (S1 and S2) and 12 helices (h1–h12)^48,50–52^. Activation function-2 (AF-2), in which helix 12 is key, is a ligand-dependent C-terminal sequence that controls LXRα and LXRβ transcriptional activity in response to ligand binding^48,50–53^. Conformational fluctuation and secondary structure changes of LXRα and LXRβ by interactions with D3 and L3 derivatives could directly affect the size and shape of the binding pocket and conformation of AF-2 region. This could further affect the binding affinity of D3 and L3 derivatives with LXRα and LXRβ as observed in Tables 1, 3 and Fig. 5, influence LXRα and LXRβ functions such as LXRs binding with coactivator for their transcriptional activity affecting gene expression (Figs. 1, 2, 3, 4).

The root mean squared fluctuation (RMSF) analysis examined the conformation stability of LXRα and LXRβ binding to different D3 and L3 derivatives (Fig. 7B). Overall, RMSF of α-helices in LXR receptors are more stable than β-sheet and the unstructured parts of LXR receptors in the complex with D3 and L3 derivatives. Conformational stability differences for LXRα and LXRβ were observed with the interactions with the different D3 and L3 derivatives. The overall structure of helices in the LBD of LXRα and LXRβ with the interactions with D3 and L3 derivatives was stable. Different degrees of variation in RMSFs for the residues between helices, including the AF-2 region, could directly affect binding affinity of LXRs with D3 and L3 derivatives (Tables 1 and 3, Fig. 5) and the motion of LXRs, further influencing its binding to coactivators for its functions and affecting gene expression (Figs. 1, 2, 3, 4).

Analysis of secondary structure by the Define Secondary Structure of Proteins (DSSP) algorithm^54^ predicted that there are not significant differences in α-helix, 3_10_-helix and β-strand secondary structure of LXRα and LXRβ between the different D3 and L3 derivatives, and the LBD of both LXRα and LXRβ is mainly formed by α-helices (Fig. 7C). The stable secondary structures are supported by the observation that there is no significant conformational fluctuation difference for α-helix and β-sheet in LXRα and LXRβ bound with different D3 and L3 derivatives (Fig. 7B). Small variations in α-helix occupancy between helices 10–12 for both LXRα and LXRβ when bound with different ligand were observed (Fig. 7C). The position of helix 12 plays a key role in the control of LXR transcriptional activity by determining the recruitment of either coactivators or corepressors^48,50–53^. The small secondary structure difference between helices 10 and 12 for LXRα and LXRβ bound with different D3 and L3 derivatives might affect this binding with coactivators as experimentally observed (Fig. 4).

##### Dynamical motion analyses

**Figure 8:**
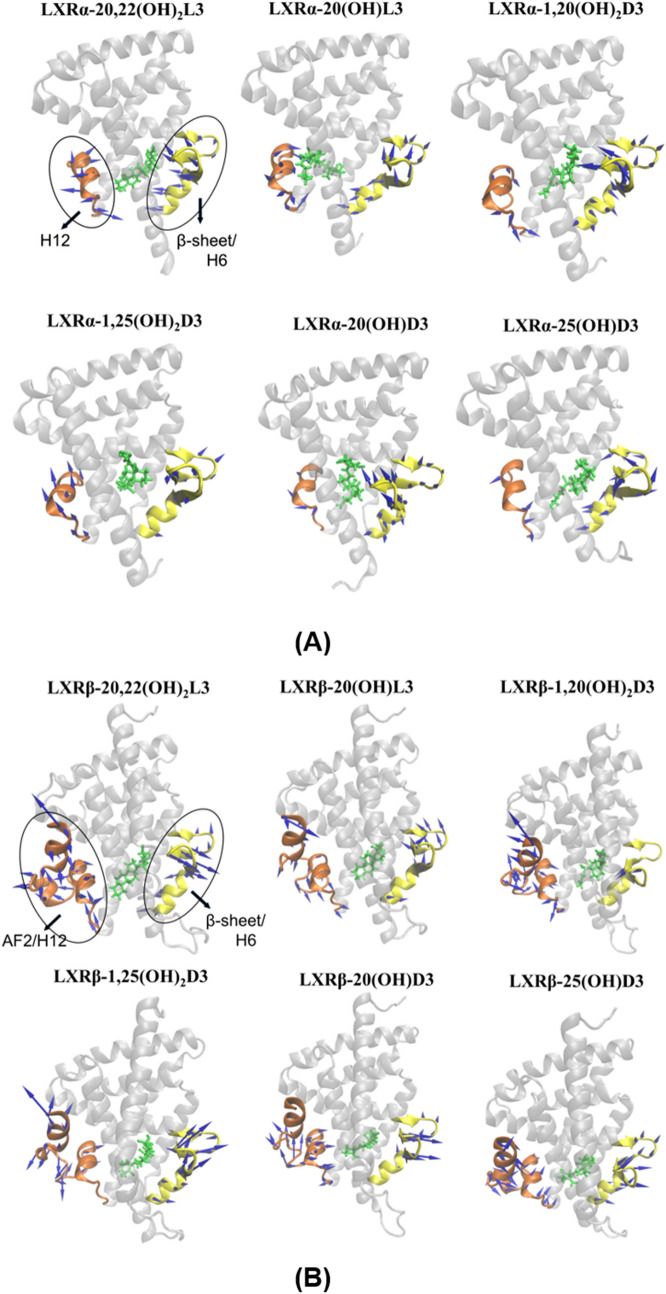
Varied principal dynamic motion of helix 12/AF-2 region (in brown color) and the β-sheet/helix 6 (in yellow color) in the ligand-binding pocket of LXRα (**A**) and LXRβ (**B**) by binding with different D3 and L3 derivatives. LXRα and LXRβ were shown as new cartoon and molecules were shown as licorice in green. Arrows for principal dynamic motion are shown as blue. The images were made with VMD (v1.9.2, http://www.ks.uiuc.edu/Research/vmd/)^101^ based on principal component analysis (PCA) of our molecular dynamics simulation trajectories.

Since LXRβ has a flexible ligand-binding pocket that can accommodate structurally different ligands^48^, we performed principal component (PCA) analysis for the LBD of LXRs to calculate the distribution of the relative contribution of the first fifty PCA modes of the LXRα (Supplementary Fig. S21) and LXRβ (Supplementary Fig. S22) in the complexes of LXRs with D3 or L3 derivatives. Results showed that the first fifty PCA modes covered almost 100% of the motion modes of LXRs, the first three PCA modes contributed significantly more to the dynamical motions of LXRs compared to other PCA modes, and the first PCA mode represented the largest motion direction of the LBD of LXRs over the equilibrated MD simulations. The ligand-binding pocket of LXRs extends from helix 12 to the β-sheet lying between helices H6 and H7^48^ and helix 12 is the key helix for the AF-2 region to bind coactivators required for LXR transcriptional activity^48,50–53^. We generated porcupine plots to show the principal dynamic motions of the helix 12/AF-2 region relative to the β-sheet/helix 6 in the ligand-binding pocket of LXRα and LXRβ by binding with different D3 and L3 derivatives. This includes the first mode of dynamical motion (Fig. 8) and the second and the third modes of dynamical motion (Supplementary Fig. S23). The porcupine arrows in Fig. 8 and Supplementary Fig. S23 represent the motion direction and the arrow length represents the magnitude of this motion. Results indicate that there is varied motion of the helix 12/AF-2 region relative to β-sheet/helix 6 for LXRs bound with different D3 and L3 derivatives demonstrating the flexible characteristics for ligand binding pocket of LXRα and LXRβ as observed in a previous study^48^. The first three principal modes of the helix 12/AF-2 region in LXRβ showed a large amplitude of motion that is consistent with the high degree of conformational fluctuation for residues in the AF-2 region, as observed in RMSF results shown in Fig. 7B. The moderate motion amplitude of β-sheets for LXRs bound with different ligands is also consistent with the fluctuation in the conformation of β-sheets observed for residues by RMSF (Fig. 7B). In addition to the principal dynamical motion of the helix 12/AF-2 region and β-sheet/helix 6 represented with porcupine arrows (Fig. 8), the dynamical motion of the other helices and unstructured regions that contributed to the binding of LXRs with ligands are shown in the movies: Movie_LXRAlpha_125OH2D3.mpeg and Movie_LXRBeta_125OH2D3.mpeg in the supplemental materials. The movies show the dynamic interaction of 1,25(OH)_2_D3 with LXRα or LXRβ during the equilibrated last 150 ns MD simulations. In the movie of the interaction of 1,25(OH)_2_D3 with LXRβ, a large motion of the unstructured region between helix 1 and helix 3 was observed, which is consistent with the observed large conformation fluctuation for the residues between helix 1 and helix 3 in Fig. 7B (4). Together with the different conformational fluctuation (Fig. 7B), the varied motions for the LBD of LXRα and LXRβ by interactions with D3 and L3 derivatives could directly influence the size and shape of the ligand binding pocket, further resulting in the different binding affinities of ligand with LXRs, as observed in Tables 1 and 3 and in Fig. 5. The different dynamic motion of the helix 12/AF-2 region of LXRs could affect LXRs binding with coactivators required for transcriptional activity of LXR and regulation of downstream gene expression, as observed in Figs. 1, 2, 3, 4.

##### Electrostatic potential analyses

**Figure 9:**
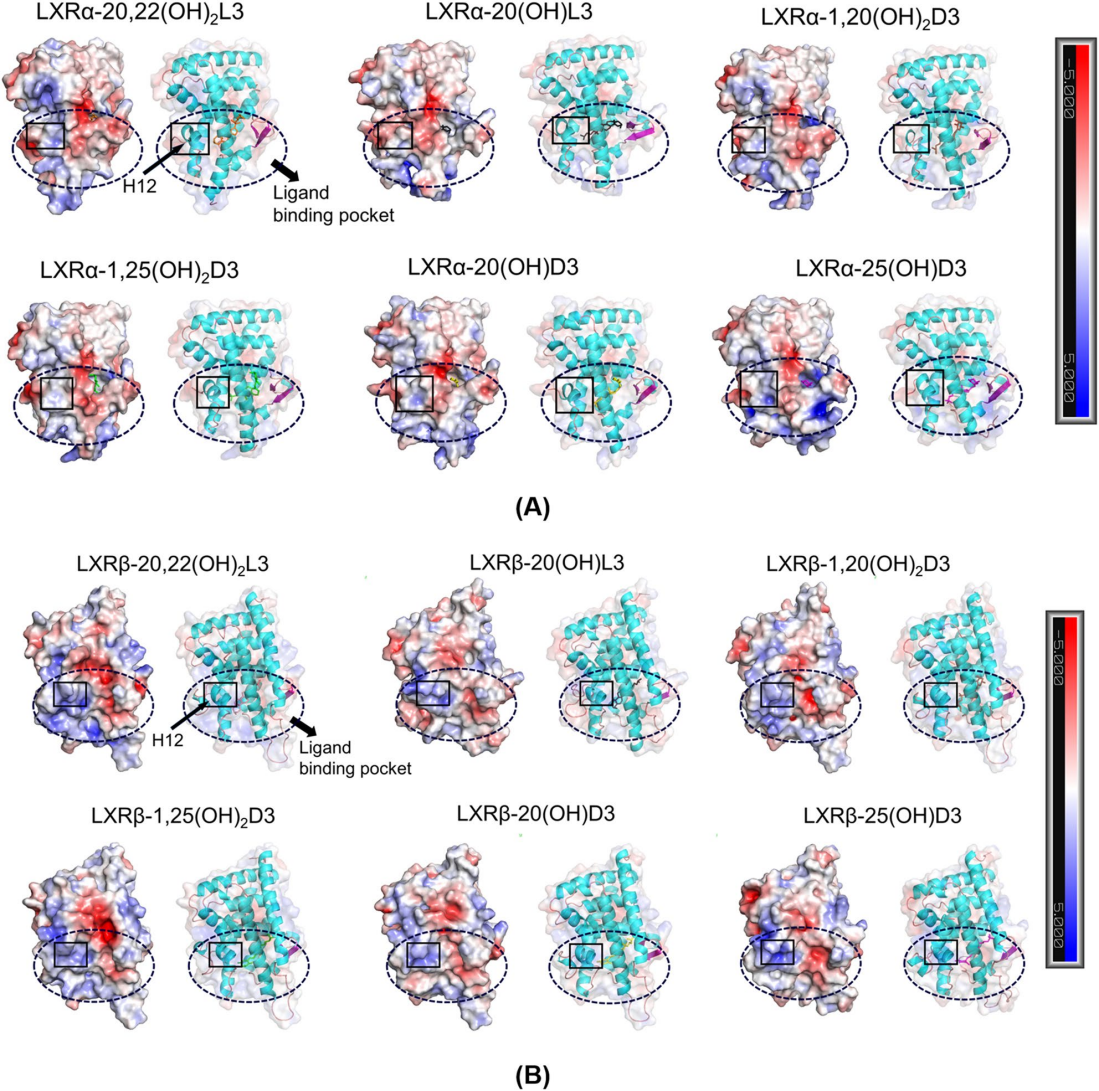
Varied electrostatic potential distribution for the LBD of LXRα (**A**) and LXRβ (**B**) bound with different D3 and L3 derivatives, including for the ligand binding pocket and H12 helix. Positive electrostatic potential is shown in blue and negative electrostatic potential is shown in red. The images were made with PyMOL(v2.4.0, https://pymol.org/2/)^99^ based on electrostatic potential analyses of the representative complex structures in our equilibrated MD simulations using APBS (v1.2.0, http://www.poissonboltzmann.org/)^98^.

Electrostatic potential analysis of the LBD of LXRα and LXRβ bound with different D3 and L3 derivatives (Fig. 9A,B) could help to understand the effect of D3 and L3 derivatives on the electrostatic potential distribution of LBD, providing a molecular insight into the binding of D3 and L3 derivatives with LXRs and with its function (Table 1, Figs. 4 and 5) from an electrostatic point of view. It is known that the ligand-binding pocket of LXRs extends from helix 12 to the β-sheet lying between helices H6 and H7^48^ and helix 12 is the key helix for the AF-2 region to bind coactivators^48,50–53^. Varied electrostatic potential distribution in the LBD of LXRα and LXRβ bound with different D3 and L3 derivatives as observed in Fig. 9A,B, including in helix 12 and ligand binding pocket, could directly affect the electrostatic energy and polar solvation energy for the complexes of LXRs with ligands. These varied electrostatic potential distribution could contribute to the different binding free energy between LXRs with ligands, as reported in Table 3, further supporting the binding of LXRs with D3 and L3 derivatives observed experimentally (Table 1 and Fig. 5). Therefore, it is expected that different electrostatic potential distributions in or near helix 12 could affect dimerization of LXRs or binding with the coactivators required for transcriptional activity, as experimentally indicated (Figs. 4, 5, 6)*.*

## Concluding remarks

Bioinformatics analyses on deposited microarray and RNAseq data identified the LXR/RXR signaling complex as a target for D3 hydroxyderivatives. This was further substantiated by stimulation of the expression of genes downstream of LXR by secosteroids and by ChIP analysis of chromatin isolated from keratinocytes treated with 1,25(OH)_2_D3 which showed a significant stimulation of LXRα/β binding to the LXRE. The expression of genes downstream of LXR was also stimulated by D3 and L3 hydroxyderivatives. Identification of LXR α/β as receptors for secosteroidal and lumisterol derivatives was confirmed and strengthened by functional assays showing: (i) the activation of the transcriptional functions of LXRα and LXR using the luciferase reporter gene containing the LXRE in CHO and HaCaT cells, (ii) binding in LanthaScreen TR-FRET LXRα and β coactivator assays, and (iii) ligand-induced translocation of LXRα/β to the nucleus. This was further supported by molecular docking with favorable docking scores and favorable binding free energy based on molecular dynamics simulations. Also docked poses for the D3 and L3 derivatives studied aligned well with the crystal structure of ligand-LXRα and LXRβ ligand binding domain complexes and indicated that they share the same ligand binding pocket. These findings allowed us to conclude that LXRα/β are indeed receptors for secosteroids and lumisterol hydroxyderivatives. In addition, molecular docking predicted derivatives of 7DHC, 7DHP, T3 and pT as potential agonists on LXRα/β, opening new possibilities for further experimental testing.

Binding thermodynamics analyses based on MD simulations demonstrated that 20,22(OH)_2_L3, 20(OH)L3, 1,20(OH)_2_D3, 1,25(OH)_2_D3, 20(OH)D3, and 25(OH)D3 favorably bind with LXRα and LXRβ, in agreement with the experimental binding assays. Different binding energy components such as van der Waals energy, electrostatics energy, and polar solvation energy were observed between the different ligands in complex with LXRα and LXRβ, which can be attributed to the different dynamic interactions of LBD of LXRs with the four D3 and two L3 derivatives studied that have the different chemical structure and atomic charge parameters. The different D3 and L3 derivatives were predicted to display different hydrogen bonding patterns with the LXRs, varied residue conformation fluctuations and dynamic motion for the LBD of LXRα or LXRβ. These changes could alter the shape, size and electrostatic potential distribution of the LBD pocket, further contributing to the different binding affinities of the D3 and L3 derivatives for LXRα or LXRβ, as experimentally observed. The varied conformation fluctuation, motion, and electrostatic potential distribution of the helix 12/AF-2 region by D3 and L3 derivatives shown as Figs. 7, 8, 9, Supplementary Figs. S21–23 and two movies, could affect the recruitment of transcriptional mediators (e.g. coactivators) and might explain the observed differences in the gene expression profiles between different derivatives. These predictions are consistent with functional and receptor binding analyses and activation of genes with LXRE, and with LXR translocation to the nucleus. However, our study has also some limitations. For example, crystallography, NMR and/or cryogenic electron microscopy studies with secosteroidal or L3 compounds are needed to define the exact nature of the ligand receptor interactions. In addition, in our future studies we plan to include ChiP-Seq and use of LXR knockouts in relation to ligand activated phenotypic activity to better define molecular and biological consequences of these interactions.

The traditional endogenous ligands for LXR are oxysterols including 22R(OH)C, 24S(OH)C, 25(OH)C, 27(OH)C and 20S(OH)C^30–32,55^. Considering their structural similarity, it is not surprising that hydroxyderivatives of L3 (9β,10α stereoisomer of 7DHC), which are produced enzymatically by the action of CYP11A1 or CYP27A1^20–22^, are able to bind and upregulate the transcriptional activity of LXRs. Further, molecular modeling results have extended the list of potential ligands for LXR to include 7DHC (photoprecursor of L3) and short side chain derivatives of L3 and 7DHC (pL and 7DHP compounds). The significance of these findings is enhanced by the recently reported biological activity of L3-hydroxyderivatives in the skin and their detection together with L3 itself in the human epidermis and serum^10,21^. Our results predict that these compounds will exert phenotypic effect via activation of LXRs in tissues expressing these receptors. This opens up exciting possibilities for studies on the phenotypic role of novel, endogenously produced LXR ligands at the local and systemic levels and on the nature of the molecular signaling, further supplemented by studies on LXR knock-out mice. We predict that molecular signaling is depend on the cell-type specific expression of nuclear receptors for L3 or related compounds, and the actual crosstalk between LXRs and RORs^10,21,23,36^.

The VDR has long been considered to be the major if not sole nuclear receptor for 1,25(OH)_2_D3^9^. This study not only breaks this dogma but also identifies its precursors and CYP11A1-derived D3-hydroxyderivatives as ligands for LXRs, which is highly significant. The identification of hydroxyderivatives of D3 as ligands for LXRs is unexpected, because of their major structural differences with sterols represented by their 9,10 secosteroidal configuration opposed to the intact B ring in 7DHC and cholesterol^2,46,56^. On the other hand, the binding pocket of LXR is large and potentially other oxysterols could fit rather easily and interact with different regions in the LXR binding pocket. Nevertheless, the A, C and D rings of D3 remain intact and the side chain is identical to cholesterol, differing only with respect to the position(s) of hydroxylation. The ligand binding pocket (LBP) of LXR is lined with mostly non-polar residues and is flexible enough to accommodate D3-hydroxyderivatives to allow the interaction of D3 hydroxyderivatives with LXRs, as predicted by molecular modelling and shown by experimental results. With respect to the VDR, the CYP27B1 mediated addition of an OH to C1α enhances selectivity of classical and non-classical hydroxy D3 compounds towards it^2,9,26,27^. However, this 1α-hydroxylation does not affect their functions as agonists on LXR, except C1α(OH)-derivatives of D3 become inverse agonists on LXRα but not β. This could be due to the different conformational fluctuation, dynamic motion and electrostatic potentials at the AF-2 region, particularly for key helices 12, in LXRα and LXRβ by binding D3 derivatives, which could result in the different transcription activities for LXRα and LXRβ. LXRs binds RXR to form heterodimer for transcriptional activities^30–32^. Conformational changes of LXRs by binding with different D3 derivatives could result in the ligand dependent conformational changes of the LXR-RXR heterodimer, which could create a binding surface for interaction with either a co-activator or a co-repressor peptide. Whether LXR-RXR heterodimer recruits a co-activator/repressor peptide also depends on the tissue type and the abundance of the co-activator/repressor peptides. Here, we speculate that C1α(OH)-derivatives of D3 influences the protein surface to recruit a co-repressor peptide rather than co-activator peptide. Future crystallography studies, will provide further insight into this phenomenon. Additionally, the identification of tachysterols as potential ligands by molecular modeling opens exciting areas of research for their signaling and functional activities in the human epidermis^10^.

Since D3 is produced and activated in the epidermis^2,9,10^, the local concentrations of different D3-hydroxyderivatives will be very high promoting their action on multiple nuclear receptors including VDR, LXR, RORs and AhR^10^. With respect to LXR, it is expressed in the epidermis and plays an important role in the epidermal barrier functions^40,41,57,58^. Similar considerations apply to orally delivered D3 and its metabolites, which will reach high concentration in the gastrointestinal tract (GI) and liver that will include not only production 25(OH)D3 but also of CYP11A1-derived D3-derivatives, since this enzyme is expressed in GI mucosa and by cells in the immune system^59^. The future challenge is to define intra-organ selectivity of nuclear receptors activation by different D3-hydroxyderivatives to achieve the phenotypic effects and the crosstalk between LXR and RORs and perhaps VDR and AhR^10,23,36^.

In conclusion, endogenously produced L3, D3 and most likely also 7DHC and T3 derivatives are identified as ligands for LXRs opening new paradigms on their mechanisms of action, biological functions, and opening diverse opportunities for medicinal chemistry and pharmacology relating to these compounds.

## Materials and methods

### Source of compounds tested

Vitamin D3, lumisterol, 1,25(OH)_2_D3, 25(OH)D3 and 20(OH)C were purchased from Sigma-Aldrich (St. Louis, MO, USA). Hydroxy-derivatives of D_3_ and L_3_ were enzymatically synthesized and purified as previously described^20,22,45,60–65^. In addition, 20(OH)D3, 20(OH)L3 and 17,20(OH)_2_pD were chemically synthesized and purified as previously described^18,47,66^.

### Animal and primary cells protocols

All animal studies followed guidelines outlined by the NIH Guide for the Care and Use of Laboratory Animals and protocols were approved by the Institutional Animal Care and Use Committee at the NIEHS or the UAB. The study was carried out in compliance with the ARRIVE guidelines (http://www.nc3rs.org.uk/page.asp?id=1357). Skin samples from newborn C57BL/6 mice were collected at the NIEHS. Dermal fibroblasts were isolated from the skin of newborn mice, 1–2 day old. Briefly, we followed the protocols described previously^67^. The skin was washed in PBS, cut into smaller pieces and incubated in 2.4 U/ml of dispase II (Roche) overnight at 4 °C. The dermis was separated from the epidermis and incubated in 0.1% collagenase (Roche) at 4 °C overnight. Next day, it was cut into smaller pieces and incubated for 1 h in 0.25% Trypsin (CellGrow) at 37 °C. Single cell suspension was prepared by pipetting and draining through a cell strainer. After centrifugation, cell pellets were suspended in DMEM medium containing 10% charcoal-stripped fetal bovine serum (cFBS), mouse fibroblasts growth factor (FGF) and 1% antibiotic, and inoculated into tissue culture dishes (Midwest Scientific, TPP). Cells, in early passages, from different mice were pooled together to provide representative samples for experiments. Fibroblasts were cultured in Petri dishes (10 cm in diameter) in DMEM and 10% cFBS and triplicate semi-confluent cultures were treated as described.

HaCaT keratinocytes were cultured as described previously^68^. For RNA isolation the cells were maintained in TPP tissue culture petri dishes (Ø60 mm, 22.1 cm^2^) in DMEM containing 10% cFBC to reach semi-confluence and then exposed to 10^–7^ M compounds or a corresponding concentration of ethanol solvent.

Adult hairless mice SKH-1 (Male, 16 weeks old) and Ptch^**+/−**^/SKH-1 (Male, 18 weeks old) were injected subcutaneously with 20 µg and 10 µg/kg of 20(OH)D3, respectively, dissolved in ethanol, further diluted in propylene glycol (PG) and saline (0.9%) sequentially (1:19:80, v/v/v). Controls were injected with the vehicle without the drug. )After 6 h the animals were killed by halothane followed by cervical dislocation and brains were collected for RNA and protein extraction protocols. The procedures followed the guidelines and approvals of the Institute Animal Care and Use Committee of the University of Alabama at Birmingham (IACUC).

### RNAseq preparation and analyses

Triplicate cultures of murine fibroblasts were used for RNA isolation (RNAeasy Micro kit, Qiagen), the RNA samples were combined for each condition and at least 200 ng of RNA for each sample was used to create libraries for RNA sequencing by BGI Americas Corporation Service (Cambridge, MA)^67^. Quality of RNA and its concentration were determined using Agilent 2100.

The raw sequence FASTQ files were aligned to Gencode’s GRCm38 p4 Release M11 genome using STAR version 2.5.4b (parameters used: –outReadsUnmapped Fastx; –outSAMtype BAM SortedByCoordinate; –outSAMattributes All; –outFilterIntronMotifs RemoveNoncanonicalUnannotated)^69^. The abundance of transcripts was calculated from the alignments using Cufflinks version 2.2.1 (parameters used: –G; –L; –library-type fr-firststrand). Samples were merged using default parameters with Cuffmerge^70,71^. The differential expression was calculated using Cuffdiff’s default parameters. The raw data have been deposited at the NCBI GEO (GSE145818).

### Pathway analysis using ingenuity

Data was analyzed using the Ingenuity Pathway Analysis (Ingenuity Systems, www.ingenuity.com)^72^. A data set containing gene identifiers and corresponding expression values was uploaded into the application to generate networks. The identifiers were mapped to their corresponding objects in Ingenuity’s Knowledge Base. A cutoff of ± 2 of fold change was used to identify molecules whose expression levels were significantly regulated. These molecules, called Network Eligible molecules, were overlaid onto a global molecular network developed from information contained in Ingenuity’s Knowledge Base. Based on their connectivity Networks of Network Eligible Molecules were algorithmically generated. The Functional Analysis identify the biological functions or/and diseases that are most significant to the entire data set. Molecules from the dataset that meet the fold change cutoff of ± 2 and are associated with biological functions or/and diseases in Ingenuity’s Knowledge Base are used for the analysis. Right-tailed Fisher’s exact test is used to calculate the p-value determining the probability that each biological function or/and disease assigned is due to chance alone.

### Quantitative RT-PCR

RNA isolated from either fibroblasts, HaCaT cells or brain was submitted for cDNA synthesis (High Capacity cDNA Reverse Transcription Kit with RNase Inhibitor, Applied Biosystems) following the manufacturers’ protocols. RT-PCR was carried out using Cyber green, in triplicates; as previously described^73^. Approximately, 750 ng of cDNA was used for PCR reactions. GAPDH and CIC-B were used as internal controls. Primer sequences are listed in Supplementary Table 27. Data were analyzed using GraphPad Prism statistical software one way ANOVA or t-test, where appropriate; *p < 0.05; **p < 0.01; ***p < 0.001.

### ChIP assay

We followed protocols described in Ref.^40^. Briefly, HaCaT keratinocytes were grown in DMEM plus 5% cFBS to achieve 90% confluence and then treated with 10^–7^ M 1,25(OH)_2_D3 or EtOH [0.1%] for 24 h. The cells were harvested and in vivo crosslinking, lysis and sonication of the chromatin, as well as following steps of ChIP assay, were done using Magna ChiP G Kit 0002 (Millipore). The chromatin fragments were immunoprecipitated with mouse monoclonal antibody against LXRα/β (cat no. sc-377260 X, Santa Cruz) and followed the protocols of the Magna ChiP G Kit 0002. The purified DNA fragments were PCR amplified with the following sequence of PCR primers for human *ABCA1* LXR-responsive element (LXRE):

Forward: 5′-CTCAACGCCCGGGAGAAAACAG-3′, Reverse: 5′-CTCCGCCGCGGAGGTTACTA-3′ and for human GAPDH as a reference gene: Forward: 5′-AGCCACATCGCTCAGACAC-3′, Reverse: 5′-GCCCAATACGACCAAATCCC-3′ (Eurofins Genomics). The PCR reaction was performed using Dream Taq Green PCR Master Mix (2×) (Thermo Scientific, cat. no. K1081) according to the following procedure: initial denaturation: 95 °C for 3 min; denaturation: 95 °C for 30 s (× 35 cycles); annealing: 58 °C for 30 s (× 35 cycles in the same cycles with denaturation); extension: 72 °C for 1 min; final extension: 72 °C for 15 min. The detection of amplification product was done by standard agarose gel electrophoresis with EtBr using Invitrogen iBright CL1000 Imaging System, with # SM Gene Ruler 1 kb Plus DNA Ladder (Thermo Scientific) as a marker.

### LXR binding assay

Binding assays of graded concentrations of vitamin D3 or lumisterol hydroxyderivatives to LXRα/β were performed using the LanthaScreen TR-FRET LXRα/β Coactivator kit (Thermo Fisher Scientific, Inc., Waltham, MA) following the manufacture’s protocol. Briefly, after addition of LXRα/β-LBD to the derivatives, a mixture of peptide (Fluorescein-TRAP220/DRIP-2 for LXRα or Fluorescein-D22 for LXRβ) and antibody (Tb-anti-GST) was added to the reaction followed by incubation in room temperature for 2 h. TR-FRET ratio was calculated by dividing the emission at 520 nm by the emission at 495 nm using Synergy neo2 (BioTek, Winooski, VT).

### Luciferase assays

The effects of vitamin D3 and L3 hydroxyderivatives were tested on the LXRE transcriptional activity using pGreenFire1-LXRE-in-ApoE Lentivirus (System Biosciences, Palo Alto, CA). This plasmid was transduced in CHO and HaCaT cells following the manufacture’s protocol and the cells used as a stable LXRE expressed tool. Briefly, the virus was transduced into the cells using TransDux MAX Lentivirus Transduction Enhancer (System Biosciences, Palo Alto, CA) and screened to select the transduced cells using puromycin. The transduced cells were treated with the compounds for 24 h in media containing 5% cFBS (charcoal-treated fetal bovine serum) and the luminescence was read by ONE-Glo Luciferase Assay System (Promega, Madison, WI) using Cytation 5 (BioTek, Winooski, VT).

### Ligand induced LXR translocation to the nucleus

HaCaT cells were plated in DMEM plus 5% cFBS either on a sterile glass cover slip placed in a well of a 6-well plates for immunofluorescence (IF) assays or TPP Petri dishes for image-stream flow cytometry, and treated with 10^–7^ M D3- or L3-hydroxyderivatives or 0.1% ethanol (vehicle) as a negative control was added to each well, and the cells were incubated for 12 or 24 h at 37 °C with 5% CO_2_. For IF the cells were fixed in 4% paraformaldehyde for 15 min, incubated in 0.5% Triton X-100 in phosphate-buffered saline (PBS) for 5 min, and washed 3 times in PBS. After blocking with 2% bovine serum albumin (BSA) in PBS for 1 h at room temperature, the cells were treated with an anti-LXRα antibody (Novus Biologicals, LLC, Centennial, CO) at 1:200 in PBS supplemented with 2% BSA overnight in 4 °C. After washing the slides were incubated with a mouse anti-rabbit IgG-FITC (Santa Cruz Biotechnology, Inc, Dallas, TX) at 1:200 dilution for 1.5 h at 37 °C. After washing the slides were mounted using propidium iodide (PI) (Vector Labs, Burlingame, California) as a nuclear counterstain. The slides were examined using a KEYENCE America BZ-X710 Fluorescence Microscope (Itasca, IL). The images were subsequently analyzed using the JACoP plugin for colocalization analysis with ImageJ^74^. Five separate high-power field (40×) images were converted to grayscale and analyzed for each condition. Manders’ coefficient (M1), calculated using a preset threshold, was used to quantify the degree of colocalization between PI and LXRα with 0 representing no colocalization, and 1 representing perfect colocalization^75^.

For Image Stream II (Amnis, Seattle, WA, USA) cytometer analyses^76^, HaCaT cells were detached and processed as previously described^77^. The cells were fixed with paraformaldehyde and permeabilized in methanol containing buffer^78,79^ and Hoechst stain (r37165, ThermoFisher) and rabbit antibodies to LXRα/β (sc 377260, AF 647, SantaCruz) or VDR (sc-13133, AF 488, SantaCruz) as described previously^76^. Data were analyzed using IDEAS software (Amnis, Seattle, WA, USA).

### Molecular docking and modeling studies on ligands targeting LXRα and LXRγ

#### Molecular docking

To predict binding poses of a series of vitamin D3 and lumisterol derivatives towards human LXRα and LXRβ, a receptor-based approach (molecular docking) was used. Glide in Extra Precision (XP) implemented in Schrödinger (version 2016-1) was used for the docking studies. Taking into account alternative ligand binding modes that could come out of exploring different conformations of the active site, among LXR crystal structures in Protein Data Bank^80^, two LXRα (PDBID 5AVI, 3IPQ) and three LXRβ (PDBID 5HJP, 1PQC, 1UPV) with most structurally diverse conformations were selected for the docking studies, respectively, by structural clustering and visual inspection. The chosen five LXR conformations were energy minimized with OPLS2005 force field. 3D structures of the 84 ligands tested which included a D3 series (full-length side chain), pregnacalciferol (pD)(short side chain), L3, pregnalumisterol (pL) series, 7DHCseries, 7DHP series, T3 and pregnatachysterol (pT) series and natural ligands (controls) including 20(OH)C and 22(OH)C were prepared using LigPrep utility of Schrödinger at pH 7.0 and with OPLS2005 force field. Chiralities were taken into account and assigned correctly based on the chemical structure of each ligand. The 84 ligands were separately docked into each energy minimized structure of the ligand binding domain (LBD) of LXRα and LXRβ. During molecular docking, receptor grids were constructed without assigning any constraints, such as hydrogen bonds or excluded volumes, in order to allow the docking algorithm to explore maximum positions and orientations of the docked ligands. The ligand structures were set as fully flexible while the proteins remained rigid; however, rotatable groups in the residues that could possibly make contacts with the docked ligands were allowed. Docking scores were determined to evaluate the binding of each compound tested with LXRα and LXRβ (see Supplementary Information).

#### Molecular dynamics (MD) simulation and binding free energy analyses

With the selected four D3 and two L3 derivative compounds in complex with LXRα or LXRβ, we performed a total of twelve 300 ns MD simulations using the Amber 14 MD simulation package (https://ambermd.org/)^81,82^ to determine the binding mode and binding free energy of LXRs with the selected D3 and L3 derivatives, and determine the effect of the D3 and L3 derivative on conformation, secondary structure, dynamical motion and electrostatic potential distribution of LXRα and LXRβ. AMBER force field was used for the simulated systems. A standard MD simulation protocol was performed, as used in our previous studies^83–93^ for twelve simulated systems as shown in Table 2: LXRα and LXRβ with four D3 and two L3 derivatives separately. Binding free energies were calculated for the twelve complexes with the Molecular Mechanics Poisson-Boltzmann Surface Area (MM-PBSA) method as described previously^87,94–96^ which was implemented using the AMBER 14 MD Software Package.

To determine the simulated systems’ equilibration tendencies and its convergence, root mean square deviation (RMSD) of protein backbone atoms over time and binding free energy of the complex structure over the cumulative time were analyzed. Based on equilibrated MD simulation trajectories, we performed a series of analyses to better understand hydrogen bond formation, conformational stability, secondary structure and dynamical motion and electrostatic potential characteristic of LXRα and LXRβ binding with the selected D3 or L3 derivatives, which were performed using the *ptraj* program of AMBER 14. Electrostatic potential analyses were performed using APBS (v1.2.0, http://www.poissonboltzmann.org/)^97,98^. Details for determining ligand force field including the charge parameters of ligands are described in Supplementary Information section on Molecular Modelling. We also detailed MD simulation protocols, MM-PBSA binding free energy method and the analyses of hydrogen bond formation, conformation, secondary structure, dynamical motion and electrostatic potentials in Supplementary Information: Molecular Modelling.

## Supplementary Information

Supplementary Video 1.

Supplementary Video 2.

Supplementary Information.

## Data Availability

Microarray data is deposited at the NCBI GEO (GSE117351), while RNAseq data at NCBI GEO (GSE145818). The authors will share reagents on reasonable request or provide guidance for synthesis of the ligands.

## References

[1] Holick MF, Clark MB (1978). The photobiogenesis and metabolism of vitamin D. Fed. Proc..

[2] Holick MF (2003). Vitamin D: A millenium perspective. J. Cell Biochem..

[3] Bikle DD (2011). Vitamin D: An ancient hormone. Exp. Dermatol..

[4] MacLaughlin JA, Anderson RR, Holick MF (1982). Spectral character of sunlight modulates photosynthesis of previtamin D3 and its photoisomers in human skin. Science.

[5] Holick MF (2007). Vitamin D deficiency. N. Engl. J. Med..

[6] Bikle DD (2010). Vitamin D: Newly discovered actions require reconsideration of physiologic requirements. Trends Endocrinol. Metab..

[7] Christakos S, Dhawan P, Verstuyf A, Verlinden L, Carmeliet G (2016). Vitamin D: Metabolism, molecular mechanism of action, and pleiotropic effects. Physiol. Rev..

[8] Rybchyn MS (2018). Enhanced repair of UV-induced DNA damage by 1,25-dihydroxyvitamin D3 in skin is linked to pathways that control cellular energy. J. Investig. Dermatol..

[9] Bikle D, Christakos S (2020). New aspects of vitamin D metabolism and action—addressing the skin as source and target. Nat. Rev. Endocrinol..

[10] Slominski AT (2020). Photoprotective properties of vitamin D and lumisterol hydroxyderivatives. Cell Biochem. Biophys..

[11] Christakos S, Li S, De La Cruz J, Bikle DD (2019). New developments in our understanding of vitamin metabolism, action and treatment. Metabolism.

[12] Carlberg C (2018). Vitamin D genomics: From in vitro to in vivo. Front. Endocrinol..

[13] Haussler MR (2013). Molecular mechanisms of vitamin D action. Calcif. Tissue Int..

[14] Thompson PD (2001). Distinct retinoid X receptor activation function-2 residues mediate transactivation in homodimeric and vitamin D receptor heterodimeric contexts. J. Mol. Endocrinol..

[15] Jurutka PW (2005). Molecular and functional comparison of 1,25-dihydroxyvitamin D3 and the novel vitamin D receptor ligand, lithocholic acid, in activating transcription of cytochrome P450 3A4. J. Cell. Biochem..

[16] Slominski AT (2012). In vivo evidence for a novel pathway of vitamin D(3) metabolism initiated by P450scc and modified by CYP27B1. FASEB J..

[17] Slominski AT (2015). Detection of novel CYP11A1-derived secosteroids in the human epidermis and serum and pig adrenal gland. Sci. Rep..

[18] Zmijewski MA (2009). Photo-conversion of two epimers (20R and 20S) of pregna-5,7-diene-3beta, 17alpha, 20-triol and their bioactivity in melanoma cells. Steroids.

[19] Guryev O, Carvalho RA, Usanov S, Gilep A, Estabrook RW (2003). A pathway for the metabolism of vitamin D3: Unique hydroxylated metabolites formed during catalysis with cytochrome P450scc (CYP11A1). Proc. Natl. Acad. Sci. U. S. A..

[20] Tuckey RC (2014). Lumisterol is metabolized by CYP11A1: Discovery of a new pathway. Int. J. Biochem. Cell Biol..

[21] Slominski AT (2017). Characterization of a new pathway that activates lumisterol in vivo to biologically active hydroxylumisterols. Sci. Rep..

[22] Tuckey RC (2018). CYP27A1 acts on the pre-vitamin D3 photoproduct, lumisterol, producing biologically active hydroxy-metabolites. J. Steroid Biochem. Mol. Biol..

[23] Jetten AM, Takeda Y, Slominski A, Kang HS (2018). Retinoic acid-related Orphan Receptor gamma (RORgamma): Connecting sterol metabolism to regulation of the immune system and autoimmune disease. Curr. Opin. Toxicol..

[24] Bikle DD (2020). Vitamin D: Newer concepts of its metabolism and function at the basic and clinical level. J. Endocr. Soc..

[25] Kim TK (2012). Correlation between secosteroid-induced vitamin D receptor activity in melanoma cells and computer-modeled receptor binding strength. Mol. Cell. Endocrinol..

[26] Slominski AT (2017). Endogenously produced nonclassical vitamin D hydroxy-metabolites act as "biased" agonists on VDR and inverse agonists on RORalpha and RORgamma. J. Steroid Biochem. Mol. Biol..

[27] Lin Z (2018). Investigation of 20S-hydroxyvitamin D3 analogs and their 1alpha-OH derivatives as potent vitamin D receptor agonists with anti-inflammatory activities. Sci. Rep..

[28] Slominski AT (2014). RORalpha and ROR gamma are expressed in human skin and serve as receptors for endogenously produced noncalcemic 20-hydroxy- and 20,23-dihydroxyvitamin D. FASEB J..

[29] Slominski AT (2018). Differential and overlapping effects of 20,23(OH)(2)D3 and 1,25(OH)(2)D3 on gene expression in human epidermal keratinocytes: Identification of AhR as an alternative receptor for 20,23(OH)(2)D3. Int. J. Mol. Sci..

[30] Peet DJ, Janowski BA, Mangelsdorf DJ (1998). The LXRs: A new class of oxysterol receptors. Curr. Opin. Genet. Dev..

[31] El-Gendy BEM, Goher SS, Hegazy LS, Arief MMH, Burris TP (2018). Recent advances in the medicinal chemistry of liver X receptors. J. Med. Chem..

[32] Jakobsson T, Treuter E, Gustafsson JA, Steffensen KR (2012). Liver X receptor biology and pharmacology: New pathways, challenges and opportunities. Trends Pharmacol. Sci..

[33] Janowski BA (1999). Structural requirements of ligands for the oxysterol liver X receptors LXRalpha and LXRbeta. Proc. Natl. Acad. Sci. U. S. A..

[34] Svensson S (2003). Crystal structure of the heterodimeric complex of LXRalpha and RXRbeta ligand-binding domains in a fully agonistic conformation. EMBO J..

[35] Endo-Umeda K (2017). 1alpha-Hydroxy derivatives of 7-dehydrocholesterol are selective liver X receptor modulators. J. Steroid Biochem. Mol. Biol..

[36] Wada T, Kang HS, Jetten AM, Xie W (2008). The emerging role of nuclear receptor RORalpha and its crosstalk with LXR in xeno- and endobiotic gene regulation. Exp. Biol. Med. (Maywood).

[37] Lee SD, Tontonoz P (2015). Liver X receptors at the intersection of lipid metabolism and atherogenesis. Atherosclerosis.

[38] Cummins CL (2006). Liver X receptors regulate adrenal cholesterol balance. J. Clin. Investig..

[39] Viscarra J, Sul HS (2020). Epigenetic regulation of hepatic lipogenesis: Role in hepatosteatosis and diabetes. Diabetes.

[40] Na TY (2017). The trisaccharide raffinose modulates epidermal differentiation through activation of liver X receptor. Sci. Rep..

[41] Hyter S, Indra AK (2013). Nuclear hormone receptor functions in keratinocyte and melanocyte homeostasis, epidermal carcinogenesis and melanomagenesis. FEBS Lett..

[42] Schmuth M, Moosbrugger-Martinz V, Blunder S, Dubrac S (1841). Role of PPAR, LXR, and PXR in epidermal homeostasis and inflammation. Biochim. Biophys. Acta.

[43] Fowler AJ (2003). Liver X receptor activators display anti-inflammatory activity in irritant and allergic contact dermatitis models: Liver-X-receptor-specific inhibition of inflammation and primary cytokine production. J. Investig. Dermatol..

[44] Slominski AT (2018). On the role of classical and novel forms of vitamin D in melanoma progression and management. J. Steroid Biochem. Mol. Biol..

[45] Slominski A (2005). The cytochrome P450scc system opens an alternate pathway of vitamin D3 metabolism. FEBS J..

[46] Tuckey RC, Cheng CYS, Slominski AT (2019). The serum vitamin D metabolome: What we know and what is still to discover. J. Steroid Biochem. Mol. Biol..

[47] Li W (2010). Chemical synthesis of 20S-hydroxyvitamin D3, which shows antiproliferative activity. Steroids.

[48] Farnegardh M (2003). The three-dimensional structure of the liver X receptor beta reveals a flexible ligand-binding pocket that can accommodate fundamentally different ligands. J. Biol. Chem..

[49] Ester, M., Kriegel, H.-P., Sander, J. & Xu, X. In *Proceedings of 2nd International Conference on Knowledge Discovery and Data Mining* 226–231 (1996).

[50] Fradera X (2010). X-ray structures of the LXRα LBD in its homodimeric form and implications for heterodimer signaling. J. Mol. Biol..

[51] Svensson S (2003). Crystal structure of the heterodimeric complex of LXRα and RXRβ ligand-binding domains in a fully agonistic conformation. EMBO J..

[52] Williams S (2003). X-ray crystal structure of the liver X receptor β ligand binding domain regulation by a histidine-tryptophan switch. J. Biol. Chem..

[53] Wójcicka, G., Jamroz-Wiśniewska, A., Horoszewicz, K. & Bełtowski, J. Liver X receptors (LXRs). Part I: Structure, function, regulation of activity, and role in lipid metabolism Receptory wątrobowe X (LXR). Część I: Budowa, funkcja, regulacja aktywności i znaczenie w metabolizmie lipidów. *Postepy Hig. Med. Dosw. (online)* **61**, 736–759 (2007). 18063918

[54] Kabsch W, Sander C (1983). Dictionary of protein secondary structure: Pattern recognition of hydrogen-bonded and geometrical features. Biopolymers.

[55] Laffitte BA (2001). LXRs control lipid-inducible expression of the apolipoprotein E gene in macrophages and adipocytes. Proc. Natl. Acad. Sci. U. S. A..

[56] Prabhu AV, Luu W, Li D, Sharpe LJ, Brown AJ (2016). DHCR7: A vital enzyme switch between cholesterol and vitamin D production. Prog. Lipid Res..

[57] Schmuth M (2004). The effect of LXR activators on AP-1 proteins in keratinocytes. J. Investig. Dermatol..

[58] Bhattacharya N, Sato WJ, Kelly A, Ganguli-Indra G, Indra AK (2019). Epidermal lipids: Key mediators of atopic dermatitis pathogenesis. Trends Mol. Med..

[59] Slominski RM (2020). Extra-adrenal glucocorticoid biosynthesis: Implications for autoimmune and inflammatory disorders. Genes Immun..

[60] Tuckey RC (2011). Production of 22-hydroxy metabolites of vitamin d3 by cytochrome p450scc (CYP11A1) and analysis of their biological activities on skin cells. Drug Metab. Dispos..

[61] Tang EK (2013). Hydroxylation of CYP11A1-derived products of vitamin D3 metabolism by human and mouse CYP27B1. Drug Metab. Dispos..

[62] Tieu EW (2012). Rat CYP24A1 acts on 20-hydroxyvitamin D(3) producing hydroxylated products with increased biological activity. Biochem. Pharmacol..

[63] Tang EK (2010). Purified mouse CYP27B1 can hydroxylate 20,23-dihydroxyvitamin D3, producing 1alpha,20,23-trihydroxyvitamin D3, which has altered biological activity. Drug Metab. Dispos..

[64] Tuckey RC (2008). Pathways and products for the metabolism of vitamin D3 by cytochrome P450scc. FEBS J..

[65] Tieu EW (2012). Metabolism of cholesterol, vitamin D3 and 20-hydroxyvitamin D3 incorporated into phospholipid vesicles by human CYP27A1. J. Steroid Biochem. Mol. Biol..

[66] Chen J (2014). Novel vitamin D analogs as potential therapeutics: Metabolism, toxicity profiling, and antiproliferative activity. Anticancer Res..

[67] Janjetovic Z (2020). Antifibrogenic activities of CYP11A1-derived vitamin D3-hydroxyderivatives are dependent on RORγ. Endocrinology.

[68] Slominski AT (2015). Novel non-calcemic secosteroids that are produced by human epidermal keratinocytes protect against solar radiation. J. Steroid Biochem. Mol. Biol..

[69] Dobin A (2013). STAR: Ultrafast universal RNA-seq aligner. Bioinformatics.

[70] Trapnell C (2012). Differential gene and transcript expression analysis of RNA-seq experiments with TopHat and Cufflinks. Nat. Protoc..

[71] Trapnell C (2010). Transcript assembly and quantification by RNA-Seq reveals unannotated transcripts and isoform switching during cell differentiation. Nat. Biotechnol..

[72] Kramer A, Green J, Pollard J, Tugendreich S (2014). Causal analysis approaches in Ingenuity Pathway Analysis. Bioinformatics.

[73] Janjetovic Z (2014). Melatonin and its metabolites ameliorate ultraviolet B-induced damage in human epidermal keratinocytes. J. Pineal Res..

[74] Bolte S, Cordelieres FP (2006). A guided tour into subcellular colocalization analysis in light microscopy. J. Microsc..

[75] Zinchuk V, Zinchuk O, Okada T (2007). Quantitative colocalization analysis of multicolor confocal immunofluorescence microscopy images: Pushing pixels to explore biological phenomena. Acta Histochem. Cytochem..

[76] Mier-Aguilar CA, Cashman KS, Raman C, Soldevila G (2016). CD5-CK2 signaling modulates Erk activation and thymocyte survival. PLoS ONE.

[77] Janjetovic Z, Tuckey RC, Nguyen MN, Thorpe EM, Slominski AT (2010). 20,23-dihydroxyvitamin D3, novel P450scc product, stimulates differentiation and inhibits proliferation and NF-kappaB activity in human keratinocytes. J. Cell. Physiol..

[78] Axtell RC (2010). T helper type 1 and 17 cells determine efficacy of interferon-beta in multiple sclerosis and experimental encephalomyelitis. Nat. Med..

[79] Rowse AL (2012). Lithium controls central nervous system autoimmunity through modulation of IFN-gamma signaling. PLoS ONE.

[80] Heinz DW, Baase WA, Dahlquist FW, Matthews BW (1993). How amino-acid insertions are allowed in an alpha-helix of T4 lysozyme. Nature.

[81] Case D (2014). The FF14SB force field. Amber.

[82] Case DA (2005). The Amber biomolecular simulation programs. J. Comput. Chem..

[83] Pan D, Song Y (2010). Role of altered sialylation of the I-like domain of beta1 integrin in the binding of fibronectin to beta1 integrin: Thermodynamics and conformational analyses. Biophys. J..

[84] Yan Q, Murphy-Ullrich JE, Song YH (2011). Molecular and structural insight into the role of key residues of thrombospondin-1 and calreticulin in thrombospondin-1-calreticulin binding. Biochemistry.

[85] Pan D, Yan Q, Chen Y, McDonald JM, Song Y (2011). Trifluoperazine regulation of calmodulin binding to Fas: A computational study. Proteins.

[86] Yan Q, Murphy-Ullrich JE, Song YH (2010). Structural insight into the role of thrombospondin-1 binding to calreticulin in calreticulin-induced focal adhesion disassembly. Biochemistry.

[87] Suever JD, Chen Y, McDonald JM, Song Y (2008). Conformation and free energy analyses of the complex of calcium-bound calmodulin and the Fas death domain. Biophys. J..

[88] Liu Y, Pan D, Bellis SL, Song Y (2008). Effect of altered glycosylation on the structure of the I-like domain of beta1 integrin: A molecular dynamics study. Proteins.

[89] Lee SJ, Song Y, Baker NA (2008). Molecular dynamics simulations of asymmetric NaCl and KCl solutions separated by phosphatidylcholine bilayers: Potential drops and structural changes induced by strong Na+–lipid interactions and finite size effects. Biophys. J..

[90] Song Y, Guallar V, Baker NA (2005). Molecular dynamics simulations of salicylate effects on the micro- and mesoscopic properties of a dipalmitoylphosphatidylcholine bilayer. Biochemistry.

[91] Yang H, Song Y (2016). Structural insight for roles of DR5 death domain mutations on oligomerization of DR5 death domain-FADD complex in the death-inducing signaling complex formation: A computational study. J. Mol. Model.

[92] Wang L, Murphy-Ullrich JE, Song Y (2019). Multiscale simulation of the interaction of calreticulin-thrombospondin-1 complex with a model membrane microdomain. J. Biomol. Struct. Dyn..

[93] Wang L, Pan D, Yan Q, Song Y (2017). Activation mechanisms of alphaVbeta3 integrin by binding to fibronectin: A computational study. Protein Sci..

[94] Kollman PA (2000). Calculating structures and free energies of complex molecules: Combining molecular mechanics and continuum models. Acc. Chem. Res..

[95] Wang W (2001). An analysis of the interactions between the Sem-5 SH3 domain and its ligands using molecular dynamics, free energy calculations, and sequence analysis. J. Am. Chem. Soc..

[96] Ganoth A, Friedman R, Nachliel E, Gutman M (2006). A molecular dynamics study and free energy analysis of complexes between the Mlc1p protein and two IQ motif peptides. Biophys. J..

[97] Baker NA, Sept D, Joseph S, Holst MJ, McCammon JA (2001). Electrostatics of nanosystems: Application to microtubules and the ribosome. Proc. Natl. Acad. Sci. U. S. A..

[98] Jurrus E (2018). Improvements to the APBS biomolecular solvation software suite. Protein Sci..

[99] The PyMOL Molecular Graphics System v. 2.4.0 (Schrödinger, LLC URL https://pymol.org/2/).

[100] Microsoft Excel v. 2019 (Microsoft Corporation URL https://office.microsoft.com/excel).

[101] Humphrey W, Dalke A, Schulten K (1996). VMD: Visual molecular dynamics. J. Mol. Graph..

