# Supplementary material for: Vitamin D and lumisterol derivatives can act on liver X receptors (LXRs)
Source: Sci Rep. 2021 Apr 13;11:8002. doi: 10.1038/s41598-021-87061-w (PMC8044163; doi:10.1038/s41598-021-87061-w)
Supplement: Supplementary file 3 — Supplementary Information. [file 41598_2021_87061_MOESM3_ESM.docx]

**Supplemental file for Vitamin D and lumisterol derivatives can act on liver X receptors (LXRs)**

Andrzej T Slominski^1,2,3^*, Tae-Kang Kim ^1^, Shariq Qayyum^1^, Yuwei Song^1^, Zorica Janjetovic^1^, Allen SW Oak^1^, Radomir M Slominski^1^, Chander Raman^1^, Joanna Stefan^1, 4^, Carlos A Mier-Aguilar^1^, Venkatram Atigadda^1,5^, David K Crossman^6^, Andriy Golub^7^, Yaroslav Bilokin^7^, Edith K Y Tang^8^, Jake Y. Chen^9^, Robert C Tuckey^8^, Anton M Jetten^10^, Yuhua Song^11^*

^1^Department of Dermatology, ^2^Comprehensive Cancer Center, Cancer Chemoprevention Program, University of Alabama at Birmingham, ^3^Pathology and Laboratory Medicine Service, VA Medical Center, Birmingham, AL 35249. USA,

^4^Department of Oncology, Nicolaus Copernicus University Medical College, Romanowskiej str. 2, 85-796 Bydgoszcz, Poland,

^5^Department of Chemistry, ^6^Department of Genetics, Genomics Core Facility, University of Alabama at Birmingham, Birmingham, AL 35249. USA,

^7^**OTAVA LTD,** Vaughan, Ontario,  L4K 0C3, Canada,

^8^School of Molecular Sciences, The University of Western Australia, Perth, WA, Australia,

^9^Informatics Institute, University of Alabama at Birmingham, Birmingham, AL 35249. USA,

^10^Cell Biology Section, National Institute of Environmental Health Sciences, National Institutes of Health, Research Triangle Park, NC 27709, USA,

^11^Department of Biomedical Engineering, University of Alabama at Birmingham, Birmingham, AL 35249. USA.

**Running title:** Secosteroids and lumisterols as ligands for LXR

***Equal corresponding authors**: Andrzej T. Slominski, MD, PhD; Department of Dermatology; 1670 University Blvd, Rm 476; Birmingham; Tel: (205) 934-5245; Fax: (205) 996-0302; E-mail: [aslominski@uabmc.edu](mailto:aslominski@uabmc.edu); Yuhua, Song, PhD, Dept. of Biomedical Engineering, Shelby 803 Tel: (205) 996-6939; Fax: (205) 975-4919; E-mail:yhsong@uab.edu

**Supplemental Table 1:** Common up and downregulated protein coding genes by 1,25(OH)_2_D3, 1,20,23(OH)_3_D3, 20,23(OH)_2_D3 and 20(OH)cholesterol with expression level measured in comparison to solvent control (0.1% ethanol) using FC≥2.

| **GENE** | **Control** | **1,25(OH)2D3** | **20,23(OH)2D3** | **1,20,23(OH)3D3** | **20(OH)Chol** | **1,25(OH)2D3 vs control log2(fold_change)** | **20,23(OH)2D3 vs control log2(fold_change)** | **1,20,23(OH)3D3 vs control log2(fold_change)** | **20(OH)Chol vs control log2(fold_change)** |
| --- | --- | --- | --- | --- | --- | --- | --- | --- | --- |
| Cd68 | 1.1418825 | 5.27372 | 3.47805 | 3.54082 | 2.99226 | 2.20524 | 1.60152 | 1.639 | 1.39102 |
| Krt19 | 5.0632125 | 14.9744 | 10.8799 | 10.3867 | 12.2793 | 1.56221 | 1.0982 | 1.04295 | 1.27931 |
| Hist1h2ab | 0.2873505 | 1.14391 | 1.7305 | 1.43046 | 1.14844 | 1.99093 | 2.58496 | 2.32193 | 2 |
| Cox15 | 5.8423 | 15.3542 | 16.947 | 15.6597 | 14.0674 | 1.39186 | 1.53108 | 1.42878 | 1.26895 |
| Ptpa | 66.28305 | 235.847 | 177.146 | 214.561 | 149.828 | 1.82897 | 1.41288 | 1.70101 | 1.1778 |
| Adh7 | 0.2568355 | 1.04202 | 1.15799 | 1.31287 | 0.775405 | 2.0183 | 2.16736 | 2.36014 | 1.59531 |
| Stmn1 | 1.5525525 | 3.69083 | 6.59092 | 5.27199 | 6.2905 | 1.24713 | 2.0805 | 1.77004 | 2.01974 |
| Susd1 | 0.69417475 | 2.15105 | 1.93554 | 2.29644 | 2.15775 | 1.6295 | 1.47402 | 1.73236 | 1.63736 |
| Btc | 0.24243425 | 0.951389 | 0.882886 | 1.11737 | 1.11239 | 1.97027 | 1.8593 | 2.21077 | 2.1992 |
| M1ap | 0.6188625 | 1.71884 | 1.3491 | 2.13957 | 2.48352 | 1.47158 | 1.11897 | 1.79596 | 2.0059 |
| Aqp11 | 0.22359875 | 0.956186 | 0.98281 | 0.939445 | 0.807322 | 2.09421 | 2.13066 | 2.07723 | 1.85344 |
| Nckipsd | 4.169295 | 15.4716 | 11.1997 | 23.2533 | 8.76458 | 1.88958 | 1.42024 | 2.48589 | 1.07309 |
| Cnnm4 | 50.231475 | 11.9457 | 3.14843 | 4.2236 | 3.86435 | -2.07427 | -4.00123 | -3.56572 | -3.69909 |
| Zbtb18 | 44.466675 | 5.62297 | 4.96588 | 4.22881 | 5.28931 | -2.98549 | -3.16794 | -3.38807 | -3.07037 |
| Rnu3a | 2.200155 | 0 | 0 | 0 | 0 | -inf | -inf | -inf | -inf |
| Ccl4 | 0.84806975 | 0 | 0.170243 | 0.337742 | 0.169473 | -inf | -2.32193 | -1.32193 | -2.32193 |
| Fmnl1 | 0.93366725 | 0.0441508 | 0.0887499 | 0.219226 | 0.0588987 | -4.40457 | -3.40043 | -2.08416 | -3.9854 |
| Slc16a5 | 0.92296475 | 0.17311 | 0.232908 | 0.0721514 | 0.45641 | -2.41676 | -1.99185 | -3.67085 | -1.01474 |
| Med9os | 2.0745275 | 0.782595 | 0.796477 | 0.782896 | 0.319092 | -1.40861 | -1.38642 | -1.39956 | -2.69954 |
| Rsad1 | 3.1396475 | 1.44719 | 1.04533 | 1.41866 | 1.41339 | -1.11952 | -1.59199 | -1.13974 | -1.15024 |
| Sgca | 1.23549 | 0.379142 | 0.382376 | 0.568943 | 0.380646 | -1.70644 | -1.69735 | -1.11239 | -1.69735 |
| Erh | 6.2900125 | 2.72768 | 2.14539 | 2.61816 | 1.52032 | -1.20756 | -1.55716 | -1.25818 | -2.04748 |
| Hist1h2bg | 2.26517 | 0.563589 | 0.568392 | 0 | 0.565821 | -2.00907 | -2 | -inf | -2 |
| Hist1h2bh | 0.8389365 | 0 | 0 | 0 | 0 | -inf | -inf | -inf | -inf |
| Hist1h4c | 0.80848325 | 0 | 0 | 0.402471 | 0.403904 | -inf | -inf | -1 | -1 |
| Isl1 | 0.80346925 | 0.224933 | 0.323857 | 0.324631 | 0.342867 | -1.83892 | -1.31622 | -1.30111 | -1.22739 |
| Defb42 | 0.91347575 | 0.230214 | 0.0911204 | 0.220309 | 0.361821 | -1.99056 | -3.33086 | -2.04551 | -1.33489 |
| Rpph1 | 1.31638 | 0.655049 | 0.660632 | 0.655309 | 0.657642 | -1.00907 | -1 | -1 | -1 |
| Parvg | 1.4818275 | 0.159462 | 0.435945 | 0.159525 | 0.0960489 | -3.21826 | -1.7705 | -3.20919 | -3.94626 |
| Scn8a | 0.802881 | 0.36025 | 0.117675 | 0.19979 | 0.297794 | -1.15836 | -2.77572 | -2.00037 | -1.42967 |
| Samd12 | 3.6042975 | 0.74267 | 1.71012 | 0.272829 | 0.547628 | -2.28109 | -1.08096 | -3.71732 | -2.71725 |
| Cd200 | 1.4145525 | 0.399774 | 0.492988 | 0.577686 | 0.445952 | -1.82526 | -1.52606 | -1.28566 | -1.66418 |
| Snhg9 | 18.693175 | 3.72079 | 7.505 | 3.72227 | 7.47104 | -2.331 | -1.32193 | -2.32193 | -1.32193 |
| Cbs | 1.9470425 | 0.41887 | 0.284085 | 0.298895 | 0.533277 | -2.21888 | -2.78223 | -2.69724 | -1.86712 |
| Syt12 | 4.10167 | 0.99028 | 0.990686 | 0.982812 | 1.31831 | -2.05247 | -2.05505 | -2.05489 | -1.63632 |
| Psd4 | 1.568305 | 0.72587 | 0.15251 | 0.101759 | 0.17918 | -1.11359 | -3.36757 | -3.93964 | -3.12852 |
| Tldc2 | 0.8889765 | 0.380996 | 0.192108 | 0 | 0 | -1.22454 | -2.21557 | -inf | -inf |
| Lhx6 | 11.665425 | 3.88347 | 2.27768 | 2.87885 | 2.20275 | -1.58899 | -2.36194 | -2.01234 | -2.40365 |
| Nr6a1 | 1.8459125 | 0.358234 | 0.322996 | 0.206752 | 0.373467 | -2.36753 | -2.52009 | -3.15203 | -2.30408 |
| Ehf | 0.83270725 | 0.283058 | 0.159384 | 0.298467 | 0.317145 | -1.55888 | -2.39065 | -1.4739 | -1.39146 |
| Tnnc2 | 0.8007985 | 0 | 0.313012 | 0.31049 | 0.155779 | -inf | -1.36056 | -1.36056 | -2.36073 |
| Larp1b | 19.6682815 | 2.78749 | 3.39863 | 6.30437 | 3.61308 | -3.23051 | -2.94769 | 5.0789 | -2.85287 |
| Unc5c | 1.08974 | 0.326351 | 0.1434 | 0.456913 | 0.496337 | -1.74165 | -2.93121 | -1.24766 | -1.13338 |
| Dcun1d1 | 13.2195 | 6.45748 | 4.48623 | 4.16178 | 3.93819 | -1.0358 | -1.56443 | -1.66106 | -1.74586 |
| Pde4dip | 3929.8175 | 20.1604 | 25.3974 | 24.1833 | 20.5427 | -7.60897 | -7.27898 | -7.33798 | -7.57849 |
| Car9 | 9.40453 | 3.34101 | 2.12587 | 2.81818 | 1.73793 | -1.49524 | -2.15064 | -1.73226 | -2.43478 |
| Zfp618 | 4.77191 | 1.66773 | 1.58324 | 1.3488 | 2.23584 | -1.51885 | -1.59703 | -1.81656 | -1.09254 |
| Dtx1 | 1.6629925 | 0.251848 | 0.681598 | 0.141978 | 0.617201 | -2.72533 | -1.29213 | -3.54371 | -1.42876 |
| Slc15a4 | 57.122325 | 20.8062 | 15.7859 | 13.3996 | 13.0176 | -1.45921 | -1.86076 | -2.08554 | -2.13238 |
| St7 | 68.998125 | 31.903 | 25.5959 | 24.6198 | 21.7063 | -1.11503 | -1.43598 | -1.48041 | -1.66724 |
| Cpa2 | 1.5708675 | 0.31267 | 0.313747 | 0.312797 | 0.313911 | -2.33102 | -2.32923 | -2.32193 | -2.32193 |
| Clec2g | 1.013685 | 0 | 0 | 0 | 0 | -inf | -inf | -inf | -inf |
| Bicd1 | 2.1379125 | 0.730446 | 0.813225 | 0.705842 | 0.390017 | -1.55152 | -1.39982 | -1.59246 | -2.45339 |
| Lsm5 | 2.6657225 | 0.989629 | 1.07037 | 1.26873 | 0.884577 | -1.43174 | -1.32175 | -1.06482 | -1.59026 |
| Ptpn6 | 11.9914 | 2.53214 | 0.339388 | 3.90274 | 1.72325 | -2.24574 | -5.14826 | -1.61311 | -2.79759 |
| Gys2 | 1.37047 | 0 | 0 | 0 | 0 | -inf | -inf | -inf | -inf |
| Ercc2 | 169.7445 | 13.8618 | 13.0262 | 13.9366 | 15.1049 | -3.61634 | -3.70921 | -3.60008 | -3.48907 |
| Syt8 | 3.193325 | 1.51698 | 0.710941 | 0.514849 | 0.256233 | -1.07603 | -2.1726 | -2.62651 | -3.63833 |
| Saa1 | 0.954759 | 0.19004 | 0 | 0.190116 | 0.190792 | -2.331 | -inf | -2.32193 | -2.32193 |
| Ntrk3 | 1.8598525 | 0 | 0.00781909 | 0.402471 | 0.369575 | -inf | -7.89931 | -2.2019 | -2.33005 |
| Nr3c2 | 1.10119 | 0.155827 | 0.302515 | 0.0667996 | 0.128507 | -2.82322 | -1.86932 | -4.03675 | -3.09794 |
| Wdr17 | 2.24784 | 0.72128 | 1.10642 | 0.236638 | 0.677045 | -1.64208 | -1.02799 | -3.24146 | -1.73001 |
| Cherp | 184.85775 | 35.1454 | 31.4407 | 32.7784 | 31.8463 | -2.39718 | -2.56105 | -2.48927 | -2.53602 |
| Ctrl | 1.0193275 | 0.3422 | 0.115035 | 0.339976 | 0.455708 | -1.57688 | -3.15282 | -1.57778 | -1.16023 |
| Arid3b | 3.4028275 | 1.26791 | 0.218273 | 0.608167 | 0.91077 | -1.42645 | -3.96787 | -2.47786 | -1.90037 |

**Supplemental Table 2**. Ingenuity canonical pathways activated by 1,25(OH)_2_D3 in murine fibroblasts, nuclear receptors are in bold

| **Ingenuity Canonical Pathways** | **p value** | **Ratio** | **Downregulated** | **Upregulated** | **No overlap with dataset** |
| --- | --- | --- | --- | --- | --- |
| Granulocyte Adhesion and Diapedesis | 5.7544E-09 | 0.11 | 44/181 (24%) | 35/181 (19%) | 102/181 (56%) |
| Agranulocyte Adhesion and Diapedesis | 8.9125E-08 | 0.0984 | 47/193 (24%) | 34/193 (18%) | 112/193 (58%) |
| Hepatic Fibrosis / Hepatic Stellate Cell Activation | 9.5499E-05 | 0.0749 | 73/187 (39%) | 43/187 (23%) | 71/187 (38%) |
| **VDR/RXR Activation** | 0.00038019 | 0.103 | 27/78 (35%) | 26/78 (33%) | 25/78 (32%) |
| Airway Pathology in Chronic Obstructive Pulmonary Disease | 0.00058884 | 0.375 | 3/8 (38%) | 1/8 (13%) | 4/8 (50%) |
| Atherosclerosis Signaling | 0.00245471 | 0.0709 | 29/127 (23%) | 27/127 (21%) | 71/127 (56%) |
| Leukotriene Biosynthesis | 0.00275423 | 0.231 | 3/13 (23%) | 3/13 (23%) | 7/13 (54%) |
| Leukocyte Extravasation Signaling | 0.00323594 | 0.0569 | 52/211 (25%) | 75/211 (36%) | 84/211 (40%) |
| GP6 Signaling Pathway | 0.00346737 | 0.0672 | 45/134 (34%) | 36/134 (27%) | 53/134 (40%) |
| Axonal Guidance Signaling | 0.0040738 | 0.0438 | 146/457 (32%) | 169/457 (37%) | 142/457 (31%) |
| cAMP-mediated signaling | 0.0060256 | 0.0526 | 61/228 (27%) | 50/228 (22%) | 117/228 (51%) |
| Gαi Signaling | 0.00691831 | 0.065 | 38/123 (31%) | 33/123 (27%) | 52/123 (42%) |
| Relaxin Signaling | 0.01 | 0.057 | 57/158 (36%) | 51/158 (32%) | 50/158 (32%) |
| Inhibition of Matrix Metalloproteases | 0.01148154 | 0.103 | 18/39 (46%) | 10/39 (26%) | 11/39 (28%) |
| SPINK1 Pancreatic Cancer Pathway | 0.01148154 | 0.0833 | 12/60 (20%) | 8/60 (13%) | 40/60 (67%) |
| G-Protein Coupled Receptor Signaling | 0.01230269 | 0.0461 | 80/282 (28%) | 80/282 (28%) | 122/282 (43%) |
| Role of Hypercytokinemia/hyperchemokinemia in the Pathogenesis of Influenza | 0.01584893 | 0.093 | 4/43 (9%) | 3/43 (7%) | 36/43 (84%) |
| Pathogenesis of Multiple Sclerosis | 0.01659587 | 0.222 | 3/9 (33%) | 0/9 (0%) | 6/9 (67%) |
| Eicosanoid Signaling | 0.01778279 | 0.0746 | 14/67 (21%) | 14/67 (21%) | 39/67 (58%) |
| Acute Phase Response Signaling | 0.01905461 | 0.0511 | 63/176 (36%) | 53/176 (30%) | 60/176 (34%) |
| Neuroprotective Role of THOP1 in Alzheimer's Disease | 0.01949845 | 0.0583 | 23/120 (19%) | 20/120 (17%) | 77/120 (64%) |
| Bladder Cancer Signaling | 0.01995262 | 0.0638 | 35/94 (37%) | 30/94 (32%) | 29/94 (31%) |
| Role of IL-17F in Allergic Inflammatory Airway Diseases | 0.01995262 | 0.087 | 16/46 (35%) | 17/46 (37%) | 13/46 (28%) |
| **LXR/RXR Activation** | 0.02041738 | 0.0579 | 37/121 (31%) | 24/121 (20%) | 60/121 (50%) |

**Supplemental Table 3**. Ingenuity toxicity pathways activated by 1,25(OH)_2_D3 in murine fibroblasts, nuclear receptors are in bold

| **Ingenuity Toxicity Lists** | **p value** | **Ratio** |
| --- | --- | --- |
| Hepatic Fibrosis | 4.57088E-06 | 0.114 |
| **VDR/RXR Activation** | 0.000380189 | 0.103 |
| Cardiac Fibrosis | 0.004168694 | 0.055 |
| Positive Acute Phase Response Proteins | 0.004466836 | 0.133 |
| Cytochrome P450 Panel - Substrate is a Vitamin (human) | 0.00724436 | 0.333 |
| Renal Necrosis/Cell Death | 0.007413102 | 0.04 |
| Cytochrome P450 Panel - Substrate is a Vitamin (Mouse and Rat) | 0.01 | 0.286 |
| Acute Renal Failure Panel (Rat) | 0.013182567 | 0.0806 |
| Renal Ischemic Resistance Panel (Rat) | 0.020417379 | 0.2 |
| Increases Renal Damage | 0.020892961 | 0.0632 |
| **LXR/RXR Activation** | 0.021877616 | 0.0569 |

**Supplemental Table 4**. Ingenuity canonical pathways activated by 20,23(OH)_2_D3 in murine fibroblasts, nuclear receptors are in bold

| **Ingenuity Canonical Pathways** | **p value** | **Ratio** | **Downregulated** | **Upregulated** | **No overlap with dataset** |
| --- | --- | --- | --- | --- | --- |
| Estrogen-mediated S-phase Entry | 0.000707946 | 0.154 | 13/26 (50%) | 10/26 (38%) | 3/26 (12%) |
| Atherosclerosis Signaling | 0.000954993 | 0.063 | 33/127 (26%) | 25/127 (20%) | 69/127 (54%) |
| Cell Cycle Regulation by BTG Family Proteins | 0.002691535 | 0.108 | 14/37 (38%) | 17/37 (46%) | 6/37 (16%) |
| EIF2 Signaling | 0.003467369 | 0.0441 | 81/227 (36%) | 102/227 (45%) | 43/227 (19%) |
| Communication between Innate and Adaptive Immune Cells | 0.004265795 | 0.0625 | 7/96 (7%) | 16/96 (17%) | 73/96 (76%) |
| Role of Hypercytokinemia/hyperchemokinemia in the Pathogenesis of Influenza | 0.004677351 | 0.093 | 2/43 (5%) | 5/43 (12%) | 36/43 (84%) |
| Cyclins and Cell Cycle Regulation | 0.009332543 | 0.0617 | 42/81 (52%) | 28/81 (35%) | 11/81 (14%) |
| Role of Cytokines in Mediating Communication between Immune Cells | 0.010471285 | 0.0741 | 3/54 (6%) | 5/54 (9%) | 46/54 (85%) |
| Transcriptional Regulatory Network in Embryonic Stem Cells | 0.010471285 | 0.0741 | 12/54 (22%) | 8/54 (15%) | 33/54 (61%) |
| **LXR/RXR Activation** | 0.012589254 | 0.0496 | 37/121 (31%) | 23/121 (19%) | 61/121 (50%) |
| Altered T Cell and B Cell Signaling in Rheumatoid Arthritis | 0.014125375 | 0.0556 | 14/90 (16%) | 15/90 (17%) | 61/90 (68%) |
| Hematopoiesis from Multipotent Stem Cells | 0.014791084 | 0.167 | 2/12 (17%) | 7/12 (58%) | 3/12 (25%) |
| DNA Methylation and Transcriptional Repression Signaling | 0.016218101 | 0.0882 | 12/34 (35%) | 12/34 (35%) | 9/34 (26%) |
| Adipogenesis pathway | 0.019952623 | 0.0448 | 49/134 (37%) | 60/134 (45%) | 25/134 (19%) |
| Cell Cycle: G1/S Checkpoint Regulation | 0.021877616 | 0.0597 | 34/67 (51%) | 28/67 (42%) | 5/67 (7%) |
| Eicosanoid Signaling | 0.021877616 | 0.0597 | 13/67 (19%) | 16/67 (24%) | 38/67 (57%) |
| nNOS Signaling in Skeletal Muscle Cells | 0.026915348 | 0.0732 | 7/41 (17%) | 10/41 (24%) | 24/41 (59%) |
| Cysteine Biosynthesis/Homocysteine Degradation | 0.031622777 | 0.5 | 2/2 (100%) | 0/2 (0%) | 0/2 (0%) |
| Systemic Lupus Erythematosus Signaling | 0.033884416 | 0.0342 | 54/234 (23%) | 63/234 (27%) | 117/234 (50%) |
| GADD45 Signaling | 0.035481339 | 0.105 | 13/19 (68%) | 6/19 (32%) | 0/19 (0%) |
| Inflammasome pathway | 0.039810717 | 0.1 | 7/20 (35%) | 5/20 (25%) | 8/20 (40%) |
| Graft-versus-Host Disease Signaling | 0.040738028 | 0.0625 | 5/48 (10%) | 8/48 (17%) | 35/48 (73%) |
| mTOR Signaling | 0.046773514 | 0.034 | 77/206 (37%) | 87/206 (42%) | 41/206 (20%) |
| Glutamate Degradation II | 0.046773514 | 0.333 | 0/3 (0%) | 3/3 (100%) | 0/3 (0%) |
| Aspartate Biosynthesis | 0.046773514 | 0.333 | 0/3 (0%) | 3/3 (100%) | 0/3 (0%) |
| Allograft Rejection Signaling | 0.047863009 | 0.0465 | 7/86 (8%) | 10/86 (12%) | 69/86 (80%) |
| **FXR/RXR Activation** | 0.050118723 | 0.0397 | 18/126 (14%) | 23/126 (18%) | 85/126 (67%) |

**Supplemental Table 5**. Ingenuity toxicity pathways activated by 20,23(OH)_2_D3 in murine fibroblasts, nuclear receptors are in bold

| **Ingenuity Toxicity Lists** | **p value** | **Ratio** |
| --- | --- | --- |
| **LXR/RXR Activation** | 0.013804 | 0.0488 |
| Renal Necrosis/Cell Death | 0.015136 | 0.0291 |
| Liver Proliferation | 0.016218 | 0.0369 |
| Cell Cycle: G1/S Checkpoint Regulation | 0.023988 | 0.058 |
| **FXR/RXR Activation** | 0.050119 | 0.0397 |
| Increases Renal Damage | 0.064565 | 0.0421 |
| Acute Renal Failure Panel (Rat) | 0.075858 | 0.0484 |
| Positive Acute Phase Response Proteins | 0.081283 | 0.0667 |
| Hepatic Fibrosis | 0.085114 | 0.0381 |
| Hepatic Cholestasis | 0.117761 | 0.0307 |
| Vasopressin-induced Genes in Inner Medullary Renal Collecting Duct Cells (Rat) | 0.11995 | 0.125 |
| Cardiac Hypertrophy | 0.121619 | 0.0249 |
| Liver Necrosis/Cell Death | 0.124451 | 0.0256 |
| Increases Glomerular Injury | 0.152405 | 0.0353 |
| Increases Damage of Mitochondria | 0.161065 | 0.0909 |
| Increases Transmembrane Potential of Mitochondria and Mitochondrial Membrane | 0.187499 | 0.04 |
| Mechanism of Gene Regulation by Peroxisome Proliferators via PPARα | 0.190985 | 0.0316 |
| Cardiac Necrosis/Cell Death | 0.193642 | 0.0236 |
| Increases Liver Steatosis | 0.228034 | 0.0288 |
| Long-term Renal Injury Anti-oxidative Response Panel (Rat) | 0.250035 | 0.0556 |
| **Aryl Hydrocarbon Receptor Signaling** | 0.252348 | 0.0248 |

**Supplemental Table 6**. Ingenuity canonical pathways activated by 1,20,23(OH)_3_D3 in murine fibroblasts, nuclear receptors are in bold

| **Ingenuity Canonical Pathways** | **p value** | **Ratio** | **Downregulated** | **Upregulated** | **No overlap with dataset** |
| --- | --- | --- | --- | --- | --- |
| GP6 Signaling Pathway | 0.000427 | 0.0672 | 28/134 (21%) | 58/134 (43%) | 48/134 (36%) |
| NF-κB Signaling | 0.004365 | 0.0481 | 55/187 (29%) | 75/187 (40%) | 57/187 (30%) |
| Hepatic Cholestasis | 0.005623 | 0.05 | 36/160 (23%) | 51/160 (32%) | 73/160 (46%) |
| Sphingomyelin Metabolism | 0.007244 | 0.25 | 3/8 (38%) | 3/8 (38%) | 2/8 (25%) |
| p70S6K Signaling | 0.00871 | 0.0507 | 28/138 (20%) | 73/138 (53%) | 37/138 (27%) |
| Phototransduction Pathway | 0.011749 | 0.0755 | 6/53 (11%) | 12/53 (23%) | 35/53 (66%) |
| Apelin Cardiomyocyte Signaling Pathway | 0.012023 | 0.0531 | 25/113 (22%) | 50/113 (44%) | 38/113 (34%) |
| GDNF Family Ligand-Receptor Interactions | 0.012023 | 0.061 | 17/82 (21%) | 47/82 (57%) | 18/82 (22%) |
| Growth Hormone Signaling | 0.01349 | 0.0595 | 15/84 (18%) | 44/84 (52%) | 25/84 (30%) |
| EIF2 Signaling | 0.014454 | 0.0396 | 94/227 (41%) | 88/227 (39%) | 44/227 (19%) |
| p38 MAPK Signaling | 0.015136 | 0.0504 | 36/119 (30%) | 44/119 (37%) | 39/119 (33%) |
| **LXR/RXR Activation** | 0.016218 | 0.0496 | 23/121 (19%) | 37/121 (31%) | 61/121 (50%) |
| Production of Nitric Oxide and Reactive Oxygen Species in Macrophages | 0.016596 | 0.0412 | 44/194 (23%) | 82/194 (42%) | 68/194 (35%) |
| Role of Osteoblasts, Osteoclasts and Chondrocytes in Rheumatoid Arthritis | 0.016982 | 0.0386 | 55/233 (24%) | 94/233 (40%) | 84/233 (36%) |
| Atherosclerosis Signaling | 0.019953 | 0.0472 | 25/127 (20%) | 30/127 (24%) | 72/127 (57%) |
| Natural Killer Cell Signaling | 0.020893 | 0.0469 | 25/128 (20%) | 48/128 (38%) | 55/128 (43%) |
| IL-4 Signaling | 0.020893 | 0.0532 | 24/94 (26%) | 42/94 (45%) | 28/94 (30%) |
| Ceramide Signaling | 0.025119 | 0.0505 | 26/99 (26%) | 51/99 (52%) | 22/99 (22%) |
| IL-6 Signaling | 0.025119 | 0.0448 | 35/134 (26%) | 68/134 (51%) | 31/134 (23%) |
| Role of Pattern Recognition Receptors in Recognition of Bacteria and Viruses | 0.027542 | 0.0438 | 34/137 (25%) | 40/137 (29%) | 63/137 (46%) |
| **ErbB Signaling** | 0.0302 | 0.0481 | 22/104 (21%) | 57/104 (55%) | 25/104 (24%) |
| CREB Signaling in Neurons | 0.030903 | 0.0367 | 52/218 (24%) | 86/218 (39%) | 79/218 (36%) |
| MSP-RON Signaling Pathway | 0.032359 | 0.0556 | 11/72 (15%) | 22/72 (31%) | 38/72 (53%) |

**Supplemental Table 7**. Ingenuity toxicity pathways activated by 1,20,23(OH)_3_D3 in murine fibroblasts, nuclear receptors are in bold

| **Ingenuity Toxicity Lists** | **p value** | **Ratio** |
| --- | --- | --- |
| Increases Renal Damage | 0.005248 | 0.0632 |
| Renal Necrosis/Cell Death | 0.005495 | 0.0327 |
| Hepatic Cholestasis | 0.00631 | 0.0491 |
| Cardiac Fibrosis | 0.011482 | 0.0413 |
| **LXR/RXR Activation** | 0.017378 | 0.0488 |
| NF-κB Signaling | 0.018197 | 0.0362 |
| Liver Proliferation | 0.053703 | 0.0328 |
| Increases Glomerular Injury | 0.053703 | 0.0471 |
| Cardiac Hypertrophy | 0.083176 | 0.0277 |
| **CAR/RXR Activation** | 0.085114 | 0.069 |
| Hepatic Fibrosis | 0.1 | 0.0381 |
| LPS/IL-1 Mediated Inhibition of RXR Function | 0.136773 | 0.0275 |
| **FXR/RXR Activation** | 0.160694 | 0.0317 |
| Increases Liver Hyperplasia/Hyperproliferation | 0.170216 | 0.031 |
| Glutathione Depletion - CYP Induction and Reactive Metabolites | 0.183231 | 0.0833 |
| Decreases Respiration of Mitochondria | 0.196789 | 0.0769 |
| Cytochrome P450 Panel - Substrate is a Sterol (Mouse) | 0.210378 | 0.0714 |
| Mechanism of Gene Regulation by Peroxisome Proliferators via PPARα | 0.212324 | 0.0316 |
| **RAR Activation** | 0.213304 | 0.0263 |
| Cell Cycle: G2/M DNA Damage Checkpoint Regulation | 0.22182 | 0.0377 |
| **THR/RXR Activation** | 0.225424 | 0.0306 |
| Cardiac Necrosis/Cell Death | 0.230144 | 0.0236 |
| Increases Renal Proliferation | 0.235505 | 0.027 |
| Xenobiotic Metabolism Signaling | 0.260615 | 0.0221 |
| Increases Bradycardia | 0.286418 | 0.05 |
| Biogenesis of Mitochondria | 0.286418 | 0.05 |
| Glutathione Depletion - Phase II Reactions | 0.286418 | 0.05 |
| Cell Cycle: G1/S Checkpoint Regulation | 0.320627 | 0.029 |
| Increases Liver Damage | 0.347536 | 0.024 |
| Decreases Transmembrane Potential of Mitochondria and Mitochondrial Membrane | 0.352371 | 0.0238 |
| Increases Liver Hepatitis | 0.363915 | 0.0263 |
| Reversible Glomerulonephritis Biomarker Panel (Rat) | 0.365595 | 0.037 |
| **VDR/RXR Activation** | 0.375837 | 0.0256 |

**Supplemental Table 8**. Ingenuity canonical pathways activated by 20(OH)C in murine fibroblasts, nuclear receptors are in bold

| **Ingenuity Canonical Pathways** | p value | Ratio | Downregulated | Upregulated | No overlap with dataset |
| --- | --- | --- | --- | --- | --- |
| Protein Kinase A Signaling | 8.32E-05 | 0.0448 | 144/402 (36%) | 128/402 (32%) | 128/402 (32%) |
| **RAR Activation** | 0.000933 | 0.0526 | 58/190 (31%) | 89/190 (47%) | 42/190 (22%) |
| Axonal Guidance Signaling | 0.001072 | 0.0372 | 157/457 (34%) | 159/457 (35%) | 140/457 (31%) |
| Osteoarthritis Pathway | 0.002138 | 0.0472 | 68/212 (32%) | 73/212 (34%) | 71/212 (33%) |
| NF-κB Signaling | 0.00309 | 0.0481 | 56/187 (30%) | 79/187 (42%) | 52/187 (28%) |
| Type II Diabetes Mellitus Signaling | 0.003236 | 0.0519 | 38/154 (25%) | 60/154 (39%) | 56/154 (36%) |
| Agranulocyte Adhesion and Diapedesis | 0.003802 | 0.0466 | 40/193 (21%) | 43/193 (22%) | 110/193 (57%) |
| Bladder Cancer Signaling | 0.003802 | 0.0638 | 26/94 (28%) | 40/94 (43%) | 27/94 (29%) |
| Hepatic Cholestasis | 0.004074 | 0.05 | 36/160 (23%) | 52/160 (33%) | 71/160 (44%) |
| Granulocyte Adhesion and Diapedesis | 0.008511 | 0.0442 | 38/181 (21%) | 43/181 (24%) | 100/181 (55%) |
| LPS/IL-1 Mediated Inhibition of RXR Function | 0.00955 | 0.0404 | 58/223 (26%) | 63/223 (28%) | 102/223 (46%) |
| Molecular Mechanisms of Cancer | 0.010715 | 0.033 | 149/394 (38%) | 152/394 (39%) | 91/394 (23%) |
| Erythropoietin Signaling | 0.012303 | 0.0575 | 34/87 (39%) | 39/87 (45%) | 14/87 (16%) |
| Factors Promoting Cardiogenesis in Vertebrates | 0.015488 | 0.0543 | 34/92 (37%) | 25/92 (27%) | 33/92 (36%) |
| IL-1 Signaling | 0.015488 | 0.0543 | 33/92 (36%) | 41/92 (45%) | 17/92 (18%) |
| Neuregulin Signaling | 0.016218 | 0.0538 | 39/93 (42%) | 41/93 (44%) | 13/93 (14%) |
| Calcium Signaling | 0.017378 | 0.0388 | 59/206 (29%) | 49/206 (24%) | 98/206 (48%) |
| Leukocyte Extravasation Signaling | 0.019498 | 0.0379 | 70/211 (33%) | 60/211 (28%) | 81/211 (38%) |
| Netrin Signaling | 0.019498 | 0.0615 | 22/65 (34%) | 17/65 (26%) | 26/65 (40%) |
| Ceramide Signaling | 0.020893 | 0.0505 | 37/99 (37%) | 41/99 (41%) | 21/99 (21%) |
| Acute Phase Response Signaling | 0.022387 | 0.0398 | 46/176 (26%) | 71/176 (40%) | 59/176 (34%) |
| Corticotropin Releasing Hormone Signaling | 0.023442 | 0.0432 | 34/139 (24%) | 47/139 (34%) | 57/139 (41%) |
| Inhibition of Matrix Metalloproteases | 0.023442 | 0.0769 | 14/39 (36%) | 15/39 (38%) | 10/39 (26%) |
| STAT3 Pathway | 0.023988 | 0.0485 | 38/103 (37%) | 43/103 (42%) | 22/103 (21%) |
| Gα12/13 Signaling | 0.025119 | 0.0426 | 51/141 (36%) | 47/141 (33%) | 43/141 (30%) |
| ErbB Signaling | 0.025119 | 0.0481 | 38/104 (37%) | 42/104 (40%) | 23/104 (22%) |
| Cholecystokinin/Gastrin-mediated Signaling | 0.027542 | 0.0467 | 34/107 (32%) | 47/107 (44%) | 26/107 (24%) |
| tRNA Splicing | 0.02884 | 0.0714 | 18/42 (43%) | 13/42 (31%) | 11/42 (26%) |
| Role of Hypercytokinemia/hyperchemokinemia in the Pathogenesis of Influenza | 0.0302 | 0.0698 | 1/43 (2%) | 7/43 (16%) | 35/43 (81%) |
| All-trans-decaprenyl Diphosphate Biosynthesis | 0.031623 | 0.5 | 1/2 (50%) | 1/2 (50%) | 0/2 (0%) |
| Cysteine Biosynthesis/Homocysteine Degradation | 0.031623 | 0.5 | 2/2 (100%) | 0/2 (0%) | 0/2 (0%) |
| Toll-like Receptor Signaling | 0.032359 | 0.0526 | 23/76 (30%) | 29/76 (38%) | 24/76 (32%) |
| Chemokine Signaling | 0.033884 | 0.0519 | 33/77 (43%) | 24/77 (31%) | 20/77 (26%) |
| Production of Nitric Oxide and Reactive Oxygen Species in Macrophages | 0.035481 | 0.0361 | 60/194 (31%) | 70/194 (36%) | 63/194 (32%) |
| Cardiomyocyte Differentiation via BMP Receptors | 0.039811 | 0.1 | 5/20 (25%) | 5/20 (25%) | 10/20 (50%) |
| Relaxin Signaling | 0.040738 | 0.038 | 52/158 (33%) | 54/158 (34%) | 51/158 (32%) |
| Graft-versus-Host Disease Signaling | 0.040738 | 0.0625 | 2/48 (4%) | 11/48 (23%) | 35/48 (73%) |
| p38 MAPK Signaling | 0.040738 | 0.042 | 38/119 (32%) | 42/119 (35%) | 39/119 (33%) |
| UVC-Induced MAPK Signaling | 0.042658 | 0.0612 | 23/49 (47%) | 21/49 (43%) | 5/49 (10%) |
| TNFR1 Signaling | 0.044668 | 0.06 | 17/50 (34%) | 25/50 (50%) | 8/50 (16%) |
| Proline Degradation | 0.046774 | 0.333 | 1/3 (33%) | 1/3 (33%) | 1/3 (33%) |
| 1,25-dihydroxyvitamin D3 Biosynthesis | 0.046774 | 0.333 | 1/3 (33%) | 1/3 (33%) | 1/3 (33%) |
| L-serine Degradation | 0.046774 | 0.333 | 2/3 (67%) | 1/3 (33%) | 0/3 (0%) |
| Dopamine-DARPP32 Feedback in cAMP Signaling | 0.046774 | 0.0366 | 50/164 (30%) | 47/164 (29%) | 66/164 (40%) |
| Apelin Cardiac Fibroblast Signaling Pathway | 0.046774 | 0.0909 | 9/22 (41%) | 8/22 (36%) | 5/22 (23%) |
| Xenobiotic Metabolism Signaling | 0.047863 | 0.0303 | 79/297 (27%) | 102/297 (34%) | 115/297 (39%) |
| Signaling by Rho Family GTPases | 0.047863 | 0.0317 | 91/252 (36%) | 88/252 (35%) | 73/252 (29%) |
| HIPPO signaling | 0.048978 | 0.046 | 40/87 (46%) | 34/87 (39%) | 13/87 (15%) |
| Macropinocytosis Signaling | 0.048978 | 0.046 | 39/87 (45%) | 28/87 (32%) | 20/87 (23%) |
| Thrombin Signaling | 0.050119 | 0.0333 | 72/210 (34%) | 77/210 (37%) | 60/210 (29%) |
| Differential Regulation of Cytokine Production in Intestinal Epithelial Cells by IL-17A and IL-17F | 0.051286 | 0.087 | 1/23 (4%) | 5/23 (22%) | 17/23 (74%) |
| Pyrimidine Deoxyribonucleotides De Novo Biosynthesis I | 0.051286 | 0.087 | 7/23 (30%) | 8/23 (35%) | 8/23 (35%) |
| Breast Cancer Regulation by Stathmin1 | 0.051286 | 0.0332 | 76/211 (36%) | 80/211 (38%) | 54/211 (26%) |
| Sperm Motility | 0.051286 | 0.0394 | 28/127 (22%) | 37/127 (29%) | 62/127 (49%) |
| Transcriptional Regulatory Network in Embryonic Stem Cells | 0.053703 | 0.0556 | 13/54 (24%) | 7/54 (13%) | 34/54 (63%) |
| FGF Signaling | 0.056234 | 0.044 | 30/91 (33%) | 32/91 (35%) | 28/91 (31%) |
| NF-κB Activation by Viruses | 0.060256 | 0.043 | 31/93 (33%) | 42/93 (45%) | 20/93 (22%) |
| Spermine and Spermidine Degradation I | 0.06166 | 0.25 | 1/4 (25%) | 3/4 (75%) | 0/4 (0%) |
| HER-2 Signaling in Breast Cancer | 0.063096 | 0.0426 | 33/94 (35%) | 36/94 (38%) | 24/94 (26%) |
| RhoGDI Signaling | 0.063096 | 0.0339 | 66/177 (37%) | 62/177 (35%) | 49/177 (28%) |
| CCR5 Signaling in Macrophages | 0.064565 | 0.0421 | 27/95 (28%) | 24/95 (25%) | 44/95 (46%) |
| Communication between Innate and Adaptive Immune Cells | 0.066069 | 0.0417 | 9/96 (9%) | 14/96 (15%) | 73/96 (76%) |
| Androgen Signaling | 0.067608 | 0.0365 | 48/137 (35%) | 52/137 (38%) | 37/137 (27%) |
| Synaptic Long Term Depression | 0.067608 | 0.0333 | 47/180 (26%) | 47/180 (26%) | 86/180 (48%) |
| Role of NFAT in Cardiac Hypertrophy | 0.067608 | 0.0311 | 73/225 (32%) | 82/225 (36%) | 69/225 (31%) |
| SPINK1 Pancreatic Cancer Pathway | 0.069183 | 0.05 | 13/60 (22%) | 6/60 (10%) | 41/60 (68%) |
| EIF2 Signaling | 0.070795 | 0.0308 | 89/227 (39%) | 93/227 (41%) | 44/227 (19%) |
| CTLA4 Signaling in Cytotoxic T Lymphocytes | 0.072444 | 0.0404 | 27/99 (27%) | 31/99 (31%) | 41/99 (41%) |
| Cardiac β-adrenergic Signaling | 0.074131 | 0.0355 | 47/141 (33%) | 40/141 (28%) | 53/141 (38%) |
| Trehalose Degradation II (Trehalase) | 0.077625 | 0.2 | 0/5 (0%) | 3/5 (60%) | 2/5 (40%) |
| Role of Osteoblasts, Osteoclasts and Chondrocytes in Rheumatoid Arthritis | 0.077625 | 0.03 | 73/233 (31%) | 75/233 (32%) | 84/233 (36%) |
| Systemic Lupus Erythematosus Signaling | 0.079433 | 0.0299 | 60/234 (26%) | 57/234 (24%) | 117/234 (50%) |
| TNFR2 Signaling | 0.081283 | 0.0667 | 8/30 (27%) | 17/30 (57%) | 5/30 (17%) |
| Sonic Hedgehog Signaling | 0.081283 | 0.0667 | 9/30 (30%) | 14/30 (47%) | 7/30 (23%) |
| Calcium-induced T Lymphocyte Apoptosis | 0.087096 | 0.0455 | 19/66 (29%) | 14/66 (21%) | 33/66 (50%) |
| Cell Cycle: G1/S Checkpoint Regulation | 0.091201 | 0.0448 | 31/67 (46%) | 30/67 (45%) | 5/67 (7%) |
| Fatty Acid β-oxidation I | 0.091201 | 0.0625 | 15/32 (47%) | 12/32 (38%) | 5/32 (16%) |
| Glycogen Biosynthesis II (from UDP-D-Glucose) | 0.091201 | 0.167 | 3/6 (50%) | 2/6 (33%) | 1/6 (17%) |
| Rapoport-Luebering Glycolytic Shunt | 0.091201 | 0.167 | 3/6 (50%) | 2/6 (33%) | 1/6 (17%) |
| GDP-mannose Biosynthesis | 0.091201 | 0.167 | 2/6 (33%) | 4/6 (67%) | 0/6 (0%) |
| Phospholipase C Signaling | 0.095499 | 0.0286 | 72/245 (29%) | 83/245 (34%) | 89/245 (36%) |
| IL-10 Signaling | 0.095499 | 0.0435 | 17/69 (25%) | 31/69 (45%) | 21/69 (30%) |
| NRF2-mediated Oxidative Stress Response | 0.1 | 0.03 | 74/200 (37%) | 88/200 (44%) | 38/200 (19%) |
| **Glucocorticoid Receptor Signaling** | 0.1 | 0.0261 | 108/345 (31%) | 120/345 (35%) | 117/345 (34%) |
| DNA Methylation and Transcriptional Repression Signaling | 0.100925 | 0.0588 | 9/34 (26%) | 15/34 (44%) | 10/34 (29%) |
| Caveolar-mediated Endocytosis Signaling | 0.103039 | 0.0423 | 28/71 (39%) | 24/71 (34%) | 19/71 (27%) |
| ERK5 Signaling | 0.103039 | 0.0423 | 29/71 (41%) | 36/71 (51%) | 6/71 (8%) |
| Nitric Oxide Signaling in the Cardiovascular System | 0.104954 | 0.0354 | 33/113 (29%) | 38/113 (34%) | 42/113 (37%) |
| Apelin Cardiomyocyte Signaling Pathway | 0.104954 | 0.0354 | 42/113 (37%) | 33/113 (29%) | 38/113 (34%) |
| Melatonin Signaling | 0.105925 | 0.0417 | 21/72 (29%) | 27/72 (38%) | 24/72 (33%) |
| Basal Cell Carcinoma Signaling | 0.105925 | 0.0417 | 16/72 (22%) | 22/72 (31%) | 34/72 (47%) |
| MIF-mediated Glucocorticoid Regulation | 0.105925 | 0.0571 | 9/35 (26%) | 11/35 (31%) | 15/35 (43%) |
| Ceramide Biosynthesis | 0.105925 | 0.143 | 2/7 (29%) | 3/7 (43%) | 2/7 (29%) |
| Ephrin B Signaling | 0.109901 | 0.0411 | 35/73 (48%) | 23/73 (32%) | 15/73 (21%) |
| Virus Entry via Endocytic Pathways | 0.11298 | 0.0345 | 47/116 (41%) | 40/116 (34%) | 29/116 (25%) |
| Ethanol Degradation II | 0.115878 | 0.0541 | 9/37 (24%) | 13/37 (35%) | 15/37 (41%) |
| Airway Pathology in Chronic Obstructive Pulmonary Disease | 0.11995 | 0.125 | 1/8 (13%) | 3/8 (38%) | 4/8 (50%) |
| Sphingomyelin Metabolism | 0.11995 | 0.125 | 3/8 (38%) | 3/8 (38%) | 2/8 (25%) |
| Antigen Presentation Pathway | 0.12106 | 0.0526 | 11/38 (29%) | 10/38 (26%) | 17/38 (45%) |
| Notch Signaling | 0.12106 | 0.0526 | 11/38 (29%) | 12/38 (32%) | 15/38 (39%) |
| PKCθ Signaling in T Lymphocytes | 0.121899 | 0.0303 | 43/165 (26%) | 54/165 (33%) | 67/165 (41%) |
| G Beta Gamma Signaling | 0.123027 | 0.0333 | 41/120 (34%) | 41/120 (34%) | 38/120 (32%) |
| Dopamine Receptor Signaling | 0.123027 | 0.039 | 21/77 (27%) | 27/77 (35%) | 28/77 (36%) |
| **VDR/RXR Activation** | 0.127057 | 0.0385 | 23/78 (29%) | 28/78 (36%) | 26/78 (33%) |
| Tight Junction Signaling | 0.127057 | 0.0299 | 66/167 (40%) | 46/167 (28%) | 55/167 (33%) |
| April Mediated Signaling | 0.127057 | 0.0513 | 10/39 (26%) | 23/39 (59%) | 6/39 (15%) |
| ErbB4 Signaling | 0.127057 | 0.0385 | 32/78 (41%) | 29/78 (37%) | 17/78 (22%) |
| Noradrenaline and Adrenaline Degradation | 0.13213 | 0.05 | 11/40 (28%) | 15/40 (38%) | 14/40 (35%) |
| Role of BRCA1 in DNA Damage Response | 0.133968 | 0.0375 | 35/80 (44%) | 39/80 (49%) | 6/80 (8%) |
| Pathogenesis of Multiple Sclerosis | 0.133968 | 0.111 | 1/9 (11%) | 2/9 (22%) | 6/9 (67%) |
| Citrulline Biosynthesis | 0.133968 | 0.111 | 5/9 (56%) | 0/9 (0%) | 4/9 (44%) |
| CXCR4 Signaling | 0.136144 | 0.0292 | 64/171 (37%) | 60/171 (35%) | 46/171 (27%) |
| GNRH Signaling | 0.136144 | 0.0292 | 49/171 (29%) | 70/171 (41%) | 50/171 (29%) |
| B Cell Activating Factor Signaling | 0.137088 | 0.0488 | 11/41 (27%) | 24/41 (59%) | 6/41 (15%) |
| Mechanisms of Viral Exit from Host Cells | 0.137088 | 0.0488 | 15/41 (37%) | 16/41 (39%) | 10/41 (24%) |
| Cyclins and Cell Cycle Regulation | 0.137088 | 0.037 | 38/81 (47%) | 32/81 (40%) | 11/81 (14%) |
| Apelin Endothelial Signaling Pathway | 0.137088 | 0.032 | 44/125 (35%) | 48/125 (38%) | 32/125 (26%) |
| Role of Macrophages, Fibroblasts and Endothelial Cells in Rheumatoid Arthritis | 0.138995 | 0.0249 | 97/321 (30%) | 111/321 (35%) | 113/321 (35%) |
| eNOS Signaling | 0.138995 | 0.0291 | 47/172 (27%) | 50/172 (29%) | 74/172 (43%) |
| GDNF Family Ligand-Receptor Interactions | 0.140929 | 0.0366 | 31/82 (38%) | 34/82 (41%) | 17/82 (21%) |
| Atherosclerosis Signaling | 0.142889 | 0.0315 | 25/127 (20%) | 32/127 (25%) | 70/127 (55%) |
| Renin-Angiotensin Signaling | 0.145881 | 0.0312 | 46/128 (36%) | 50/128 (39%) | 31/128 (24%) |
| MIF Regulation of Innate Immunity | 0.147911 | 0.0465 | 12/43 (28%) | 15/43 (35%) | 16/43 (37%) |
| Growth Hormone Signaling | 0.147911 | 0.0357 | 30/84 (36%) | 30/84 (36%) | 24/84 (29%) |
| Embryonic Stem Cell Differentiation into Cardiac Lineages | 0.147911 | 0.1 | 2/10 (20%) | 2/10 (20%) | 6/10 (60%) |
| GDP-glucose Biosynthesis | 0.147911 | 0.1 | 4/10 (40%) | 4/10 (40%) | 2/10 (20%) |
| Glycine Betaine Degradation | 0.147911 | 0.1 | 2/10 (20%) | 4/10 (40%) | 4/10 (40%) |
| PI3K/AKT Signaling | 0.152055 | 0.0308 | 47/130 (36%) | 66/130 (51%) | 16/130 (12%) |
| Regulation of IL-2 Expression in Activated and Anergic T Lymphocytes | 0.155955 | 0.0349 | 37/86 (43%) | 31/86 (36%) | 18/86 (21%) |
| CD28 Signaling in T Helper Cells | 0.158125 | 0.0303 | 36/132 (27%) | 44/132 (33%) | 52/132 (39%) |
| iNOS Signaling | 0.158855 | 0.0444 | 19/45 (42%) | 21/45 (47%) | 5/45 (11%) |
| Glucose and Glucose-1-phosphate Degradation | 0.161065 | 0.0909 | 4/11 (36%) | 4/11 (36%) | 3/11 (27%) |
| Dolichyl-diphosphooligosaccharide Biosynthesis | 0.161065 | 0.0909 | 6/11 (55%) | 3/11 (27%) | 2/11 (18%) |
| UDP-N-acetyl-D-galactosamine Biosynthesis II | 0.161065 | 0.0909 | 5/11 (45%) | 5/11 (45%) | 1/11 (9%) |
| G-Protein Coupled Receptor Signaling | 0.162181 | 0.0248 | 75/282 (27%) | 84/282 (30%) | 122/282 (43%) |
| Adipogenesis pathway | 0.164059 | 0.0299 | 45/134 (34%) | 60/134 (45%) | 28/134 (21%) |
| IL-3 Signaling | 0.167109 | 0.0337 | 38/89 (43%) | 33/89 (37%) | 17/89 (19%) |
| PI3K Signaling in B Lymphocytes | 0.169824 | 0.0294 | 55/136 (40%) | 48/136 (35%) | 33/136 (24%) |
| Altered T Cell and B Cell Signaling in Rheumatoid Arthritis | 0.171002 | 0.0333 | 9/90 (10%) | 20/90 (22%) | 61/90 (68%) |
| nNOS Signaling in Neurons | 0.171002 | 0.0426 | 15/47 (32%) | 13/47 (28%) | 19/47 (40%) |
| Role of Pattern Recognition Receptors in Recognition of Bacteria and Viruses | 0.172982 | 0.0292 | 36/137 (26%) | 39/137 (28%) | 62/137 (45%) |
| OX40 Signaling Pathway | 0.174985 | 0.033 | 9/91 (10%) | 23/91 (25%) | 59/91 (65%) |
| Hepatic Fibrosis / Hepatic Stellate Cell Activation | 0.176198 | 0.0267 | 64/187 (34%) | 53/187 (28%) | 70/187 (37%) |
| Fcγ Receptor-mediated Phagocytosis in Macrophages and Monocytes | 0.179061 | 0.0326 | 40/92 (43%) | 30/92 (33%) | 22/92 (24%) |
| α-Adrenergic Signaling | 0.18281 | 0.0323 | 37/93 (40%) | 31/93 (33%) | 24/93 (26%) |
| LPS-stimulated MAPK Signaling | 0.18281 | 0.0323 | 35/93 (38%) | 41/93 (44%) | 17/93 (18%) |
| IL-4 Signaling | 0.187068 | 0.0319 | 31/94 (33%) | 35/94 (37%) | 28/94 (30%) |
| Fatty Acid Activation | 0.187932 | 0.0769 | 5/13 (38%) | 4/13 (31%) | 4/13 (31%) |
| D-myo-inositol (1,4,5,6)-Tetrakisphosphate Biosynthesis | 0.194984 | 0.0278 | 56/144 (39%) | 47/144 (33%) | 40/144 (28%) |
| D-myo-inositol (3,4,5,6)-tetrakisphosphate Biosynthesis | 0.194984 | 0.0278 | 56/144 (39%) | 47/144 (33%) | 40/144 (28%) |
| B Cell Receptor Signaling | 0.194984 | 0.0258 | 57/194 (29%) | 78/194 (40%) | 57/194 (29%) |
| Opioid Signaling Pathway | 0.196789 | 0.0244 | 70/246 (28%) | 84/246 (34%) | 91/246 (37%) |
| Regulation of the Epithelial-Mesenchymal Transition Pathway | 0.198153 | 0.0256 | 65/195 (33%) | 70/195 (36%) | 59/195 (30%) |
| Isoleucine Degradation I | 0.199986 | 0.0714 | 10/14 (71%) | 4/14 (29%) | 0/14 (0%) |
| Androgen Biosynthesis | 0.199986 | 0.0714 | 1/14 (7%) | 4/14 (29%) | 9/14 (64%) |
| Colanic Acid Building Blocks Biosynthesis | 0.199986 | 0.0714 | 6/14 (43%) | 7/14 (50%) | 1/14 (7%) |
| Insulin Receptor Signaling | 0.201837 | 0.0274 | 54/146 (37%) | 63/146 (43%) | 28/146 (19%) |
| CD27 Signaling in Lymphocytes | 0.205116 | 0.0377 | 14/53 (26%) | 31/53 (58%) | 7/53 (13%) |
| Role of Cytokines in Mediating Communication between Immune Cells | 0.210863 | 0.037 | 2/54 (4%) | 7/54 (13%) | 45/54 (83%) |
| Superpathway of Citrulline Metabolism | 0.212814 | 0.0667 | 5/15 (33%) | 3/15 (20%) | 7/15 (47%) |
| 3-phosphoinositide Biosynthesis | 0.214783 | 0.0249 | 71/201 (35%) | 68/201 (34%) | 61/201 (30%) |
| Epithelial Adherens Junction Signaling | 0.214783 | 0.0267 | 63/150 (42%) | 49/150 (33%) | 38/150 (25%) |
| Th2 Pathway | 0.214783 | 0.0267 | 31/150 (21%) | 36/150 (24%) | 83/150 (55%) |
| **PPAR Signaling** | 0.215774 | 0.0297 | 31/101 (31%) | 49/101 (49%) | 21/101 (21%) |
| Colorectal Cancer Metastasis Signaling | 0.21677 | 0.0236 | 87/254 (34%) | 91/254 (36%) | 75/254 (30%) |
| IL-8 Signaling | 0.219786 | 0.0246 | 72/203 (35%) | 78/203 (38%) | 53/203 (26%) |
| RANK Signaling in Osteoclasts | 0.219786 | 0.0294 | 34/102 (33%) | 45/102 (44%) | 22/102 (22%) |
| NER Pathway | 0.223872 | 0.0291 | 44/103 (43%) | 45/103 (44%) | 14/103 (14%) |
| Gustation Pathway | 0.228034 | 0.026 | 33/154 (21%) | 38/154 (25%) | 82/154 (53%) |
| mTOR Signaling | 0.229087 | 0.0243 | 80/206 (39%) | 86/206 (42%) | 39/206 (19%) |
| Clathrin-mediated Endocytosis Signaling | 0.231739 | 0.0242 | 67/207 (32%) | 70/207 (34%) | 68/207 (33%) |
| RAN Signaling | 0.238232 | 0.0588 | 11/17 (65%) | 4/17 (24%) | 2/17 (12%) |
| γ-linolenate Biosynthesis II (Animals) | 0.238232 | 0.0588 | 8/17 (47%) | 4/17 (24%) | 5/17 (29%) |
| Mitochondrial L-carnitine Shuttle Pathway | 0.238232 | 0.0588 | 6/17 (35%) | 6/17 (35%) | 5/17 (29%) |
| 3-phosphoinositide Degradation | 0.242103 | 0.0253 | 59/158 (37%) | 53/158 (34%) | 45/158 (28%) |
| Differential Regulation of Cytokine Production in Macrophages and T Helper Cells by IL-17A and IL-17F | 0.250035 | 0.0556 | 1/18 (6%) | 4/18 (22%) | 13/18 (72%) |
| Ubiquinol-10 Biosynthesis (Eukaryotic) | 0.250035 | 0.0556 | 3/18 (17%) | 7/18 (39%) | 8/18 (44%) |
| Valine Degradation I | 0.250035 | 0.0556 | 14/18 (78%) | 4/18 (22%) | 0/18 (0%) |
| Apelin Muscle Signaling Pathway | 0.250035 | 0.0556 | 6/18 (33%) | 8/18 (44%) | 4/18 (22%) |
| Induction of Apoptosis by HIV1 | 0.251768 | 0.0328 | 26/61 (43%) | 26/61 (43%) | 9/61 (15%) |
| Gαq Signaling | 0.251768 | 0.0248 | 54/161 (34%) | 57/161 (35%) | 50/161 (31%) |
| D-myo-inositol-5-phosphate Metabolism | 0.255859 | 0.0247 | 62/162 (38%) | 50/162 (31%) | 49/162 (30%) |
| Type I Diabetes Mellitus Signaling | 0.258226 | 0.027 | 30/111 (27%) | 40/111 (36%) | 41/111 (37%) |
| GPCR-Mediated Nutrient Sensing in Enteroendocrine Cells | 0.261818 | 0.0268 | 25/112 (22%) | 33/112 (29%) | 53/112 (47%) |
| Cdc42 Signaling | 0.272898 | 0.024 | 49/167 (29%) | 45/167 (27%) | 73/167 (44%) |
| Granzyme A Signaling | 0.274157 | 0.05 | 4/20 (20%) | 4/20 (20%) | 11/20 (55%) |
| Inflammasome pathway | 0.274157 | 0.05 | 7/20 (35%) | 5/20 (25%) | 8/20 (40%) |
| Nicotine Degradation II | 0.274789 | 0.0308 | 6/65 (9%) | 10/65 (15%) | 49/65 (75%) |
| UVB-Induced MAPK Signaling | 0.28119 | 0.0303 | 24/66 (36%) | 25/66 (38%) | 17/66 (26%) |
| Telomerase Signaling | 0.283139 | 0.0256 | 42/117 (36%) | 50/117 (43%) | 25/117 (21%) |
| Maturity Onset Diabetes of Young (MODY) Signaling | 0.285102 | 0.0476 | 1/21 (5%) | 4/21 (19%) | 16/21 (76%) |
| HGF Signaling | 0.291743 | 0.0252 | 48/119 (40%) | 48/119 (40%) | 22/119 (18%) |
| EGF Signaling | 0.293089 | 0.0294 | 28/68 (41%) | 30/68 (44%) | 10/68 (15%) |
| cAMP-mediated signaling | 0.295121 | 0.0219 | 61/228 (27%) | 47/228 (21%) | 119/228 (52%) |
| Polyamine Regulation in Colon Cancer | 0.295801 | 0.0455 | 11/22 (50%) | 8/22 (36%) | 3/22 (14%) |
| Glioma Signaling | 0.295801 | 0.025 | 51/120 (43%) | 48/120 (40%) | 21/120 (18%) |
| Neuroprotective Role of THOP1 in Alzheimer's Disease | 0.295801 | 0.025 | 18/120 (15%) | 23/120 (19%) | 79/120 (66%) |
| **LXR/RXR Activation** | 0.301301 | 0.0248 | 29/121 (24%) | 32/121 (26%) | 60/121 (50%) |

**Supplemental Table 9**. Ingenuity toxicity pathways activated by 20(OH)C in murine fibroblasts, nuclear receptors are in bold

| **Ingenuity Toxicity Lists** | **p value** | **Ratio** |
| --- | --- | --- |
| **RAR Activation** | 0.000933 | 0.0526 |
| Hepatic Cholestasis | 0.004571 | 0.0491 |
| LPS/IL-1 Mediated Inhibition of RXR Function | 0.007762 | 0.0392 |
| NF-κB Signaling | 0.012882 | 0.0362 |
| Hepatic Fibrosis | 0.025704 | 0.0476 |
| Xenobiotic Metabolism Signaling | 0.0302 | 0.0304 |
| Increases Transmembrane Potential of Mitochondria and Mitochondrial Membrane | 0.044668 | 0.06 |
| Cardiac Necrosis/Cell Death | 0.047863 | 0.0303 |
| Renal Necrosis/Cell Death | 0.056234 | 0.0255 |
| Cardiac Fibrosis | 0.058884 | 0.0321 |
| Cardiac Hypertrophy | 0.063096 | 0.0277 |
| Cell Cycle: G1/S Checkpoint Regulation | 0.095499 | 0.0435 |
| Cytochrome P450 Panel - Substrate is a Vitamin (Mouse) | 0.105682 | 0.143 |
| Hepatic Stellate Cell Activation | 0.105925 | 0.0571 |
| NRF2-mediated Oxidative Stress Response | 0.106905 | 0.0278 |
| Fatty Acid Metabolism | 0.115345 | 0.0342 |
| Glutathione Depletion - Hepatocellular Hypertrophy | 0.11995 | 0.125 |
| Vasopressin-induced Genes in Inner Medullary Renal Collecting Duct Cells (Rat) | 0.11995 | 0.125 |
| **VDR/RXR Activation** | 0.126765 | 0.0385 |
| Renal Ischemic Resistance Panel (Rat) | 0.147571 | 0.1 |
| Long-term Renal Injury Pro-oxidative Response Panel (Rat) | 0.187499 | 0.0769 |
| Mechanism of Gene Regulation by Peroxisome Proliferators via PPARα | 0.190985 | 0.0316 |
| Increases Renal Damage | 0.190985 | 0.0316 |
| Increases Liver Steatosis | 0.228034 | 0.0288 |
| Increases Depolarization of Mitochondria and Mitochondrial Membrane | 0.250035 | 0.0556 |
| Long-term Renal Injury Anti-oxidative Response Panel (Rat) | 0.250035 | 0.0556 |
| Glutathione Depletion - Phase II Reactions | 0.273527 | 0.05 |
| Genes associated with Chronic Allograft Nephropathy (Human) | 0.285102 | 0.0476 |
| Nongenotoxic Hepatocarcinogenicity Biomarker Panel | 0.296483 | 0.0455 |
| **LXR/RXR Activation** | 0.30903 | 0.0244 |
| Increases Liver Damage | 0.317687 | 0.024 |
| **FXR/RXR Activation** | 0.322107 | 0.0238 |
| Increases Liver Hyperplasia/Hyperproliferation | 0.334965 | 0.0233 |
| **PPARα/RXRα Activation** | 0.35156 | 0.0212 |
| **CAR/RXR Activation** | 0.370681 | 0.0345 |
| Positive Acute Phase Response Proteins | 0.381066 | 0.0333 |
| Increases Glomerular Injury | 0.390841 | 0.0235 |
| Decreases Depolarization of Mitochondria and Mitochondrial Membrane | 0.399945 | 0.0312 |
| **TR/RXR Activation** | 0.461318 | 0.0204 |
| **Aryl Hydrocarbon Receptor Signaling** | 0.470977 | 0.0186 |

**Supplemental Table 10.** Gene Oncology Enrichment Analysis (Panther platform) of activation of LXR pathways in relation to o VDR signaling by 20,23(OH)_2_D3

| **GO molecular function complete** | **fold Enrichment** | **raw P-value** | **FDR** |
| --- | --- | --- | --- |
| vitamin D response element binding | 1.10 | 1.00E00 | 1.00E00 |
| low-density lipoprotein particle receptor activity | 1.10 | 8.27E-01 | 1.00E00 |
| lipoprotein lipase activity | 1.10 | 1.00E00 | 1.00E00 |
| fatty acid synthase activity | 1.10 | 8.34E-01 | 1.00E00 |
| ATPase-coupled intramembrane lipid transporter activity | 1.10 | 7.82E-01 | 1.00E00 |
| retinoid X receptor binding | 1.10 | 7.60E-01 | 1.00E00 |
| bioactive lipid receptor activity | 1.10 | 8.56E-01 | 1.00E00 |
| S100 protein binding | 1.10 | 8.51E-01 | 1.00E00 |
| retinoic acid-responsive element binding | 1.10 | 1.00E00 | 1.00E00 |
| low-density lipoprotein particle binding | 1.10 | 7.55E-01 | 1.00E00 |
| oxysterol binding | 1.10 | 1.00E00 | 1.00E00 |
| high-density lipoprotein particle receptor activity | 1.10 | 1.00E00 | 1.00E00 |
| vitamin D binding | 1.10 | 1.00E00 | 1.00E00 |
| high-density lipoprotein particle binding | 1.10 | 8.34E-01 | 1.00E00 |
| cholesterol transfer activity | 1.10 | 7.55E-01 | 1.00E00 |
| apolipoprotein A-I binding | 1.10 | 1.00E00 | 1.00E00 |
| apolipoprotein binding | 1.10 | 8.68E-01 | 1.00E00 |
| aryl hydrocarbon receptor binding | 1.10 | 8.27E-01 | 1.00E00 |
| peroxisome proliferator activated receptor binding | 1.10 | 7.55E-01 | 1.00E00 |
| ATPase-coupled lipid transmembrane transporter activity | 1.10 | 1.00E00 | 1.00E00 |
| lipoprotein particle binding | 1.10 | 7.05E-01 | 1.00E00 |
| vitamin D receptor binding | 1.10 | 1.00E00 | 1.00E00 |
| interleukin-1 type II receptor antagonist activity | 1.10 | 1.00E00 | 1.00E00 |
| hydroxymethylglutaryl-CoA synthase activity | 1.10 | 1.00E00 | 1.00E00 |
| hydroxymethylglutaryl-CoA reductase (NADPH) activity | 1.10 | 1.00E00 | 1.00E00 |
| fatty-acyl-CoA binding | 1.10 | 7.78E-01 | 1.00E00 |
| bile acid receptor activity | 1.10 | 1.00E00 | 1.00E00 |
| sterol response element binding | 1.10 | 1.00E00 | 1.00E00 |
| chylomicron binding | 1.10 | 1.00E00 | 1.00E00 |
| lipase binding | 1.10 | 1.00E00 | 1.00E00 |
| steroid receptor RNA activator RNA binding | 1.10 | 1.00E00 | 1.00E00 |
| fatty-acyl-CoA synthase activity | 1.10 | 1.00E00 | 1.00E00 |
| farnesyl-diphosphate farnesyltransferase activity | 1.10 | 1.00E00 | 1.00E00 |
| ABC-type transmembrane transporter activity | 1.10 | 1.00E00 | 1.00E00 |
| cholesterol 26-hydroxylase activity | 1.10 | 1.00E00 | 1.00E00 |
| oxidised low-density lipoprotein particle receptor activity | 1.10 | 1.00E00 | 1.00E00 |
| cholesterol 25-hydroxylase activity | 1.10 | 1.00E00 | 1.00E00 |
| cholesterol O-acyltransferase activity | 1.10 | 1.00E00 | 1.00E00 |
| vitamin D3 25-hydroxylase activity | 1.10 | 1.00E00 | 1.00E00 |
| 1-alpha,25-dihydroxyvitamin D3 24-hydroxylase activity | 1.10 | 1.00E00 | 1.00E00 |
| fatty-acyl-ethyl-ester synthase activity | 1.10 | 1.00E00 | 1.00E00 |
| 25-hydroxycholecalciferol-24-hydroxylase activity | 1.10 | 1.00E00 | 1.00E00 |
| interleukin-10 binding | 1.10 | 1.00E00 | 1.00E00 |
| hydroxymethylglutaryl-CoA reductase activity | 1.10 | 1.00E00 | 1.00E00 |
| retinol transmembrane transporter activity | 1.10 | 1.00E00 | 1.00E00 |
| nitric-oxide synthase regulator activity | 1.10 | 1.00E00 | 1.00E00 |
| very-low-density lipoprotein particle receptor activity | 1.10 | 1.00E00 | 1.00E00 |
| apolipoprotein receptor activity | 1.10 | 1.00E00 | 1.00E00 |
| mineralocorticoid receptor binding | 1.10 | 1.00E00 | 1.00E00 |
| high-density lipoprotein particle receptor binding | 1.10 | 1.00E00 | 1.00E00 |
| vitamin D 25-hydroxylase activity | 1.10 | 1.00E00 | 1.00E00 |
| D3 vitamins binding | 1.10 | 1.00E00 | 1.00E00 |
| calcidiol binding | 1.10 | 1.00E00 | 1.00E00 |
| fatty acid alpha-hydroxylase activity | 1.10 | 1.00E00 | 1.00E00 |
| very-low-density lipoprotein particle receptor binding | 1.10 | 1.00E00 | 1.00E00 |
| bile acid binding | 1.10 | 8.40E-01 | 1.00E00 |
| glucocorticoid receptor activity | 1.10 | 1.00E00 | 1.00E00 |
| apolipoprotein A-I receptor binding | 1.10 | 1.00E00 | 1.00E00 |
| apolipoprotein receptor binding | 1.10 | 1.00E00 | 1.00E00 |
| very-low-density lipoprotein particle binding | 1.10 | 1.00E00 | 1.00E00 |
| apolipoprotein A-I receptor activity | 1.10 | 1.00E00 | 1.00E00 |
| steroid hormone receptor activity | 1.08 | 6.99E-01 | 1.00E00 |
| cholesterol binding | 1.08 | 7.58E-01 | 1.00E00 |
| lipid transfer activity | 1.08 | 7.48E-01 | 1.00E00 |
| lipid transporter activity | 1.07 | 5.61E-01 | 1.00E00 |
| lipoprotein particle receptor binding | 1.07 | 8.02E-01 | 1.00E00 |
| lipase activity | 1.07 | 6.12E-01 | 1.00E00 |
| retinoic acid receptor binding | 1.07 | 7.99E-01 | 1.00E00 |
| low-density lipoprotein particle receptor binding | 1.06 | 8.93E-01 | 1.00E00 |
| acyl-CoA binding | 1.06 | 8.93E-01 | 1.00E00 |
| nitric-oxide synthase binding | 1.05 | 8.80E-01 | 1.00E00 |
| lipid binding | 1.05 | 3.52E-01 | 1.00E00 |
| lipoprotein particle receptor activity | 1.03 | 1.00E00 | 1.00E00 |
| retinoid binding | 1.01 | 1.00E00 | 1.00E00 |
| lipase activator activity | 1.00 | 1.00E00 | 1.00E00 |
| bile acid transmembrane transporter activity | 0.95 | 1.00E00 | 1.00E00 |
| lipoprotein lipase activator activity | 0.74 | 1.00E00 | 1.00E00 |
| acetyl-CoA binding | 0.74 | 1.00E00 | 1.00E00 |

**Molecular docking studies of a ligand series potentially targeting LXRα and LXRβ**

Among LXR crystal structures, two LXRα (PDBID: 5AVI, 3IPQ) and three LXRβ (PDBID: PQC, 1UPV, 5HJP) that have most structurally diverse conformations (Fig. S1) were selected for docking studies. The choice of these crystal structures took into account alternative ligand binding modes that could come out of exploring different conformations of the binding pocket.


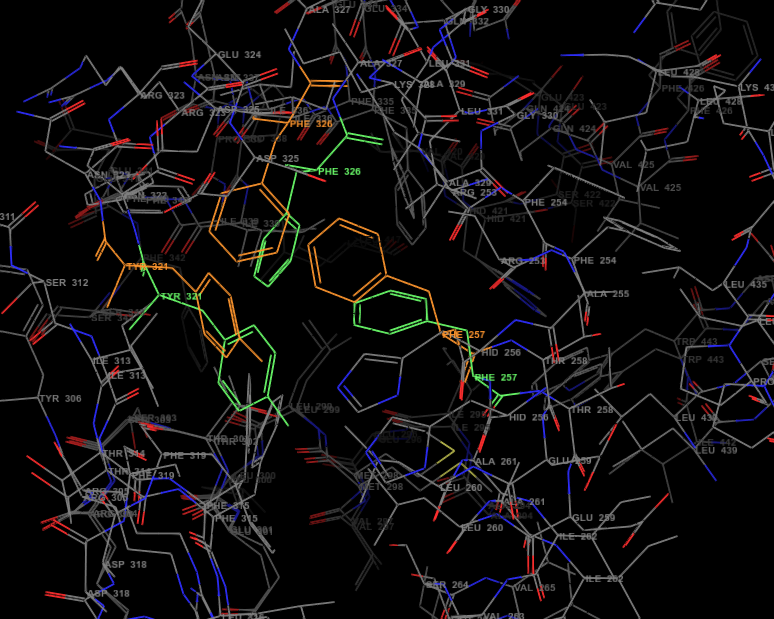


**Fig. S1**. Superposition of two LXRα conformations (PDBID:5AVI and 3IPQ) as an example of the LXR active site flexibility. Residues displaying significant difference of their side chain positions are highlighted.


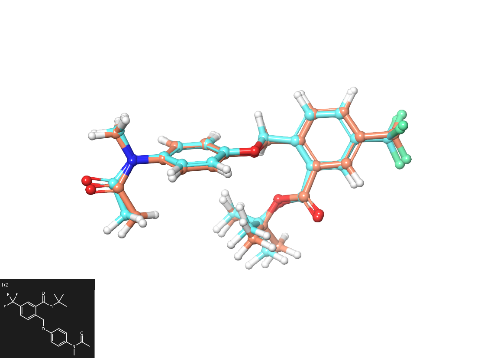
At the initial stage, the co-crystalized ligands (i.e., those found in 5AVI, 3IPQ, 5HJP, 1PQC, 1UPV) have been re-docked into the corresponding binding site to validate the docking procedure. All the corresponding ligand pairs (docked VS co-crystalized) displayed RMSD in the range 0.5 Å - 0.7 Å (acceptable values are ≤ 2.0 Å), i.e., the docking reproduced the co-crystalized (experimental) ligand poses with high precision (Fig. S2).

**Fig. S2**. Tert-butyl 2-[[4-[ethanoyl(methyl)amino]phenoxy]methyl]-5-(trifluoromethyl)benzoate. Example of docked (cyan) VS co-crystalized (orange) poses based on 5AVI (LXRα). RMSD = 0.4061Å

After the calculations, all 84 compounds have been manually inspected for favorable positions and conformations in LXRα and LXRβ binding site, presence of intermolecular H-bonds, π-π interactions, hydrophobic interactions and steric clashes. Generally, all compounds bind tightly due to mainly hydrophobic interactions and intermolecular hydrogen bonds (see examples below). Due to compounds’ hydrophobic nature they fit well the hydrophobic cavity of the binding site (Fig. S3).


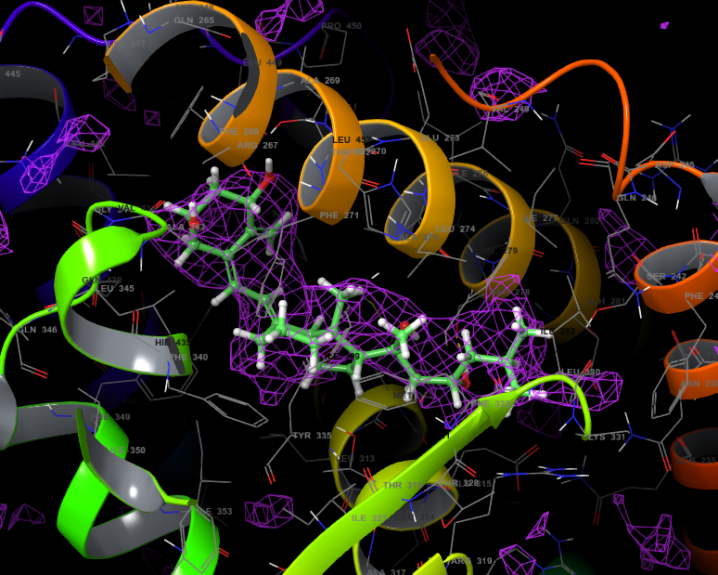


**Fig. S3**. 1α,20*S*(OH)_2_D3 (LXR-20077) in 5HJP (LXRβ) binding site. Magenta mesh is the hydrophobic cavities in the protein’s structure.

*Ligand Binding to LXRα*

Depending on the receptor conformation, the ligands display two general binding modes. LXR binding site is a stretched, predominantly hydrophobic binding cavity, and according to the docking results, the same ligand could “flip” between the two “opposite” binding modes according to the receptor conformation used for the docking and the ligand’s structure (Fig. S4).

| 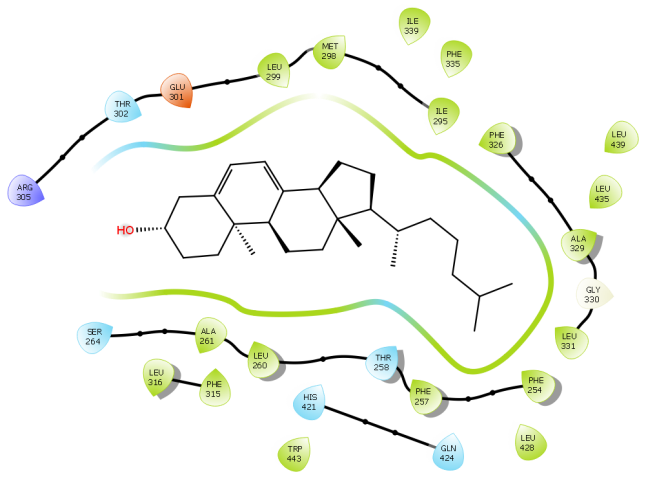  A | 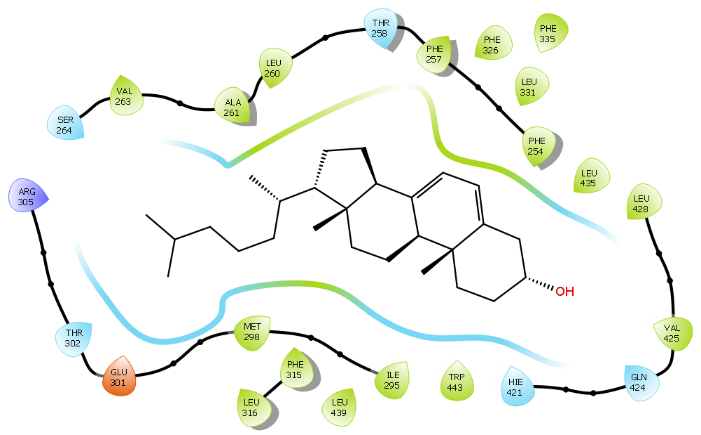  B |
| --- | --- |

**Fig. S4.** 7-Dehydrocholesterol in 5AVI (A) and 3IPQ (B) LXRα LBD pocket.

The LBD pocket consists of five cavities (C1, C2, C3, C4 and SC) according to the classification described in literature^1^. While having the hydrophobic “central” part, two main hydrogen bonding regions could be allocated in the LBD active site structure as well. These regions are surrounding the central part of the site. One of them is formed by residues Glu267, Ser264, Thr302, Arg305, Leu316, Leu260, Met298), and another one by Ala329, Phe257, Phe326. Majority of the tested ligands position their heterocyclic hydrophobic core in the central part of the binding site, while having their donor\acceptor moieties (hydroxyl and carbonyl groups) bonded to either one or both of the hydrogen bonding regions. A special attention should be paid to the hydrophobic cavity C1 (formed by residues Phe254, Thr258, Met298, Leu331, Phe335, Leu428, Leu435, Trp443, and His421) which accommodates bulk aliphatic moieties, such as 2,6-dimethylhexane in lumisterol molecule. This produces strong hydrophobic interaction that could contribute a lot to the overall ligand binding affinity. Detailed information about the hydrogen bonds and hydrophobic interaction for all 84 ligand docked into LXRα LBD pocket is represented in the Supplemental Tables 11 and 12.

For example, 22*R*(OH)C forms two hydrogen bonds – one between its OH group in position 3 of the core and Glu267 of the CS cavity, and another one is between 22*R*-hydroxyl group and backbone of Phe257 (C4 cavity). The four fused rings (A, B, C and D) of the steroid core form hydrophobic contacts in the central part of the LBD pocket, mainly with Leu315, Leu260, Met298 and Phe257 of C3 and C4 cavities. The ligand’s 5-hydroxy-2,6-dimethylhexane moiety occupies the hydrophobic C1 cavity (Fig S5). Similar position in active site displays 22*R*(OH)C (Fig. S6), except forming only one hydrogen bond (with main chain of Leu260).


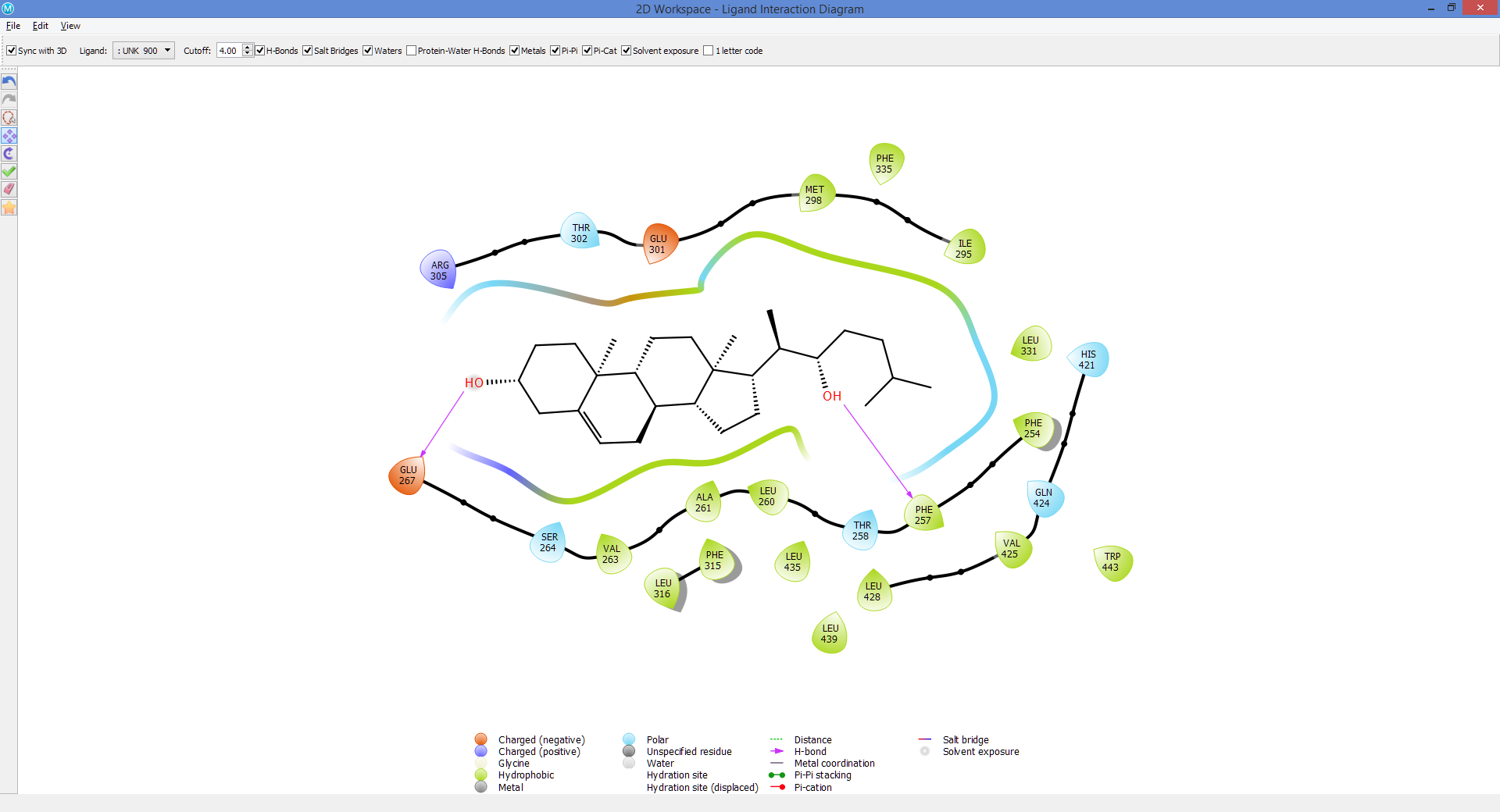


(A)


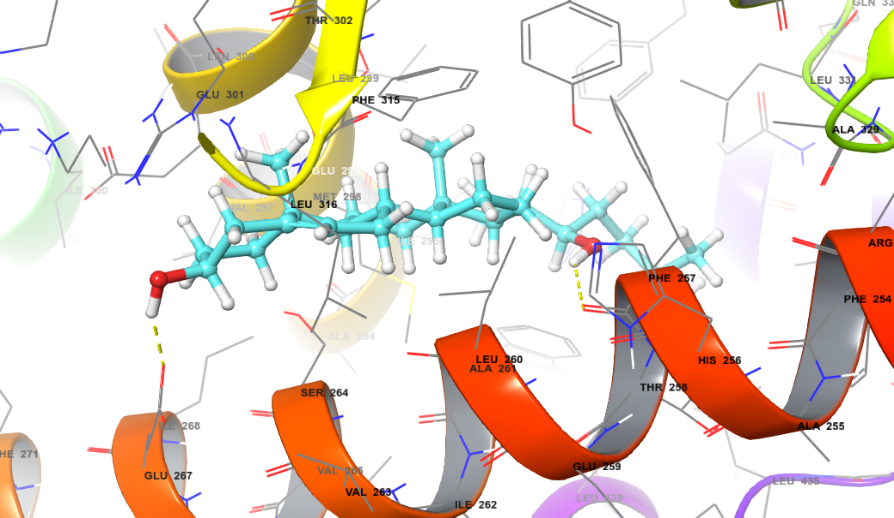


(B)

**Fig. S5.** 22*R*(OH) cholesterol (LXR-20015) in 5AVI LRXα LBD pocket. A, 2D interaction diagram. B, 3D view of the complex.


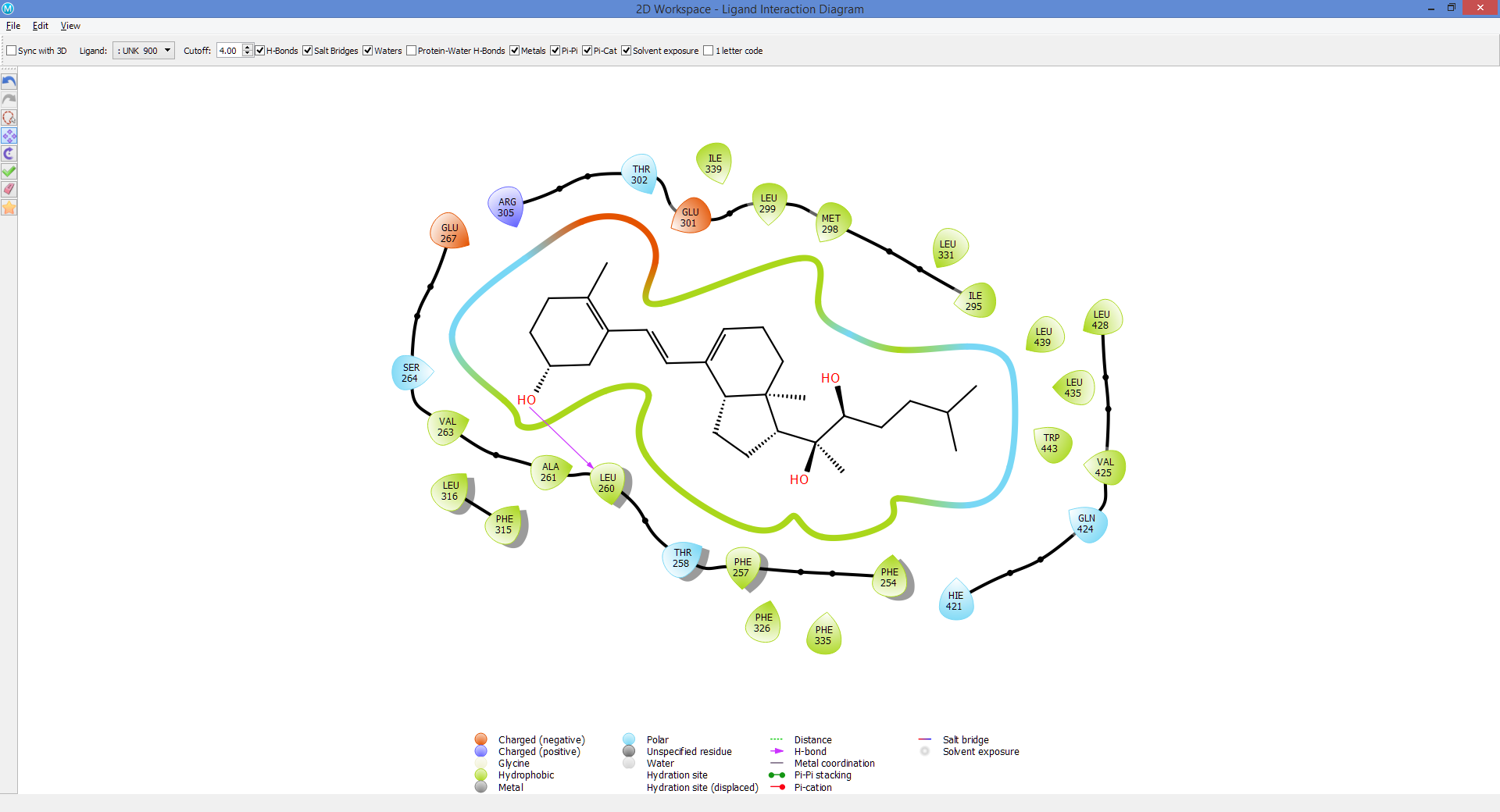


(A)


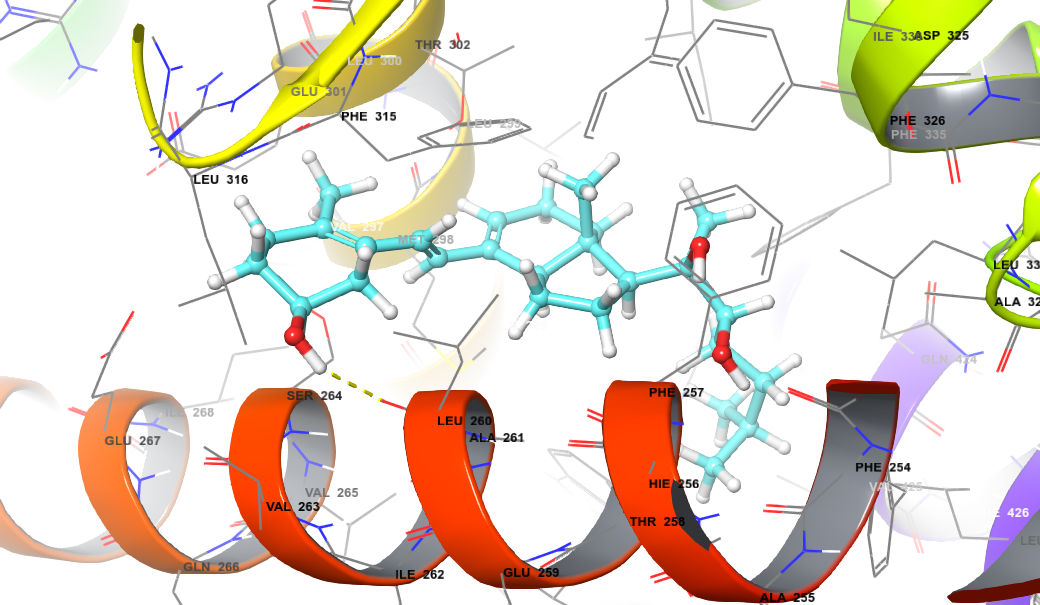


(B)

**Fig. S6.** 20*S*,22*S*(OH)2T3 (LXR-20053) in 3IPQ LRXα LBD pocket. A, 2D interaction diagram. B, 3D view of the complex.

**Supplemental Table 11.** Summary of hydrogen bonds and hydrophobic interactions of 84 ligands docked into LXRα LBD pocket (conformation of PDBID: 5AVI).

|  | **Type** | **Residue** | **# of ligands involved** | **# of contacts** |
| --- | --- | --- | --- | --- |
|  | HBond | A:257(PHE) | 2 | 2 |
|  | HBond | A:260(LEU) | 4 | 4 |
|  | HBond | A:264(SER) | 12 | 12 |
|  | HBond | A:267(GLU) | 50 | 56 |
|  | HBond | A:298(MET) | 2 | 2 |
|  | HBond | A:302(THR) | 2 | 2 |
|  | HBond | A:305(ARG) | 3 | 3 |
|  | HBond | A:316(LEU) | 5 | 5 |
|  | HBond | A:329(ALA) | 3 | 3 |
| Total Hydrogen Bonds interactions: | | | | 89 |
|  |  |  |  |  |
|  | **Type** | **Residue** | **# of ligands involved** | **# of contacts** |
|  | HPhob | A:254(PHE) | 26 | 58 |
|  | HPhob | A:257(PHE) | 84 | 654 |
|  | HPhob | A:258(THR) | 50 | 97 |
|  | HPhob | A:260(LEU) | 77 | 277 |
|  | HPhob | A:261(ALA) | 54 | 72 |
|  | HPhob | A:263(VAL) | 10 | 14 |
|  | HPhob | A:295(ILE) | 48 | 60 |
|  | HPhob | A:298(MET) | 84 | 361 |
|  | HPhob | A:299(LEU) | 3 | 3 |
|  | HPhob | A:301(GLU) | 61 | 98 |
|  | HPhob | A:302(THR) | 2 | 2 |
|  | HPhob | A:315(PHE) | 83 | 1279 |
|  | HPhob | A:316(LEU) | 11 | 18 |
|  | HPhob | A:317(LYS) | 1 | 1 |
|  | HPhob | A:326(PHE) | 20 | 21 |
|  | HPhob | A:331(LEU) | 70 | 180 |
|  | HPhob | A:335(PHE) | 38 | 89 |
|  | HPhob | A:339(ILE) | 6 | 7 |
|  | HPhob | A:424(GLN) | 13 | 13 |
|  | HPhob | A:425(VAL) | 5 | 5 |
|  | HPhob | A:428(LEU) | 44 | 59 |
|  | HPhob | A:435(LEU) | 21 | 37 |
|  | HPhob | A:439(LEU) | 4 | 5 |
|  | HPhob | A:443(TRP) | 57 | 155 |
| Total Hydrophobic interactions: | | | | 3565 |

**Supplemental Table 12.** Summary of hydrogen bonds and hydrophobic interactions of 84 ligands docked into LXRα LBD pocket (conformation of PDBID: 3IPQ).

|  | **Type** | **Residue** | **# of ligands involved** | **# of contacts** |
| --- | --- | --- | --- | --- |
|  | HBond | A:260(LEU) | 12 | 13 |
|  | HBond | A:264(SER) | 5 | 5 |
|  | HBond | A:267(GLU) | 19 | 19 |
|  | HBond | A:302(THR) | 4 | 4 |
|  | HBond | A:305(ARG) | 14 | 14 |
|  | HBond | A:316(LEU) | 7 | 7 |
| Total Hydrogen Bonds interactions: | | | | 62 |
|  |  |  |  |  |
|  | **Type** | **Residue** | **# of ligands involved** | **# of contacts** |
|  | HPhob | A:254(PHE) | 26 | 54 |
|  | HPhob | A:257(PHE) | 65 | 182 |
|  | HPhob | A:258(THR) | 41 | 61 |
|  | HPhob | A:260(LEU) | 65 | 175 |
|  | HPhob | A:261(ALA) | 75 | 159 |
|  | HPhob | A:263(VAL) | 13 | 22 |
|  | HPhob | A:267(GLU) | 1 | 3 |
|  | HPhob | A:268(ILE) | 1 | 2 |
|  | HPhob | A:295(ILE) | 9 | 11 |
|  | HPhob | A:298(MET) | 84 | 274 |
|  | HPhob | A:301(GLU) | 33 | 54 |
|  | HPhob | A:302(THR) | 27 | 37 |
|  | HPhob | A:313(ILE) | 8 | 11 |
|  | HPhob | A:315(PHE) | 82 | 1406 |
|  | HPhob | A:316(LEU) | 28 | 71 |
|  | HPhob | A:326(PHE) | 55 | 297 |
|  | HPhob | A:331(LEU) | 64 | 219 |
|  | HPhob | A:335(PHE) | 78 | 333 |
|  | HPhob | A:336(ILE) | 12 | 23 |
|  | HPhob | A:339(ILE) | 21 | 84 |
|  | HPhob | A:340(PHE) | 11 | 26 |
|  | HPhob | A:424(GLN) | 26 | 29 |
|  | HPhob | A:425(VAL) | 28 | 28 |
|  | HPhob | A:428(LEU) | 45 | 60 |
|  | HPhob | A:435(LEU) | 41 | 61 |
|  | HPhob | A:439(LEU) | 13 | 13 |
|  | HPhob | A:443(TRP) | 58 | 236 |
| Total Hydrophobic interactions: | | | | 3931 |

*Ligand Binding to LXRβ*

Similarly to LXRα, the ligand-binding pocket of LXRβ is hydrophobic but with polar or charged residues at the two ends of the cavity that form analogous hydrogen bonding regions. The pocket is flexible and can adjust to accommodate structurally different ligands. In case of LXRβ, the tested ligands bind generally similar as in case of LXRα displaying two “opposite” binding modes, forming hydrogen bonds with the abovementioned hydrogen bonding regions (predominantly Leu274, Glu281, Glu315, Thr316, His435, Thr272, Ser278, Ser242) and hydrophobic interactions in core region of the pocket and with the hydrophobic cavity C1^1^. See examples on Fig. S7 and S8. Detailed information about the hydrogen bonds and hydrophobic interaction for all 84 ligand docked into LXRα LBD pocket is represented in the Supplemental Tables 13-15.

A B


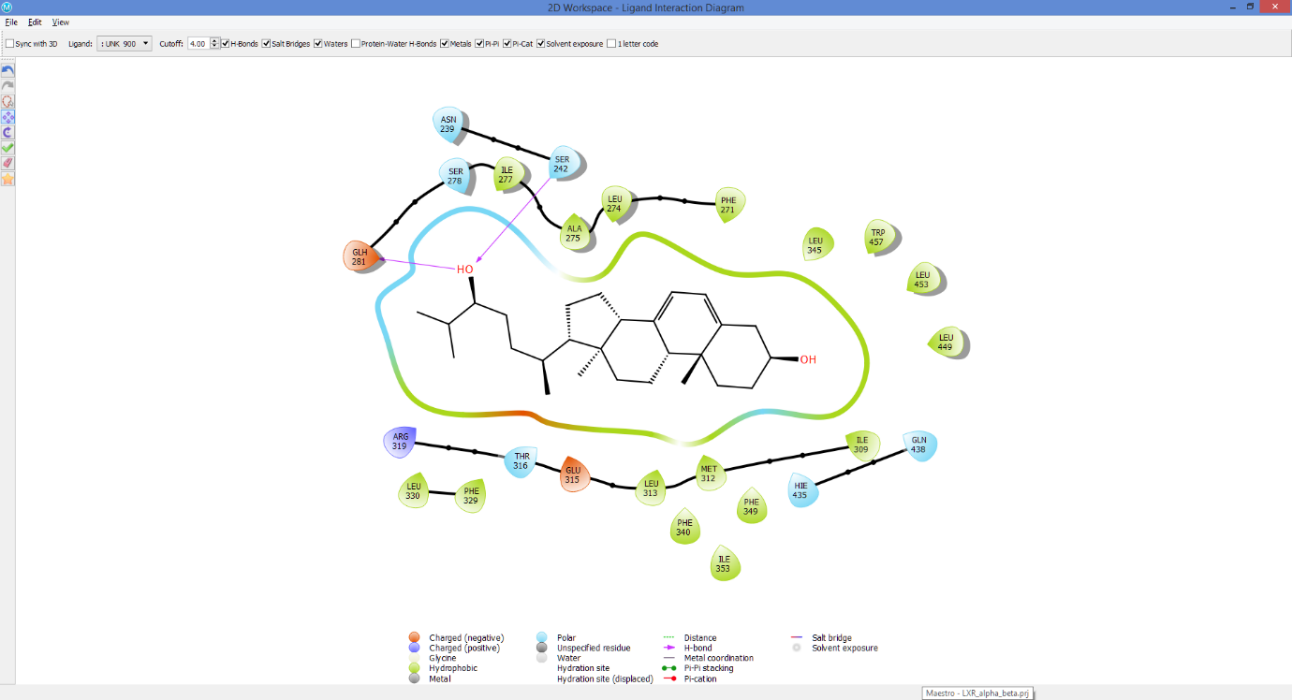


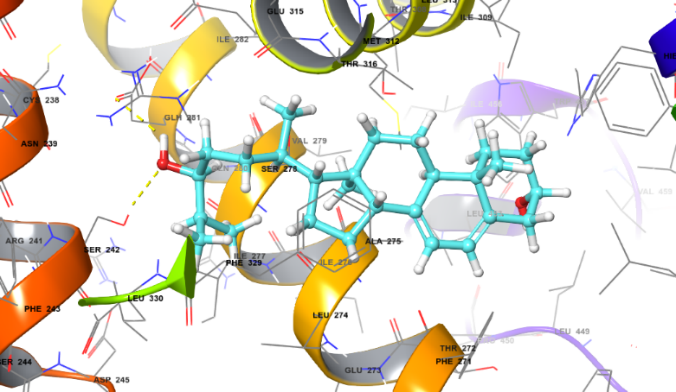


**Fig. S7.** 24*S*(OH)L3 in 5HJP LRXβ LBD pocket. A, 2D interaction diagram. B, 3D view of the complex.

A B


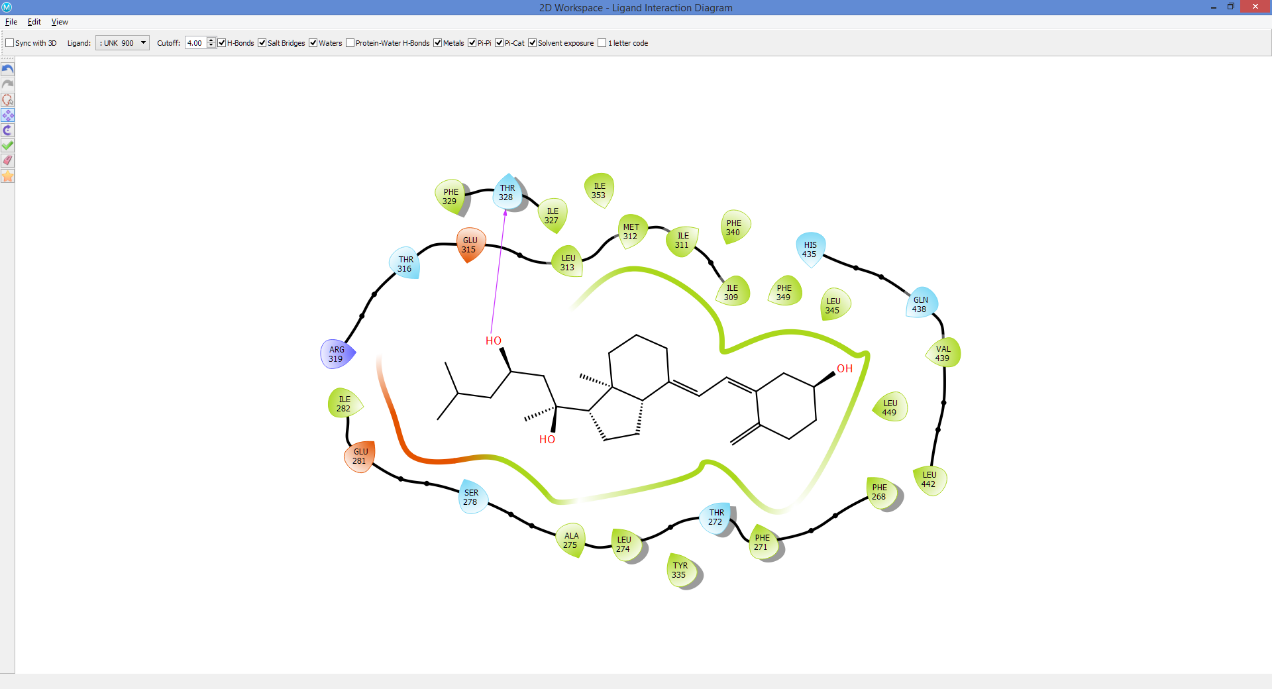


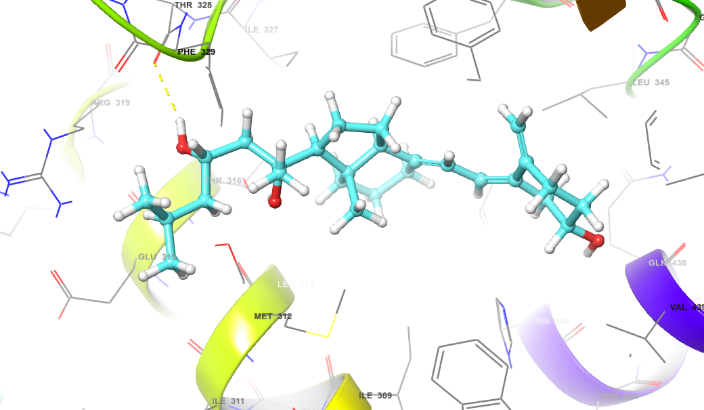


**Fig. S8.** 20*S*,23*S*(OH)_2_D3 (LXR-20071) in 1UPV LRXβ LBD pocket. A, 2D interaction diagram. B, 3D view of the complex.

**Supplemental Table 13.** Summary of hydrogen bonds and hydrophobic interactions of 84 ligands docked into LXRβ LBD pocket (conformation of PDBID: 5HJP).

|  | **Type** | **Residue** | **# of ligands involved** | **# of contacts** |
| --- | --- | --- | --- | --- |
|  | HBond | B:242(SER) | 8 | 8 |
|  | HBond | B:268(PHE) | 1 | 1 |
|  | HBond | B:274(LEU) | 12 | 12 |
|  | HBond | B:278(SER) | 9 | 9 |
|  | HBond | B:281(GLH) | 17 | 17 |
|  | HBond | B:312(MET) | 5 | 5 |
|  | HBond | B:315(GLU) | 10 | 10 |
|  | HBond | B:316(THR) | 1 | 1 |
|  | HBond | B:330(LEU) | 2 | 2 |
|  | HBond | B:343(ALA) | 5 | 5 |
|  | HBond | B:435(HIE) | 7 | 7 |
| Total Hydrogen Bonds interactions: | | | | 77 |
|  |  |  |  |  |
|  | **Type** | **Residue** | **# of ligands involved** | **# of contacts** |
|  | HPhob | B:268(PHE) | 30 | 103 |
|  | HPhob | B:271(PHE) | 78 | 479 |
|  | HPhob | B:274(LEU) | 77 | 241 |
|  | HPhob | B:275(ALA) | 78 | 110 |
|  | HPhob | B:277(ILE) | 15 | 20 |
|  | HPhob | B:281(GLH) | 1 | 2 |
|  | HPhob | B:309(ILE) | 18 | 26 |
|  | HPhob | B:312(MET) | 82 | 291 |
|  | HPhob | B:313(LEU) | 13 | 15 |
|  | HPhob | B:315(GLU) | 49 | 112 |
|  | HPhob | B:316(THR) | 1 | 1 |
|  | HPhob | B:329(PHE) | 83 | 1591 |
|  | HPhob | B:330(LEU) | 31 | 51 |
|  | HPhob | B:340(PHE) | 41 | 48 |
|  | HPhob | B:345(LEU) | 55 | 146 |
|  | HPhob | B:349(PHE) | 44 | 89 |
|  | HPhob | B:353(ILE) | 11 | 12 |
|  | HPhob | B:438(GLN) | 28 | 32 |
|  | HPhob | B:439(VAL) | 9 | 9 |
|  | HPhob | B:442(LEU) | 56 | 61 |
|  | HPhob | B:449(LEU) | 39 | 60 |
|  | HPhob | B:453(LEU) | 4 | 4 |
|  | HPhob | B:457(TRP) | 56 | 120 |
| Total Hydrophobic interactions: | | | | 3623 |

**Supplemental Table 14.** Summary of hydrogen bonds and hydrophobic interactions of 84 ligands docked into LXRβ LBD pocket (conformation of PDBID: 1PQC).

|  | **Type** | **Residue** | **# of ligands involved** | **# of contacts** |
| --- | --- | --- | --- | --- |
|  | HBond | C:241(ARG) | 8 | 13 |
|  | HBond | C:272(THR) | 1 | 1 |
|  | HBond | C:278(SER) | 6 | 6 |
|  | HBond | C:312(MET) | 1 | 1 |
|  | HBond | C:316(THR) | 13 | 14 |
|  | HBond | C:435(HIE) | 16 | 16 |
| Total Hydrogen Bonds interactions: | | | | 51 |
|  |  |  |  |  |
|  | **Type** | **Residue** | **# of ligands involved** | **# of contacts** |
|  | HPhob | C:268(PHE) | 69 | 164 |
|  | HPhob | C:271(PHE) | 82 | 672 |
|  | HPhob | C:274(LEU) | 57 | 156 |
|  | HPhob | C:275(ALA) | 78 | 194 |
|  | HPhob | C:309(ILE) | 38 | 74 |
|  | HPhob | C:312(MET) | 71 | 299 |
|  | HPhob | C:315(GLU) | 24 | 43 |
|  | HPhob | C:316(THR) | 32 | 36 |
|  | HPhob | C:327(ILE) | 56 | 98 |
|  | HPhob | C:329(PHE) | 76 | 458 |
|  | HPhob | C:335(TYR) | 37 | 264 |
|  | HPhob | C:340(PHE) | 77 | 354 |
|  | HPhob | C:345(LEU) | 64 | 193 |
|  | HPhob | C:349(PHE) | 70 | 257 |
|  | HPhob | C:353(ILE) | 8 | 10 |
|  | HPhob | C:438(GLN) | 21 | 22 |
|  | HPhob | C:439(VAL) | 21 | 27 |
|  | HPhob | C:442(LEU) | 69 | 77 |
|  | HPhob | C:449(LEU) | 78 | 108 |
|  | HPhob | C:453(LEU) | 8 | 9 |
|  | HPhob | C:457(TRP) | 52 | 208 |
| Total Hydrophobic interactions: | | | | 3723 |

**Supplemental Table 15.** Summary of hydrogen bonds and hydrophobic interactions of 84 ligands docked into LXRβ LBD pocket (conformation of PDBID: 1UPV).

|  | **Type** | **Residue** | **# of ligands involved** | **# of contacts** |
| --- | --- | --- | --- | --- |
|  | HBond | A:272(THR) | 18 | 18 |
|  | HBond | A:278(SER) | 5 | 5 |
|  | HBond | A:281(GLU) | 17 | 17 |
|  | HBond | A:312(MET) | 2 | 2 |
|  | HBond | A:316(THR) | 14 | 14 |
|  | HBond | A:328(THR) | 2 | 2 |
|  | HBond | A:329(PHE) | 1 | 1 |
|  | HBond | A:435(HIS) | 7 | 7 |
| Total Hydrogen Bonds interactions: | | | | 66 |
|  |  |  |  |  |
|  | **Type** | **Residue** | **# of ligands involved** | **# of contacts** |
|  | HPhob | A:268(PHE) | 50 | 73 |
|  | HPhob | A:271(PHE) | 76 | 397 |
|  | HPhob | A:274(LEU) | 24 | 34 |
|  | HPhob | A:275(ALA) | 66 | 154 |
|  | HPhob | A:281(GLU) | 1 | 1 |
|  | HPhob | A:282(ILE) | 1 | 1 |
|  | HPhob | A:309(ILE) | 14 | 15 |
|  | HPhob | A:312(MET) | 83 | 222 |
|  | HPhob | A:313(LEU) | 1 | 1 |
|  | HPhob | A:315(GLU) | 49 | 85 |
|  | HPhob | A:316(THR) | 26 | 28 |
|  | HPhob | A:327(ILE) | 73 | 176 |
|  | HPhob | A:329(PHE) | 75 | 452 |
|  | HPhob | A:335(TYR) | 12 | 21 |
|  | HPhob | A:340(PHE) | 82 | 391 |
|  | HPhob | A:345(LEU) | 78 | 202 |
|  | HPhob | A:349(PHE) | 67 | 244 |
|  | HPhob | A:353(ILE) | 3 | 3 |
|  | HPhob | A:438(GLN) | 14 | 14 |
|  | HPhob | A:439(VAL) | 25 | 28 |
|  | HPhob | A:442(LEU) | 31 | 36 |
|  | HPhob | A:449(LEU) | 49 | 53 |
|  | HPhob | A:453(LEU) | 10 | 12 |
|  | HPhob | A:457(TRP) | 26 | 66 |
| Total Hydrophobic interactions: | | | | 2709 |

The resulted compounds’ docking score values for each LXR conformation are represented in Supplemental Table 6. For a reference, the docking scores of co-crystalized compounds are indicated as well. Different receptor conformations accommodate particular ligand structures better than others, thus the score values could vary significantly for the same ligand, depending on the receptor’s X-ray data used.

**Supplemental Table 16**. Glide XP docking scores of compounds docked into the LXRα and LXRβ crystal structures.

| Identification | Chemical Structure | LXRα receptor | | LXRβ receptor | | |
| --- | --- | --- | --- | --- | --- | --- |
| PDBID |  | 5AVI | 3IPQ | 5HJP | 1PQC | 1UPV |
| Co-crystalized ligands (CCL) structures |  | 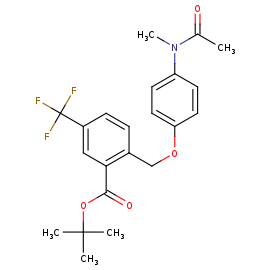 | 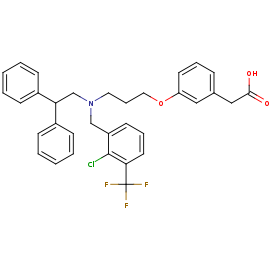 | 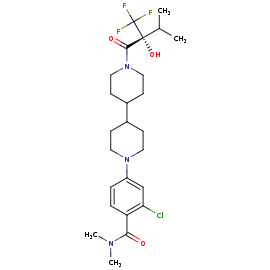 | 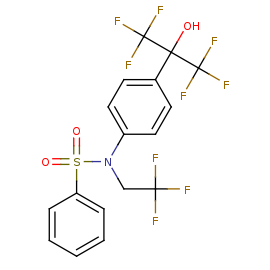 | 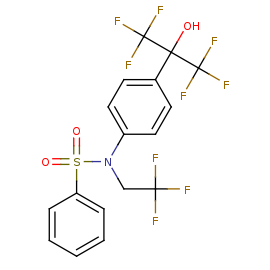 |
| CCL scores |  | -12.25 | -18.51 | -14.61 | -11.89 | -11.00 |
| 7-DHC | 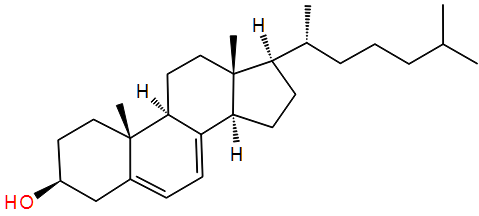 | -11.19 | -9.85 | -11.08 | -8.91 | -9.24 |
| 20*S*(OH)7DHC | 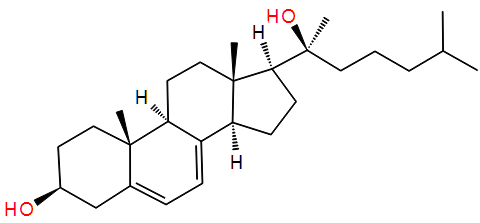 | -11.11 | -10.16 | -10.65 | -10.11 | -9.36 |
| 22*R*(OH)7DHC | 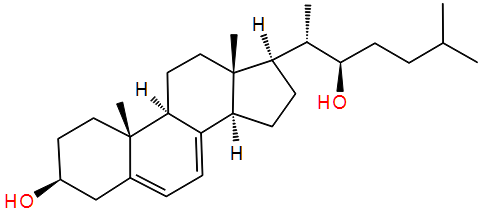 | -11.16 | -10.21 | -10.82 | -9.47 | -9.72 |
| 20*R*,22*R*(OH)_2_7DHC | 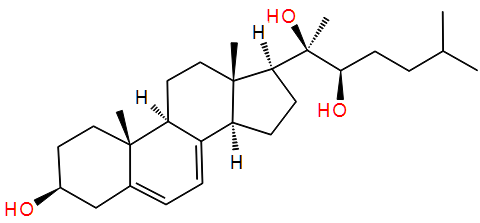 | -11.99 | -10.02 | -11.02 | -10.38 | -10.45 |
| 24*S*(OH)7DHC | 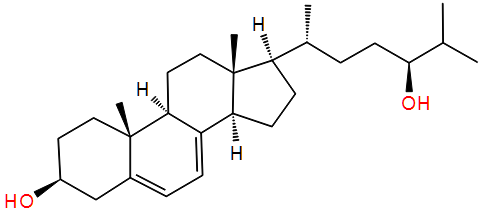 | -11.06 | -9.65 | -11.19 | -8.86 | -10.06 |
| 22*S*(OH)7DHC | 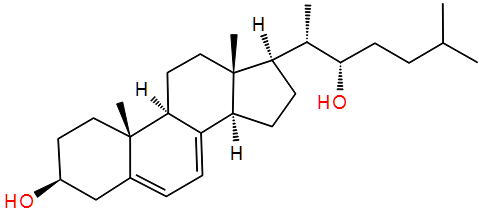 | -11.01 | -10.21 | -10.94 | -9.70 | -9.05 |
| 24*R*(OH)7DHC | 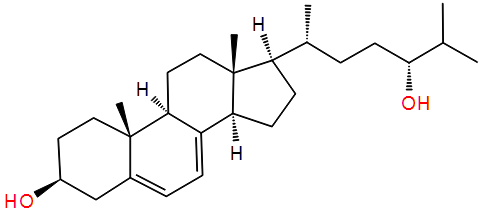 | -9.73 | -10.78 | -10.47 | -9.32 | -10.20 |
| 20*R*,22*S*(OH)_2_7DHC | 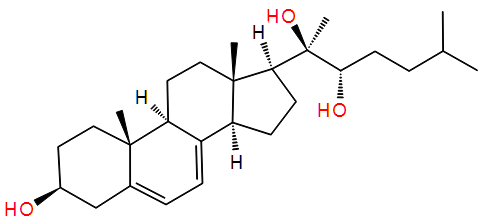 | -11.98 | -10.88 | -11.12 | -10.94 | -10.26 |
| 25(OH)7DHC | 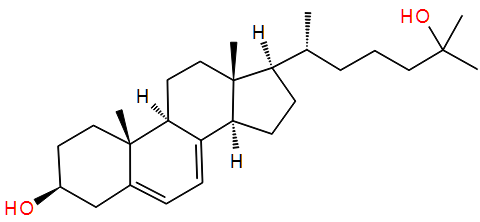 | -10.88 | -10.80 | -10.71 | -8.14 | -8.78 |
| 25*R*,27(OH)7DHC | 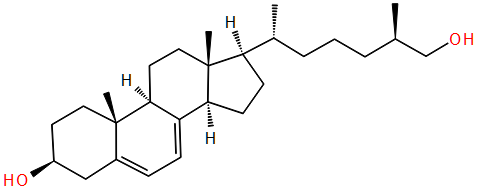 | -10.99 | -10.33 | -11.40 | -9.81 | -10.50 |
| 25*S*,27(OH)7DHC | 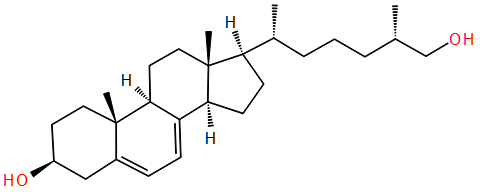 | -11.25 | -10.33 | -11.25 | -9.47 | -10.50 |
| 7DHP | 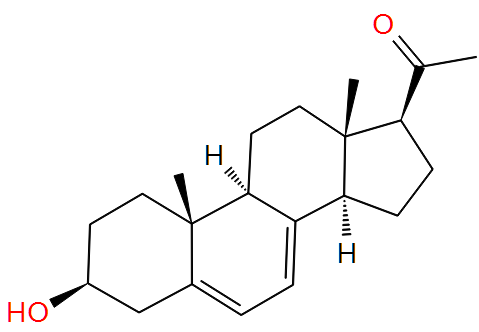 | -10.62 | -7.63 | -9.82 | -10.45 | -10.29 |
| 17(OH)7DHP | 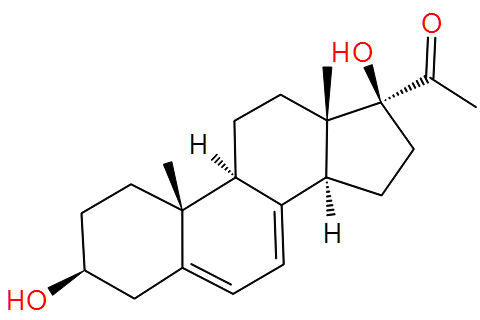 | -10.31 | -10.15 | -11.01 | -11.27 | -10.97 |
| 17*S*,20*S*(OH)_2_7DHP | 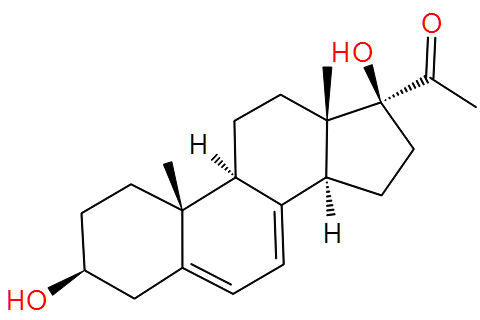 | -9.25 | -9.78 | -10.88 | -11.14 | -10.80 |
| 21(OH)7DHP | 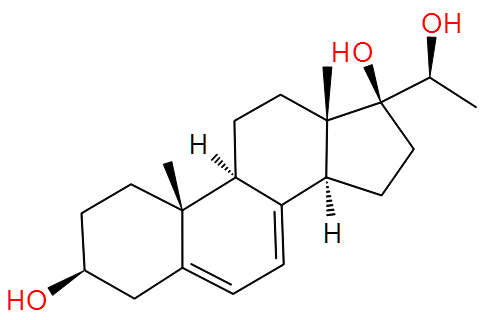 | -8.73 | -8.94 | -10.21 | -10.86 | -10.24 |
| 17*S*,20*R*(OH)_2_7DHP | 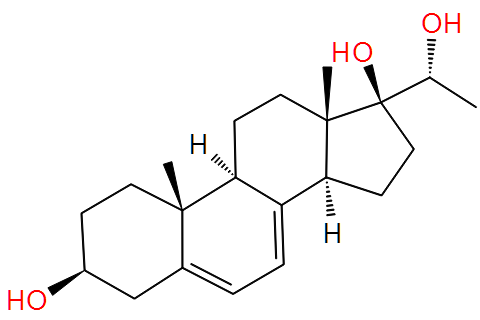 | -9.16 | -10.24 | -9.51 | -10.77 | -10.96 |
| Vitamin D3 (D3) | 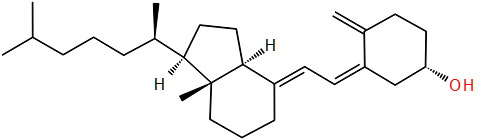 | -11.01 | -10.58 | -11.24 | -9.92 | -8.61 |
| 20*S*(OH)D3 | 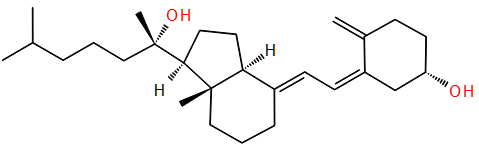 | -10.918 | -10.141 | -12.017 | -9.896 | -9.493 |
| 22*S*(OH)D3 | 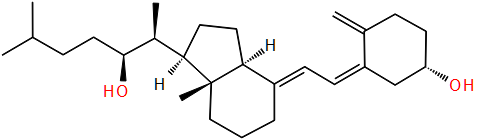 | -11.04 | -10.15 | -12.18 | -8.80 | -9.83 |
| 22*R*(OH)D3 | 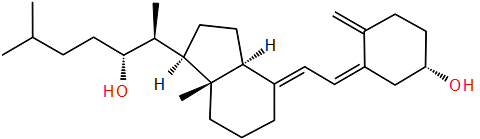 | -11.05 | -10.16 | -11.86 | -10.48 | -10.36 |
| 24*S*(OH)D3 | 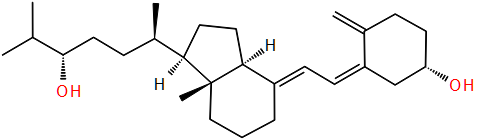 | -11.16 | -10.73 | -11.35 | -9.88 | -10.31 |
| 24*R*(OH)D3 | 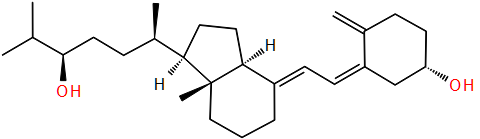 | -10.75 | -10.46 | -11.25 | -9.86 | -9.96 |
| 25(OH)D3 | 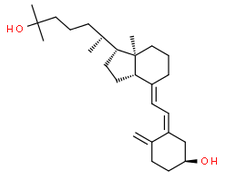 | -10.936 | -10.884 | -11.705 | -8.914 | -9.806 |
| (25*S*),27(OH)D3 | 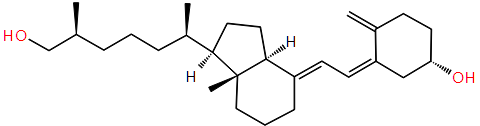 | -11.13 | -10.29 | -13.04 | -9.24 | -10.23 |
| (25*R*),27(OH)D3 | 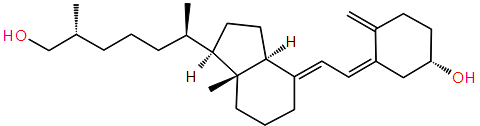 | -11.63 | -10.88 | -12.83 | -7.60 | -10.42 |
| 20*S*,22*S*(OH)_2_D3 | 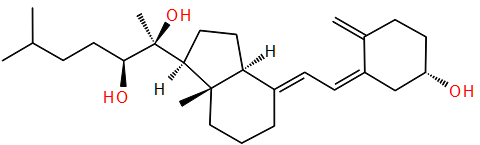 | -12.67 | -11.29 | -12.98 | -12.36 | -9.61 |
| 20*S*,22*R*(OH)_2_D3 | 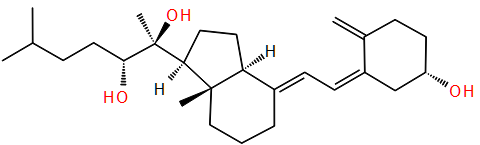 | -12.28 | -10.29 | -10.79 | -12.13 | -10.58 |
| 20*R*,23*S*(OH)_2_D3 | 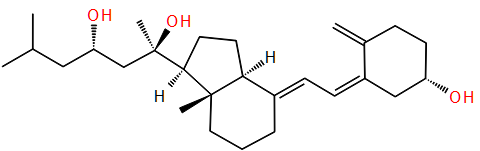 | -11.96 | -11.00 | -12.16 | -8.93 | -11.39 |
| 20*R*,23*R*(OH)*_2_*D3 | 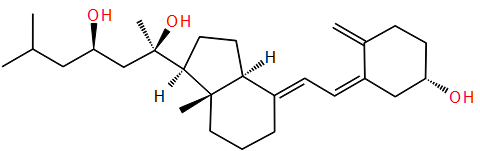 | -11.70 | -10.47 | -11.55 | -7.98 | -10.14 |
| 20*S*,24*S*(OH)_2_D3 | 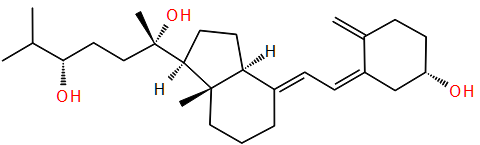 | -11.39 | -10.77 | -11.96 | -10.17 | -10.20 |
| 20*S*,24*R*(OH)_2_D3 | 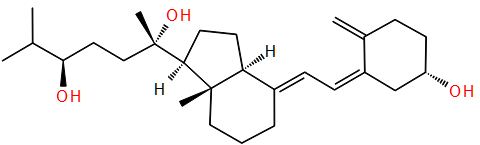 | -11.81 | -11.11 | -11.96 | -10.26 | -10.60 |
| 20*S*,25(OH)_2_D3 | 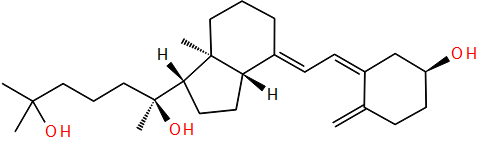 | -11.39 | -10.15 | -11.74 | -10.19 | -10.49 |
| 20*S*,25*S*,26(OH)_3_D3 | 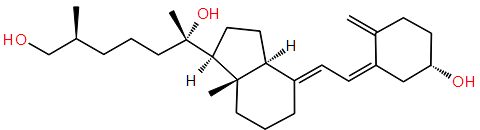 | -11.88 | -11.00 | -12.69 | -10.12 | -10.25 |
| 20*S*,25*R*,26(OH)_2_D3 | 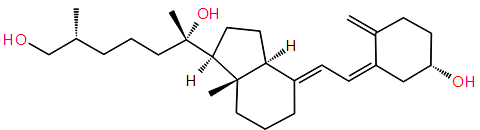 | -11.94 | -11.39 | -13.07 | -10.32 | -10.47 |
| 1α,20*S*(OH)_2_D3 | 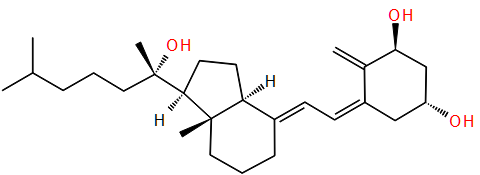 | -11.63 | -11.382 | -12.144 | -9.854 | -10.048 |
| 1α,25(OH)_2_D3 | 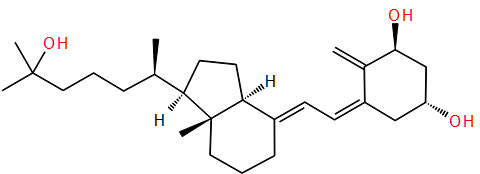 | -11.466 | -10.698 | -11.226 | -10.12 | -10.503 |
| 1α,20*S*,23*R*(OH)_3_D3 | 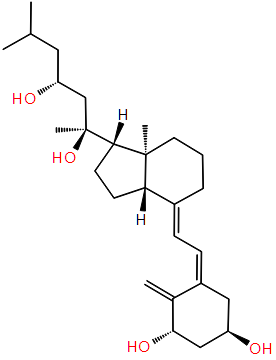 | -12.70 | -11.10 | -12.65 | -9.36 | -9.41 |
| 1α,20*S*,23*S*(OH)_3_D3 | 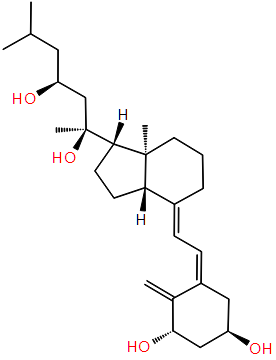 | -12.65 | -10.32 | -13.60 | -9.68 | -11.95 |
| 1α,20*S*,24*R*(OH)_3_D3 | 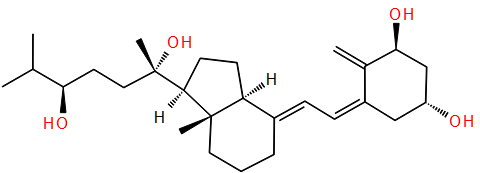 | -11.57 | -10.57 | -11.86 | -10.39 | -10.37 |
| 1α,20*S*,24*S*(OH)_3_D3 | 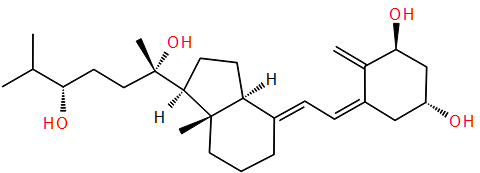 | -11.92 | -11.24 | -12.44 | -10.94 | -9.66 |
| 1α,20*S*,25(OH)_3_D3 | 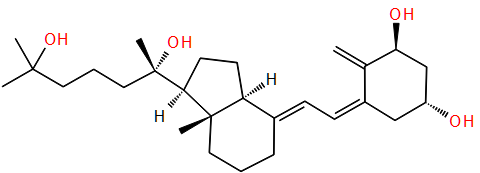 | -12.13 | -11.50 | -12.47 | -10.00 | -9.48 |
| 1α,20*S*,26*R*(OH)_3_D3 | 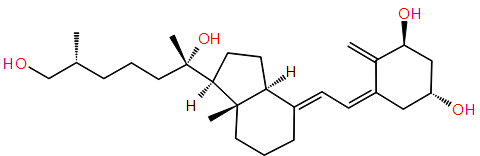 | -11.83 | -11.09 | -13.46 | -10.13 | -10.52 |
| 1α,20*S*,26*S*(OH)_3_D3 | 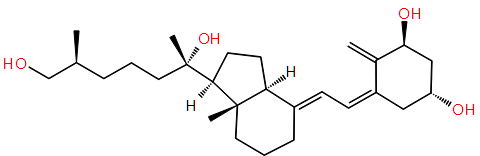 | -11.43 | -11.03 | -12.66 | -10.54 | -10.41 |
| 17,20,23(OH)_3_D3 | 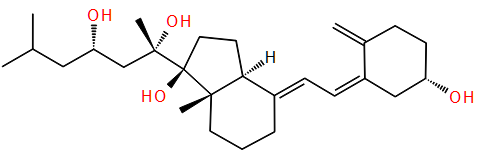 | -11.90 | -10.82 | -12.92 | -12.08 | -10.13 |
| pD | 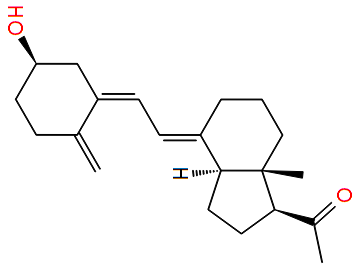 | -10.79 | -9.92 | -11.12 | -10.98 | -9.48 |
| 17*S*(OH)pD | 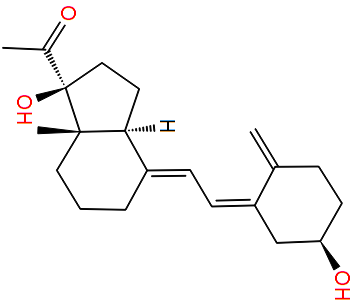 | -10.42 | -9.90 | -9.88 | -11.25 | -11.46 |
| 17*R*,20*S*(OH)_2_pD | 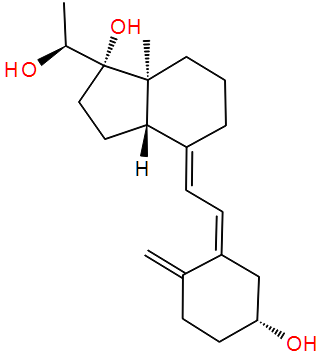 | -9.55 | -9.02 | -10.13 | -11.62 | -11.10 |
| 17*R*,20*R*(OH)_2_pD | 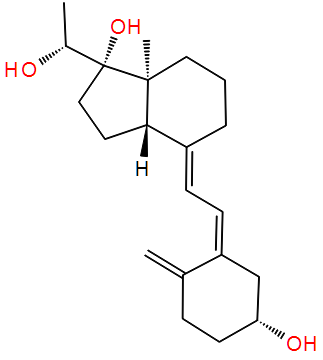 | -10.19 | -8.66 | -9.81 | -10.86 | -10.53 |
| 21(OH)pD | 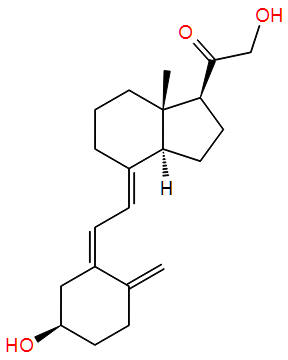 | -11.14 | -10.83 | -11.26 | -11.57 | -10.97 |
| Lumisterol (L3) | 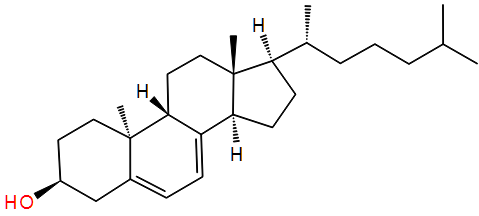 | -10.34 | -8.76 | -10.48 | -9.50 | -8.42 |
| 20*S*(OH)L3 | 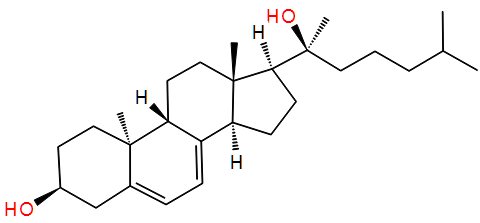 | -9.825 | -10.121 | -10.632 | -9.771 | -9.821 |
| 22*R*(OH)L3 | 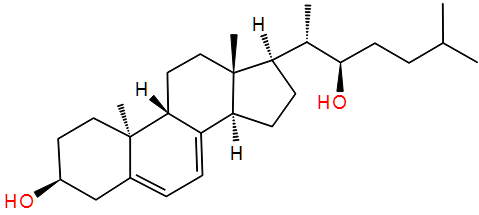 | -9.82 | -7.40 | -11.77 | -10.45 | -10.45 |
| 24*S*(OH)L3 | 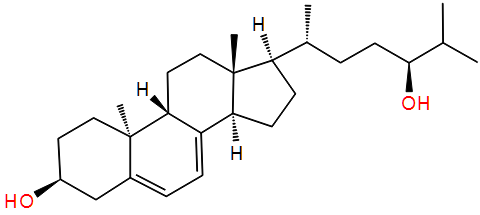 | -10.53 | -9.95 | -11.16 | -9.35 | -9.28 |
| 22*S*(OH)L3 | 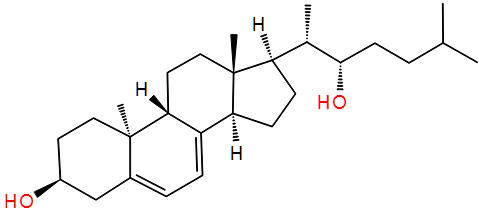 | -10.27 | -10.30 | -11.75 | -10.38 | -10.39 |
| 24*R*(OH)L3 | 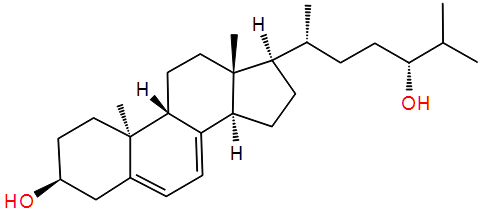 | -10.87 | -9.64 | -11.28 | -9.54 | -9.45 |
| 25(OH)L3 | 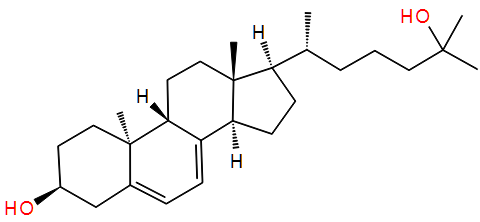 | -10.43 | -9.38 | -11.03 | -9.70 | -10.20 |
| (25*R*),27(OH)L3 | 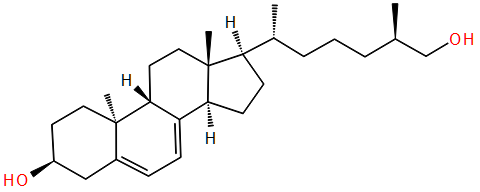 | -10.14 | -8.76 | -11.29 | -10.29 | -10.54 |
| (25*S*),27(OH)L3 | 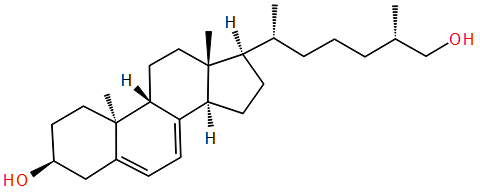 | -10.32 | -8.47 | -10.84 | -9.42 | -10.49 |
| 20*R*,22*S*(OH)_2_L3 | 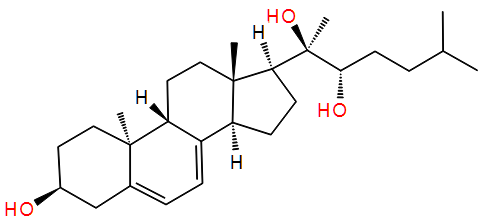 | -10.80 | -9.29 | -12.55 | -10.37 | -9.35 |
| 20*R*,22*R*(OH)_2_L3 | 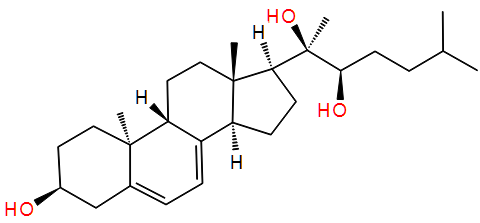 | -11.366 | -8.158 | -13.028 | -11.087 | -10.706 |
| pL | 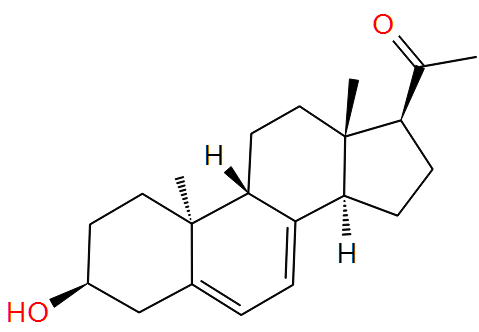 | -10.34 | -9.98 | -10.01 | -10.36 | -9.75 |
| 17*S*(OH)pL | 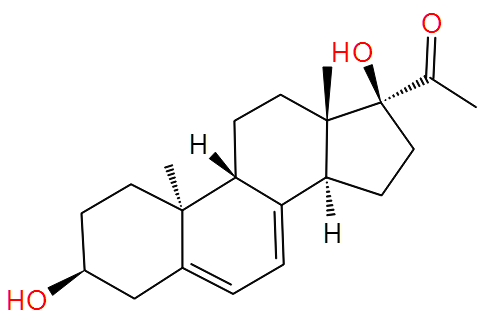 | -10.68 | -10.36 | -11.11 | -10.76 | -10.48 |
| 17*S*,20*S*(OH)_2_pL | 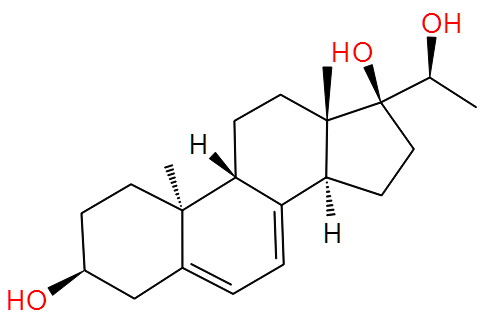 | -11.84 | -11.61 | -12.03 | -11.32 | -11.22 |
| 17*S*,20*R*(OH)_2_pL | 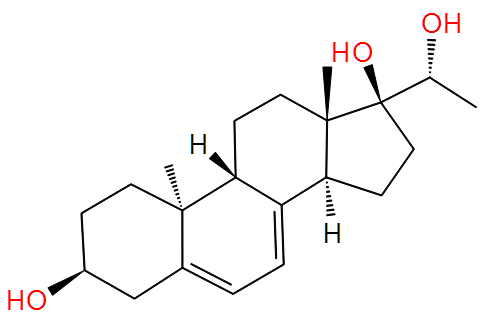 | -11.85 | -10.31 | -11.25 | -10.75 | -11.14 |
| 21(OH)pL | 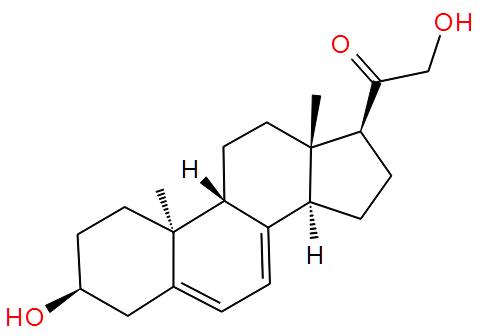 | -10.72 | -10.18 | -10.48 | -10.22 | -10.35 |
| pT | 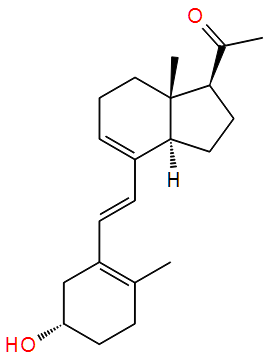 | -10.01 | -9.23 | -9.96 | -10.45 | -10.51 |
| 17S(OH)pT | 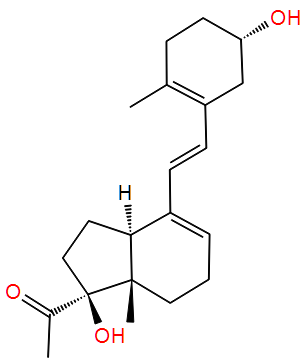 | -9.69 | -8.92 | -10.56 | -10.95 | -11.21 |
| 17S,20S(OH)_2_pT | 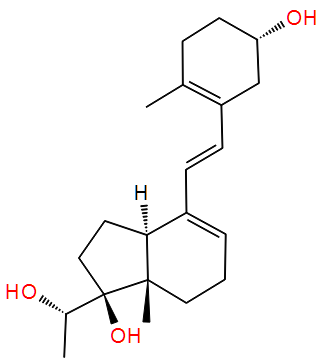 | -9.96 | -9.52 | -10.64 | -11.24 | -11.63 |
| 21(OH)pT | 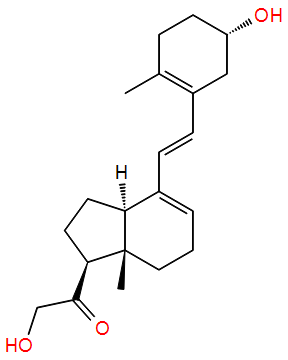 | -10.06 | -9.08 | -10.51 | -11.71 | -11.56 |
| 17*S*,20*R*(OH)_2_pT | 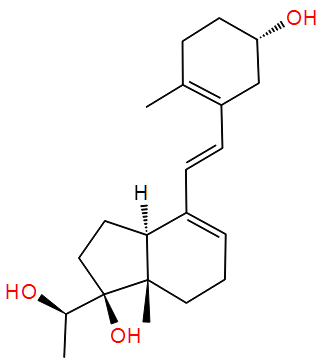 | -10.41 | -9.63 | -10.79 | -10.22 | -10.99 |
| Tachysterol (T3) | 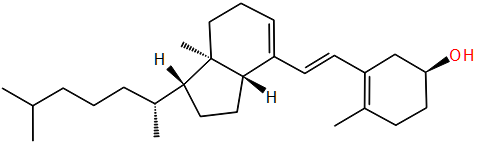 | -10.66 | -10.40 | -11.43 | -9.46 | -10.38 |
| 22S(OH)T3 | 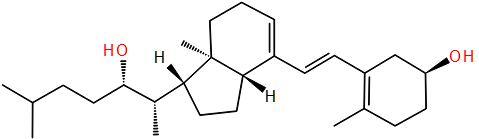 | -12.11 | -10.95 | -11.56 | -9.73 | -11.27 |
| 24*S*(OH)T3 | 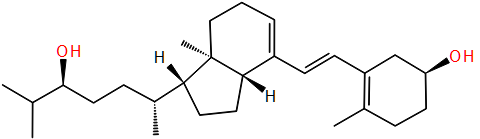 | -10.97 | -10.68 | -11.93 | -10.28 | -10.80 |
| 20*S*(OH)T3 | 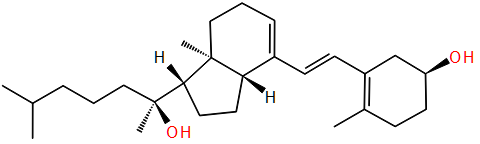 | -10.79 | -11.00 | -11.43 | -9.16 | -11.48 |
| 22*R*(OH)T3 | 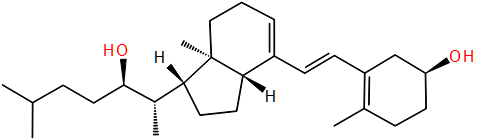 | -10.31 | -10.32 | -11.41 | -8.92 | -11.27 |
| 20*R*,22*S*(OH)_2_Tachysterol | 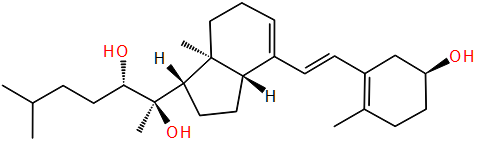 | -11.80 | -11.93 | -11.70 | -10.54 | -11.39 |
| 25(OH)T3 | 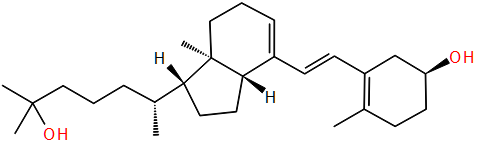 | -11.54 | -10.93 | -11.98 | -10.30 | -12.03 |
| 2*4R*(OH)T3 | 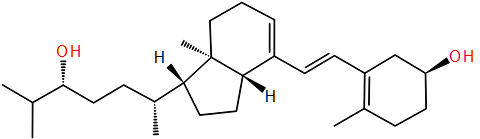 | -10.86 | -10.52 | -11.33 | -10.09 | -10.97 |
| 25*R*,27(OH)T3 | 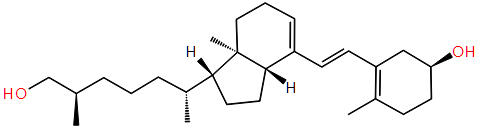 | -10.39 | -10.04 | -12.21 | -9.64 | -10.90 |
| 20*R*,22*R*(OH)_2_T3l | 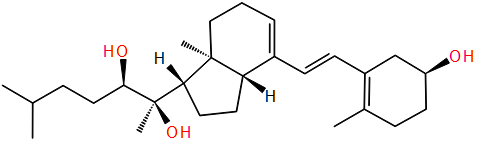 | -11.23 | -10.82 | -12.25 | -9.89 | -11.82 |
| calcitroic acid | 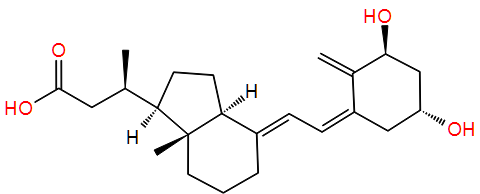 | -10.79 | -10.53 | -12.51 | -10.10 | -11.36 |
| 20*S*(OH)Cholesterol | 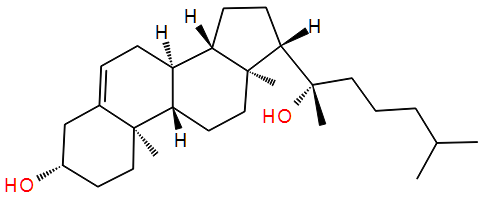 | -10.65 | -10.20 | -10.82 | -8.83 | -9.13 |
| 22*R*(OH)Cholesterol |  | -11.62 | -10.20 | -11.20 | -9.50 | -8.84 |
| 22*S*(OH)Cholesterol |  | -10.55 | -9.99 | -11.01 | -9.26 | -8.78 |

**Identification of LXRα and LXRβ receptor structure in complex with the selected D3 and L3 compounds**

**Supplemental Table 17.** LXRα and LXRβ receptor selection for four selected D3 derivatives based on the comparison of alignment parameters of the selected ligands with co-crystalized ligand and binding energy (kcal/mol).

|  | Receptor | PDB ID  (Resolution) | PC-score (Query) | RMSD_LS_ | Binding energy | Decision (PDB ID) |
| --- | --- | --- | --- | --- | --- | --- |
| 1,20(OH)_2_D3 | LXR$\alpha$ | 3IPQ (2 Å) | 0.4718 | 2.0568 | -11.382 | 5AVI |
|  |  | 5AVI (2.7 Å) | 0.4945 | 2.8627 | -11.63 |  |
|  | LXR$\beta$ | 1PQC (2.8 Å) | 0.497 | 2.2538 | -9.854 | 5HJP |
|  |  | 1UPV (2.1 Å) | 0.5047 | 1.8595 | -10.048 |  |
|  |  | 5HJP (2.6 Å) | 0.595 | 1.5428 | -12.144 |  |
| 1,25(OH)_2_D3 | LXR$\alpha$ | 3IPQ (2 Å) | 0.4526 | 3.959 | -10.698 | 5AVI |
|  |  | 5AVI (2.7 Å) | 0.5012 | 1.3544 | -11.466 |  |
|  | LXR$\beta$ | 1PQC (2.8 Å) | 0.536 | 1.3679 | -10.12 | 5HJP |
|  |  | 1UPV (2.1 Å) | 0.5009 | 1.4402 | -10.503 |  |
|  |  | 5HJP (2.6 Å) | 0.5785 | 4.0727 | -11.226 |  |
| 20(OH)D3 | LXR$\alpha$ | 3IPQ (2 Å) | 0.4912 | 4.0744 | -10.141 | 5AVI |
|  |  | 5AVI (2.7 Å) | 0.5162 | 2.7116 | -10.918 |  |
|  | LXR$\beta$ | 1PQC (2.8 Å) | 0.5194 | 1.7027 | -9.896 | 5HJP |
|  |  | 1UPV (2.1 Å) | 0.4949 | 2.0074 | -9.493 |  |
|  |  | 5HJP (2.6 Å) | 0.5954 | 2.1342 | -12.017 |  |
| 25(OH)D3 | LXR$\alpha$ | 3IPQ (2 Å) | 0.4892 | 3.8453 | -10.884 | 5AVI |
|  |  | 5AVI (2.7 Å) | 0.5006 | 1.216 | -10.936 |  |
|  | LXR$\beta$ | 1PQC (2.8 Å) | 0.5079 | 1.3323 | -8.914 | 5HJP |
|  |  | 1UPV (2.1 Å) | 0.4793 | 1.6847 | -9.806 |  |
|  |  | 5HJP (2.6 Å) | 0.5984 | 3.1124 | -11.705 |  |

**Supplemental Table 18.** LXRα and LXRβ receptor selection for two selected L3 derivatives based on the comparison of alignment parameters of the selected ligands with co-crystalized ligand and binding energy (kcal/mol).

|  | Receptor | PDB ID  (Resolution) | PC-score (Query) | RMSD_LS_ | Binding energy | Decision (PDB ID) |
| --- | --- | --- | --- | --- | --- | --- |
| 20,22(OH)_2_L3 | LXR$\alpha$ | 3IPQ (2 Å) | 0.4792 | 1.823 | -8.158 | 5AVI |
|  |  | 5AVI (2.7 Å) | 0.4873 | 1.9761 | -11.366 |  |
|  | LXR$\beta$ | 1PQC (2.8 Å) | 0.4956 | 1.7801 | -11.087 | 5HJP |
|  |  | 1UPV (2.1 Å) | 0.4672 | 1.3596 | -10.706 |  |
|  |  | 5HJP (2.6 Å) | 0.6048 | 2.6399 | -13.028 |  |
| 20(OH)L3 | LXR$\alpha$ | 3IPQ (2 Å) | 0.46 | 2.8754 | -10.121 | 5AVI |
|  |  | 5AVI (2.7 Å) | 0.5116 | 1.7182 | -9.825 |  |
|  | LXR$\beta$ | 1PQC (2.8 Å) | 0.4627 | 1.3623 | -9.771 | 5HJP |
|  |  | 1UPV (2.1 Å) | 0.4941 | 1.8642 | -9.821 |  |
|  |  | 5HJP (2.6 Å) | 0.5689 | 3.464 | -10.632 |  |

Based on PC-score (Query), RMSD_LS_ and docking score shown in Table S7 and S8, crystal structures of LXR$\alpha$ (PDBID 5AVI) and LXR$\beta$ (PDBID 5HJP) were chosen as receptors for all of the selected Vitamin D3 and Lumisterol compounds for the further molecular dynamics simulations and binding free energy analyses.

**Ligand Parameterization**

The force fields for the selected four D3 and two L3 derivatives that is compatible with general AMBER force field were determined with ANTECHAMBER program^2^. The Restrained ElectroStatic Potential fit (RESP) charges for the selected four D3 and two L3 derivatives were obtained with the combined ANTECHAMBER program and Gaussian 16 quantum mechanics Program^3^. The selected four D3 and two L3 structure generated using ACD/Structure Elucidator^4^ and RESP charge for the ligands are shown as Fig. S9 – S14 and Supplemental Table 19 – 24.

**Selected four Vitamin D3 and two Lumisterol derivatives structure and force field charge parameters**

**Figure S9.** 1,20(OH)_2_D3, including the atom naming matching force field charge parameter

**Supplemental Table 19.** 1,20(OH)_2_D3 force field charge parameter (in electron units); see Figure S9 for atom naming scheme

| Atom | C1 | C2 | C3 | C4 | C5 | C6 | C7 | C8 |
| --- | --- | --- | --- | --- | --- | --- | --- | --- |
| Charge(e) | 0.181794 | -0.222696 | 0.372272 | -0.114296 | -0.11466 | -0.164796 | -0.128178 | -0.122065 |
| Atom | C9 | C10 | C11 | C12 | C13 | C14 | C15 | C16 |
| Charge(e) | 0.04594 | 0.159138 | 0.017623 | -0.028306 | 0.193427 | -0.034431 | -0.110482 | -0.153235 |
| Atom | C17 | C18 | C19 | C20 | C21 | C22 | C23 | C24 |
| Charge(e) | 0.06124 | -0.140081 | -0.403652 | 0.234848 | -0.067705 | -0.042482 | 0.021216 | -0.209053 |
| Atom | C25 | C26 | C27 | O1 | O2 | O3 | H1 | H2 |
| Charge(e) | 0.211108 | -0.269491 | -0.269491 | -0.701907 | -0.635322 | -0.682715 | 0.048752 | 0.077939 |
| Atom | H3 | H4 | H5 | H6 | H7 | H8 | H9 | H10 |
| Charge(e) | 0.077939 | 0.004401 | 0.063872 | 0.063872 | 0.043182 | 0.160558 | 0.150615 | -0.013128 |
| Atom | H11 | H12 | H13 | H14 | H15 | H16 | H17 | H18 |
| Charge(e) | -0.013128 | 0.0005 | 0.0005 | 0.004501 | 0.004501 | 0.045308 | 0.045308 | 0.057221 |
| Atom | H19 | H20 | H21 | H22 | H23 | H24 | H25 | H26 |
| Charge(e) | 0.057221 | -0.011449 | 0.026536 | 0.026536 | 0.026536 | 0.153291 | 0.153291 | 0.411386 |
| Atom | H27 | H28 | H29 | H30 | H31 | H32 | H33 | H34 |
| Charge(e) | 0.028258 | 0.028258 | 0.028258 | 0.001354 | 0.001354 | 0.028697 | 0.028697 | 0.054235 |
| Atom | H35 | H36 | H37 | H38 | H39 | H40 | H41 | H42 |
| Charge(e) | 0.054235 | -0.002081 | 0.063488 | 0.063488 | 0.063488 | 0.063488 | 0.063488 | 0.063488 |
| Atom | H43 | H44 |  |  |  |  |  |  |
| Charge(e) | 0.405696 | 0.412484 |  |  |  |  |  |  |

**Figure S10**. 1,25(OH)_2_D3, including the atom naming matching force field charge parameter

**Supplemental Table 20**. 1,25(OH)_2_D3 force field charge parameter (in electron units); see Figure S10 for atom naming scheme

| Atom | C1 | C2 | C3 | C4 | C5 | C6 | C7 | C8 |
| --- | --- | --- | --- | --- | --- | --- | --- | --- |
| Charge(e) | 0.140802 | -0.079305 | 0.341569 | -0.096985 | -0.074531 | -0.219893 | -0.10509 | -0.07884 |
| Atom | C9 | C10 | C11 | C12 | C13 | C14 | C15 | C16 |
| Charge(e) | 0.007548 | 0.071845 | -0.000891 | -0.010324 | 0.139555 | -0.016141 | -0.130698 | -0.081074 |
| Atom | C17 | C18 | C19 | C20 | C21 | C22 | C23 | C24 |
| Charge(e) | 0.019522 | -0.097043 | -0.386259 | 0.068645 | -0.101531 | -0.061851 | -0.037169 | 0.001631 |
| Atom | C25 | C26 | C27 | O1 | O2 | O3 | H1 | H2 |
| Charge(e) | 0.349831 | -0.181641 | -0.181641 | -0.704091 | -0.744623 | -0.663489 | 0.05263 | 0.069497 |
| Atom | H3 | H4 | H5 | H6 | H7 | H8 | H9 | H10 |
| Charge(e) | 0.069497 | 0.027247 | 0.043428 | 0.043428 | 0.059592 | 0.169606 | 0.100151 | -0.011372 |
| Atom | H11 | H12 | H13 | H14 | H15 | H16 | H17 | H18 |
| Charge(e) | -0.011372 | 0.006374 | 0.006374 | 0.017597 | 0.017597 | 0.04857 | 0.04857 | 0.029519 |
| Atom | H19 | H20 | H21 | H22 | H23 | H24 | H25 | H26 |
| Charge(e) | 0.029519 | 0.005073 | -0.014394 | 0.021548 | 0.021548 | 0.021548 | 0.154584 | 0.154584 |
| Atom | H27 | H28 | H29 | H30 | H31 | H32 | H33 | H34 |
| Charge(e) | 0.408354 | 0.021124 | 0.021124 | 0.021124 | 0.005346 | 0.005346 | 0.016173 | 0.016173 |
| Atom | H35 | H36 | H37 | H38 | H39 | H40 | H41 | H42 |
| Charge(e) | 0.050257 | 0.050257 | 0.04043 | 0.04043 | 0.04043 | 0.04043 | 0.04043 | 0.04043 |
| Atom | H43 | H44 |  |  |  |  |  |  |
| Charge(e) | 0.443601 | 0.429762 |  |  |  |  |  |  |

**Figure S11**. 20(OH)D3, including the atom naming matching force field charge parameter

**Supplemental Table 21**. 20(OH)D3 force field charge parameter (in electron units); see Figure S11 for atom naming scheme

| Atom | C1 | C2 | C3 | C4 | C5 | C6 | C7 | C8 |
| --- | --- | --- | --- | --- | --- | --- | --- | --- |
| Charge(e) | -0.038193 | -0.093053 | 0.29899 | -0.110804 | -0.09049 | -0.217497 | -0.102814 | -0.081167 |
| Atom | C9 | C10 | C11 | C12 | C13 | C14 | C15 | C16 |
| Charge(e) | 0.024641 | 0.155008 | -0.004221 | -0.033785 | 0.155644 | -0.02056 | -0.192286 | -0.117204 |
| Atom | C17 | C18 | C19 | C20 | C21 | C22 | C23 | C24 |
| Charge(e) | 0.073019 | -0.149441 | -0.420798 | 0.102618 | -0.112002 | -0.00689 | 0.005183 | -0.13642 |
| Atom | C25 | C26 | C27 | O1 | O2 | H1 | H2 | H3 |
| Charge(e) | 0.274248 | -0.190207 | -0.190207 | -0.696989 | -0.641167 | 0.044045 | 0.083036 | 0.083036 |
| Atom | H4 | H5 | H6 | H7 | H8 | H9 | H10 | H11 |
| Charge(e) | 0.006965 | 0.035059 | 0.035059 | 0.027559 | 0.027559 | 0.154061 | 0.096299 | -0.000539 |
| Atom | H12 | H13 | H14 | H15 | H16 | H17 | H18 | H19 |
| Charge(e) | -0.000539 | 0.004179 | 0.004179 | 0.015428 | 0.015428 | 0.074449 | 0.074449 | 0.05793 |
| Atom | H20 | H21 | H22 | H23 | H24 | H25 | H26 | H27 |
| Charge(e) | 0.05793 | 0.015764 | 0.031934 | 0.031934 | 0.031934 | 0.154735 | 0.154735 | 0.414287 |
| Atom | H28 | H29 | H30 | H31 | H32 | H33 | H34 | H35 |
| Charge(e) | 0.044532 | 0.044532 | 0.044532 | 0.008002 | 0.008002 | 0.015384 | 0.015384 | 0.024925 |
| Atom | H36 | H37 | H38 | H39 | H40 | H41 | H42 | H43 |
| Charge(e) | 0.024925 | -0.045809 | 0.035159 | 0.035159 | 0.035159 | 0.035159 | 0.035159 | 0.035159 |
| Atom | H44 |  |  |  |  |  |  |  |
| Charge(e) | 0.430583 |  |  |  |  |  |  |  |

**Figure S12**. 25(OH)D3, including the atom naming matching force field charge parameter

**Supplemental Table 22**. 25(OH)D3 force field charge parameter (in electron units); see Figure S12 for atom naming scheme

| Atom | C1 | C2 | C3 | C4 | C5 | C6 | C7 | C8 |
| --- | --- | --- | --- | --- | --- | --- | --- | --- |
| Charge(e) | -0.097824 | -0.048048 | 0.335303 | -0.202248 | -0.017769 | -0.258861 | -0.106521 | -0.07421 |
| Atom | C9 | C10 | C11 | C12 | C13 | C14 | C15 | C16 |
| Charge(e) | -0.004733 | 0.147167 | 0.004964 | -0.023055 | 0.146377 | -0.005591 | -0.170375 | -0.099026 |
| Atom | C17 | C18 | C19 | C20 | C21 | C22 | C23 | C24 |
| Charge(e) | 0.077691 | -0.156605 | -0.421796 | 0.024638 | -0.115302 | -0.027999 | -0.038819 | -0.161754 |
| Atom | C25 | C26 | C27 | O1 | O2 | H1 | H2 | H3 |
| Charge(e) | 0.493809 | -0.206324 | -0.206324 | -0.712525 | -0.710913 | 0.048875 | 0.072942 | 0.072942 |
| Atom | H4 | H5 | H6 | H7 | H8 | H9 | H10 | H11 |
| Charge(e) | -0.003279 | 0.055049 | 0.055049 | 0.037406 | 0.037406 | 0.17362 | 0.109933 | -0.010848 |
| Atom | H12 | H13 | H14 | H15 | H16 | H17 | H18 | H19 |
| Charge(e) | -0.010848 | 0.007569 | 0.007569 | 0.019342 | 0.019342 | 0.059711 | 0.059711 | 0.028712 |
| Atom | H20 | H21 | H22 | H23 | H24 | H25 | H26 | H27 |
| Charge(e) | 0.028712 | -0.00265 | 0.00861 | 0.030079 | 0.030079 | 0.030079 | 0.156625 | 0.156625 |
| Atom | H28 | H29 | H30 | H31 | H32 | H33 | H34 | H35 |
| Charge(e) | 0.422896 | 0.035903 | 0.035903 | 0.035903 | -0.000277 | -0.000277 | 0.013193 | 0.013193 |
| Atom | H36 | H37 | H38 | H39 | H40 | H41 | H42 | H43 |
| Charge(e) | 0.050989 | 0.050989 | 0.04754 | 0.04754 | 0.04754 | 0.04754 | 0.04754 | 0.04754 |
| Atom | H44 |  |  |  |  |  |  |  |
| Charge(e) | 0.414656 |  |  |  |  |  |  |  |

**Figure S13.** 20,22(OH)_2_L3, including the atom naming matching force field charge parameter

**Supplemental Table 23**. 20,22(OH)_2_L3 force field charge parameter (in electron units); see Figure S13 for atom naming scheme

| Atom | C1 | C2 | C3 | C4 | C5 | C6 | C7 | C8 |
| --- | --- | --- | --- | --- | --- | --- | --- | --- |
| Charge(e) | -0.038823 | -0.082526 | 0.266522 | -0.274252 | -0.022616 | -0.193027 | -0.166149 | -0.065335 |
| Atom | C9 | C10 | C11 | C12 | C13 | C14 | C15 | C16 |
| Charge(e) | -0.013002 | 0.096348 | -0.127562 | -0.129624 | 0.038487 | 0.00316 | -0.159358 | -0.079732 |
| Atom | C17 | C18 | C19 | C20 | C21 | C22 | C23 | C24 |
| Charge(e) | 0.070763 | -0.118445 | -0.107116 | 0.253318 | -0.178611 | 0.108555 | 0.006961 | -0.105879 |
| Atom | C25 | C26 | C27 | O1 | O2 | O3 | H1 | H2 |
| Charge(e) | 0.265766 | -0.279335 | -0.279335 | -0.634523 | -0.651559 | -0.62474 | 0.076913 | 0.099761 |
| Atom | H3 | H4 | H5 | H6 | H7 | H8 | H9 | H10 |
| Charge(e) | 0.030683 | 0.030683 | 0.054732 | 0.09353 | 0.09353 | 0.023821 | 0.023821 | 0.14307 |
| Atom | H11 | H12 | H13 | H14 | H15 | H16 | H17 | H18 |
| Charge(e) | 0.131299 | 0.056079 | 0.056079 | 0.051258 | 0.051258 | 0.056866 | 0.056866 | 0.021858 |
| Atom | H19 | H20 | H21 | H22 | H23 | H24 | H25 | H26 |
| Charge(e) | 0.021858 | 0.031483 | 0.028227 | 0.028227 | 0.028227 | 0.375215 | 0.031572 | 0.031572 |
| Atom | H27 | H28 | H29 | H30 | H31 | H32 | H33 | H34 |
| Charge(e) | 0.031572 | 0.066024 | 0.03371 | 0.03371 | 0.013212 | 0.013212 | -0.019801 | 0.061501 |
| Atom | H35 | H36 | H37 | H38 | H39 | H40 | H41 | H42 |
| Charge(e) | 0.061501 | 0.061501 | 0.045259 | 0.045259 | 0.045259 | 0.061501 | 0.061501 | 0.061501 |
| Atom | H43 | H44 |  |  |  |  |  |  |
| Charge(e) | 0.408679 | 0.408083 |  |  |  |  |  |  |

**Figure S14**. 20(OH)L3, including the atom naming matching force field charge parameter

**Supplemental Table 24**. 20(OH)L3 force field charge parameter (in electron units); see Figure S14 for atom naming scheme

| Atom | C1 | C2 | C3 | C4 | C5 | C6 | C7 | C8 |
| --- | --- | --- | --- | --- | --- | --- | --- | --- |
| Charge(e) | -0.047411 | -0.084206 | 0.285554 | -0.292605 | -0.038146 | -0.194684 | -0.144968 | -0.056772 |
| Atom | C9 | C10 | C11 | C12 | C13 | C14 | C15 | C16 |
| Charge(e) | -0.015875 | 0.105444 | -0.153079 | -0.064159 | 0.039588 | -0.023366 | -0.080082 | -0.028204 |
| Atom | C17 | C18 | C19 | C20 | C21 | C22 | C23 | C24 |
| Charge(e) | 0.044746 | -0.100307 | -0.083322 | 0.28674 | -0.084442 | -0.047928 | -0.014824 | -0.137541 |
| Atom | C25 | C26 | C27 | O1 | O2 | H1 | H2 | H3 |
| Charge(e) | 0.223129 | -0.286664 | -0.286664 | -0.638468 | -0.669039 | 0.06435 | 0.083272 | 0.030465 |
| Atom | H4 | H5 | H6 | H7 | H8 | H9 | H10 | H11 |
| Charge(e) | 0.030465 | 0.053266 | 0.098427 | 0.098427 | 0.025286 | 0.025286 | 0.145992 | 0.117901 |
| Atom | H12 | H13 | H14 | H15 | H16 | H17 | H18 | H19 |
| Charge(e) | 0.022802 | 0.022802 | 0.061753 | 0.061753 | 0.035178 | 0.035178 | -0.003656 | -0.003656 |
| Atom | H20 | H21 | H22 | H23 | H24 | H25 | H26 | H27 |
| Charge(e) | 0.035713 | 0.023547 | 0.023547 | 0.023547 | 0.379961 | 0.026407 | 0.026407 | 0.026407 |
| Atom | H28 | H29 | H30 | H31 | H32 | H33 | H34 | H35 |
| Charge(e) | 0.019643 | 0.019643 | 0.020046 | 0.020046 | 0.034336 | 0.034336 | -0.005127 | 0.067712 |
| Atom | H36 | H37 | H38 | H39 | H40 | H41 | H42 | H43 |
| Charge(e) | 0.067712 | 0.067712 | 0.026804 | 0.026804 | 0.026804 | 0.067712 | 0.067712 | 0.067712 |
| Atom | H44 |  |  |  |  |  |  |  |
| Charge(e) | 0.387124 |  |  |  |  |  |  |  |

**Equilibration of MD simulation systems**

For the complexes of LXRα and LXRβ with the selected four D3 and two L3 derivatives, root mean square deviation (RMSD) of the backbone of LXRs in the complexes over the 300 ns MD simulations showed that all the systems reached initial equilibration after 150 ns simulations shown as Fig. S15 ad Fig. S16. The binding free energies for all the complexes as a function of cumulative time over the 300 ns MD simulation also showed that the complexes converged after the first 150 ns MD simulation (Fig. S17 and Fig. S18). The last 150 ns of MD trajectories were used for the analyses of the binding thermodynamics of LXRα and LXRβ with the select D3 and L3 derivatives, and the conformation and motion characteristics of LXRs by binding with the different ligands.

**Figure S15**. Root mean square deviation (RMSD) for systems of LXRα bound with L3 **(A)** or D3 **(B)** derivatives equilibrate over 300ns

**Figure S16.** RMSD for systems of LXRβ bound with L3 **(A)** or D3 **(B)** derivatives equilibrate over 300ns

**Figure S17.** Binding free energy between LXRα and D3 or L3 derivatives in the complexes as a function of cumulative time over the 300ns MD simulation

**Figure S18**. Binding free energy between LXRβ and D3 or L3 derivatives in the complexes as a function of cumulative time over the 300ns MD simulation

**Hydrogen bond occupancy of more than 10% between LXR and D3 or L3 derivatives**

**Supplemental Table 25.** Hydrogen bond occupancy of more than 10% between LXRα and D3 or L3 derivatives

|  | LXRα-20,22(OH)_2_L3 | LXRα-20(OH)L3 | LXRα-1,20 (OH)_2_D3 | LXRα-1,25 (OH)_2_D3 | LXRα-20 (OH)D3 | LXRα-25 (OH)D3 |
| --- | --- | --- | --- | --- | --- | --- |
| PHE257 | 12.42% | 12.11% | 22.60% | 11.71% | 15.44% | 33.66% |
| THR258 | 35.94% | 14.39% | 13.49% |  |  | 22.32% |
| LEU260 | 13.35% | 11.89% | 39.98% | 11.22% | 12.52% | 11.50% |
| ALA261 | 15.66% |  | 11.55% |  | 29.39% | 20.34% |
| SER264 | 39.11% |  | 68.63% | 53.81% | 59.72% | 12.68% |
| GLU267 |  |  |  | 10.25% | 12.12% |  |
| MET298 | 21.76% | 28.45% | 10.17% | 19.98% | 21.09% | 12.47% |
| GLU301 |  |  | 13.45% | 23.37% | 41.62% |  |
| THR302 | 83.56% | 24.59% | 47.14% | 28.99% | 21.50% | 28.71% |
| ARG305 | 20.16% | 43.41% | 15.28% | 25.69% | 45.42% | 55.94% |
| ILE313 |  | 20.10% |  |  |  | 11.20% |
| THR314 |  | 15.28% |  |  |  |  |
| PHE315 | 33.12% |  | 21.63% | 24.84% | 18.45% | 16.42% |
| LEU316 | 25.38% |  | 19.76% |  | 17.64% |  |
| PHE326 | 13.24% |  |  |  |  | 29.42% |
| LEU331 | 11.54% |  |  |  | 17.33% | 11.69% |
| PHE335 |  | 15.37% | 11.03% | 17.74% |  | 17.05% |
| HID421 | 13.38% | 47.13% | 77.55% | 42.18% |  | 15.57% |
| TRP443 | 10.71% | 10.91% | 10.94% | 37.02% |  | 27.06% |

**Supplemental Table 26**. Hydrogen bond occupancy of more than 10% between LXRβ and D3 or L3 derivatives

|  | LXRβ-20,22(OH)_2_L3 | LXRβ-20(OH)L3 | LXRβ-1,20 (OH)_2_D3 | LXRβ-1,25 (OH)_2_D3 | LXRβ-20 (OH)D3 | LXRβ-25 (OH)D3 |
| --- | --- | --- | --- | --- | --- | --- |
| ASN239 |  |  |  | 16.79% |  |  |
| SER242 |  | 19.75% |  |  | 12.04% | 33.36% |
| PHE268 | 24.18% | 16.79% | 16.25% | 18.25% |  |  |
| PHE271 |  | 23.58% | 18.90% | 15.05% | 14.76% | 20.66% |
| THR272 | 17.20% | 14.46% | 16.06% | 56.77% | 19.28% | 26.34% |
| LEU274 |  | 30.22% | 51.03% | 18.04% | 14.99% | 26.90% |
| ALA275 | 14.15% |  | 13.36% |  | 11.89% |  |
| SER278 |  | 63.34% | 85.60% | 34.05% | 79.65% | 16.74% |
| GLH281 |  | 11.84% | 70.04% | 17.72% |  | 16.47% |
| MET312 | 33.74% | 30.50% |  |  | 10.33% | 12.52% |
| GLU315 |  |  | 29.55% | 48.55% |  | 33.68% |
| THR316 | 86.39% | 30.22% | 30.89% | 26.08% | 38.43% | 22.50% |
| ARG319 |  |  |  |  | 28.50% |  |
| PHE329 | 40.21% | 47.85% | 14.28% | 57.01% | 54.02% | 49.56% |
| LEU330 |  |  |  |  |  | 18.28% |
| PHE340 | 23.07% |  |  | 22.87% | 25.55% |  |
| LEU345 |  | 18.97% |  | 15.93% | 10.34% |  |
| PHE349 | 10.81% |  | 11.57% |  |  |  |
| HID435 |  |  | 89.13% | 81.56% | 90.15% | 93.27% |
| GLN438 | 87.90% |  |  | 15.89% |  |  |
| VAL439 | 36.18% |  |  |  |  |  |
| LEU442 | 27.46% |  |  |  |  |  |
| LEU449 | 10.42% |  |  |  |  |  |
| TRP457 |  | 23.78% |  |  |  |  |

**2D interaction map of the selected four D3 and two L3 derivatives with ligand binding regions of LXRα and LXRβ separately for the representative complex structure in the equilibrated MD simulations.**

**Figure S19.** 2D interaction map of D3 or L3 derivatives with ligand binding region of LXRα for the representative complex structure in the equilibrated MD simulations

**Figure S20.** 2D interaction map of D3 or L3 derivatives with ligand binding region of LXRβ for the representative complex structure in the equilibrated MD simulations

**Distribution of percent of eigenvalues obtained from principal component analysis (PCA) analysis**

**Figure S21**. Distribution of percent of eigenvalues obtained from PCA analysis for LXRα in complex with different D3 and L3 derivatives

**Figure S22.** Distribution of percent of eigenvalues obtained from PCA analysis for LXRβ in complex with different D3 and L3 derivatives

**(A)**

**(B)**

**Figure S23.** Varied the second and the third dominant PCA motions of helix 12/AF-2 region (in brown color) and the β-sheet/helix 6 (in yellow color) in ligand-binding pocket of LXRα **(A)** and LXRβ **(B)** by binding with different D3 and L3 derivatives. LXRα and LXRβ were shown as new cartoon and molecules were shown as licorice in green. Arrows of principal dynamic motion mode 2 were shown as black. Arrows of principal dynamic motion mode 3 were shown as red. The images were made with VMD program.

**Movie caption**

Movie_LXRAlpha_125OH2D3.mpeg: Dynamics motion of 1,25(OH)_2_D3 with LXRα

Movie_LXRBeta_125OH2D3.mpeg: Dynamics motion of 1,25(OH)_2_D3 with LXRβ

**Materials and methods for molecular dynamics (MD) simulation and binding free energy analyses**

- *Ligand force field*

The AMBER ff14SB protein force field was used as parameters for the receptors. The force field for ligands were determined using ANTECHAMBER program^5^ that is an accessory module in AMBER 14 package^2^ in conjunction with the general amber force field. RESP (Restrained ElectroStatic Potential fit) charges of each ligand were determined using ANTECHAMBER program and Gaussian 16 quantum mechanics Program^3^. Ligand geometries optimization were carried out at the B3LYP/6-31G* level and electrostatic potential (ESP) of ligands were calculated at the fit using Hartree-Fock calculation with the 6-31G* basis set using Gaussian 16 Program^3^. RESP charges of each ligand were obtained by fitting to reproduce ESP using two-stage RESP fitting method in Antechamber program. ^2^ While different local environment and conformational differences could cause small charge variations for ligands, using a fixed charge parameter set for ligands was necessary for the long MD simulation.

- *Molecular dynamics simulation*

A standard MD simulation protocol was performed as those in our previous studies^6-16^ for twelve simulated systems: LXRα with four D3 and two L3 derivatives, and LXRβ with four D3 and two L3 derivatives separately. Each complex was first minimized and solvated with TIP3P water molecules and 150 mM NaCl (physiological salt concentration) in a periodic box. At least 20 Å distance between the protein complex and the periodic box boundaries was ensured to reduce potential artifacts arising from periodicity. Additional ions of Na^+^ or Cl^-^ were added into the periodic box to neutralize the charge of protein-ligand complex. The periodic box for LXRα-ligand was ∼60Å×42Å×50 Å and for LXRβ-ligand was ∼42Å×49Å×64 Å. The solvated system was first energy-minimized with the protein complex and ions restrained but water unrestrained. The solvent was further equilibrated with the protein complex and ions restrained at a constant number-pressure-temperature (NPT) at 50K and 1 atm for 10 ps. The simulated system was warmed up via constant number-volume-temperature (NVT) to 300K by steps of 50K lasting 10 ps each with SHAKE constraints and a time-step of 2 fs. Production MD simulation of 300 ns at NPT of 300K and 1 atm was performed. In the production simulations, electrostatics interaction was calculated using the particle-mesh Ewald method with interpolation of order 4. Lennard-Jones cutoffs set at 1.0 nm. SHAKE constraint were used on all hydrogen-heavy atom bonds to permit a dynamics time step of 2 fs. All the MD simulations were performed on Cheaha cluster in University of Alabama at Birmingham, using 32 conventional 2.5 GHz Intel Xeon E5 series cores in parallel with OpenMPI (v1.10.2, <https://www.open-mpi.org/software/ompi/v1.10/> ).

- *Binding free energy analysis*

In order to better understand the binding characteristics of LXRα and LXRβ with the selected four D3 and two L3 compounds, binding free energies were calculated for the twelve complexes: LXRα with four D3 and two L3 derivatives, and LXRβ with four D3 and two L3 derivatives. The binding free energy was determined with the Molecular Mechanics Poisson-Boltzmann Surface Area (MM-PBSA) method as described in previous studies^17-20^, which was implemented using the AMBER 14 MD Software Package (<https://ambermd.org/>). The MM-PBSA method combines molecular mechanics, continuum electrostatics, solvent accessible surface area calculations, and normal mode analyses for entropy to calculate the binding free energy from a series of snapshots obtained from the trajectories of the MD simulations. The binding free energy was calculated by finding the energy difference between the complex and the receptor and ligand (equation 1).  The complex for each of the frames in the production trajectory was decomposed into receptor and ligand components.  The resulting three components (complex, receptor and ligand) were then used for the binding free energy analysis. Binding free energy of the complex structure over the cumulative time was also used to verify the equilibration of the complex structure during the MD simulations.

$\Delta G=\overline{G}_{complex}-\overline{G}_{receptor}-\overline{G}_{ligand},$ (1)

Snapshots at a time interval of 150 ps from the last 150ns MD simulation trajectories after the system equilibration were used to calculate all components of binding free energy except entropy. Due to the high computational cost for entropy calculations, snapshots at a time interval of 1 ns were used for the normal mode analysis to calculate the entropy of the system. The means and standard deviations of the binding free energy over the MD simulation trajectories were calculated after the system reached initial equilibration using the bootstrap statistical method^21^.

- Conformation, secondary structure, dynamical motion and electrostatic potential analyses based on MD trajectories

Based on MD simulation trajectories, we performed a series of analyses to better understand the conformation, secondary structure and dynamical motion and electrostatic potential characteristic of LXRα and LXRβ binding with the selected D3 or L3 derivatives. The conformation, secondary structure and dynamical motion analyses were performed using the *ptraj* program of AMBER 14. Electrostatic potential analyses were performed using APBS software(v1.2.0, <http://www.poissonboltzmann.org/>)^22,23^.

- - *Hydrogen bond formation, conformational stability and secondary structure analyses*

Hydrogen bond formation were calculated between LXRs and ligand of D3 or L3 derivatives. The hydrogen bond formed between LXRs D3 or L3 derivatives could directly contribute to the changes of LXRs’ secondary structure and conformational stability. A hydrogen bond was assigned when the distance between the hydrogen and the acceptor was < 4 Å and the angle of donor-hydrogen-acceptor was less than 30 degree. OH and NH groups were treated as donors, and oxygen and nitrogen atoms were defined as acceptors. The occupancy of each hydrogen bond was calculated based on the percentage of time that the hydrogen bond existed over the equilibrated MD simulation time.

To characterize the conformational fluctuation of LXRα and LXRβ by binding with D3 or L3 derivatives, root mean square fluctuations (RMSF) of LXRα and LXRβ were calculated on a residue-by-residue basis, and averaged over the production simulation trajectories after the systems reached equilibrium.

To characterize the change of the secondary structure of LXR bound with different D3 or L3 derivatives, we evaluated the secondary structure using DSSP method in the *cpptraj* program of AMBER14^24^. For LXRα or LXRβ in complex with D3 or L3 derivatives, the occupancy of each residue in the secondary structure was determined based on the percentage of time that the residue existed in the secondary structure over the equilibrated MD simulations.

- - *Dynamic motion analyses*

Based on the equilibrated MD simulation trajectory, principal component analysis (PCA)^25^ was performed to decompose the motions of ligand binding domain (LBD) of LXRα and LXRβ by binding with D3 or L3 derivatives into a few principal motions that are described by eigenvectors and eigenvalues. Eigenvalue characterizes a principal mode of the total motion of the system. The eigenvalues can also be used to determine the contribution of each mode to the total motion of the system. The distribution of the relative contribution of the first fifty PCA modes were calculated based on corresponding eigenvalue. The first PCA mode is usually interpreted as the direction of the largest conformational fluctuation of the system during MD simulations^26^. Porcupine plots were used to visualize the collective dynamic modes calculated from PCA analysis.

- - *Electrostatic Potential*

To evaluate the surface electrostatics of LXRα and LXRβ in complex with different D3 or L3 derivatives, we generated electrostatic potential maps for each representative structure of LXRα or LXRβ in complex with D3 or L3 derivatives using APBS software^22,23^. The *dbscan* (density-based spatial clustering of applications with noise) program^27^ in Amber 14 was used for the clustering analyses of the equilibrated MD simulation trajectories. In *dbscan*, points are considered as part of a single cluster if there are at least some other points within a neighborhood radius ε. The *dbscan* algorithm also generates an average structure for the population of each cluster called a medoid. The medoid structure in the largest cluster was chosen as representative conformation for electrostatic potential analysis. We then used the PDB2PQR software package^28,29^ to obtain the representative complex structures in PQR format that included AMBER radii and charges for each atom in the complex structure, which was then used to generate surface electrostatic potentials for each complex structure of LXRα or LXRβ with different D3 or L3 derivatives using APBS software.

**Supplemental Table 27**. Sequences of primers and references to conditions used for qRTPCR^30,31^.

| **Gene name** | **Sequences** |
| --- | --- |
| *mApoE* | ACAGATCAGCTCGAGTGGCAAA  ATCTTGCGCAGGTGTGTGGAGA |
| *mAbca1* | AGTGATAATCAAAGTCAAAGGCACAC  AGCAACTTGGCACTAGTAACTCTG |
| *hABCA1* | ACAAGATGCTGAGGGCTGAT  CCCAAGACTATGCAGCAATG |
| *mAbcg1* | TTCATCGTCCTGGGCATCTT  CGGATTTTGTATCTGAGGACGAA |
| *mAbcg5* | TGC CCT TTC TGA GTC CAG AG  GTG CTC TTT CAA TGT TCT CCA G |
| *mAbcg8* | ATG AGC TGG AAG ACG GGC TG  GCC AGT GAG AGC AAG GCT GA |
| *mLp* | TGGATGAGCGACTCCTACTTCA  CGGATCCTCTCGATGACGAA |
| *mFas* | GGC ATC ATT GGG CAC TCC TT  GCT GCA AGC ACA GCC TCT CT |
| *mCyp7a1* | AGCAACTAAACAACCTGCCAGTA CTA  GTCCGGATATTCAAGGATGCA |
| *mGapdh* | GCTACACTGAGGACCAGGTTGTC  GCCAGCCCCGGCAT |
| *hGAPDH* | GCCACATCGCTCAGACAC  GCCCAATACGACCAAATCCC |
| *mCyclophilin A* | CTGCACTGCCAAGACTGAAT  CCACAATGTTCATGCCTTCT |

M: mouse, h: human

**References**

1 Färnegårdh, M. *et al.* The three-dimensional structure of the liver X receptor β reveals a flexible ligand-binding pocket that can accommodate fundamentally different ligands. *Journal of Biological Chemistry* **278**, 38821-38828 (2003).

2 Case, D. *et al.* The FF14SB force field. *Amber* **14**, 29-31 (2014).

3 Gaussian 16 Rev. B.01 (Wallingford, CT, 2016).

4 ACD/ChemSketch ( Advanced Chemistry Development, Inc., Toronto, ON, Canada, [www.acdlabs.com](file:///C:\Users\aslomins\Documents\NuclearReceptors\LXR\SciReports\Resubmission\www.acdlabs.com), version 2020.1.2).

5 Wang, J., Wang, W., Kollman, P. A. & Case, D. A. Automatic atom type and bond type perception in molecular mechanical calculations. *Journal of molecular graphics & modelling* **25**, 247-260 (2006).

6 Pan, D. & Song, Y. Role of altered sialylation of the I-like domain of beta1 integrin in the binding of fibronectin to beta1 integrin: thermodynamics and conformational analyses. *Biophysical journal* **99**, 208-217, doi:10.1016/j.bpj.2010.03.063 (2010).

7 Yan, Q., Murphy-Ullrich, J. E. & Song, Y. H. Molecular and Structural Insight into the Role of Key Residues of Thrombospondin-1 and Calreticulin in Thrombospondin-1-Calreticulin Binding. *Biochemistry* **50**, 566-573 (2011).

8 Pan, D., Yan, Q., Chen, Y., McDonald, J. M. & Song, Y. Trifluoperazine regulation of calmodulin binding to Fas: a computational study. *Proteins* **79**, 2543-2556, doi:10.1002/prot.23081 (2011).

9 Yan, Q., Murphy-Ullrich, J. E. & Song, Y. H. Structural Insight into the Role of Thrombospondin-1 Binding to Calreticulin in Calreticulin-Induced Focal Adhesion Disassembly. *Biochemistry* **49**, 3685-3694 (2010).

10 Suever, J. D., Chen, Y., McDonald, J. M. & Song, Y. Conformation and free energy analyses of the complex of calcium-bound calmodulin and the Fas death domain. *Biophysical journal* **95**, 5913-5921 (2008).

11 Liu, Y., Pan, D., Bellis, S. L. & Song, Y. Effect of altered glycosylation on the structure of the I-like domain of beta1 integrin: a molecular dynamics study. *Proteins* **73**, 989-1000, doi:10.1002/prot.22126 (2008).

12 Lee, S. J., Song, Y. & Baker, N. A. Molecular dynamics simulations of asymmetric NaCl and KCl solutions separated by phosphatidylcholine bilayers: potential drops and structural changes induced by strong Na+-lipid interactions and finite size effects. *Biophysical journal* **94**, 3565-3576, doi:10.1529/biophysj.107.116335 (2008).

13 Song, Y., Guallar, V. & Baker, N. A. Molecular dynamics simulations of salicylate effects on the micro- and mesoscopic properties of a dipalmitoylphosphatidylcholine bilayer. *Biochemistry* **44**, 13425-13438 (2005).

14 Yang, H. & Song, Y. Structural Insight for Roles of DR5 Death Domain Mutations on Oligomerization of DR5 Death Domain-FADD Complex in the Death-Inducing Signaling Complex Formation: A Computational Study. *J Mol Model* **22**, 89, doi:10.1007/s00894-016-2941-0 (2016).

15 Wang, L., Murphy-Ullrich, J. E. & Song, Y. Multiscale simulation of the interaction of calreticulin-thrombospondin-1 complex with a model membrane microdomain. *Journal of Biomolecular Structure and Dynamics* **37**, 811-822, doi:10.1080/07391102.2018.1433065 (2019).

16 Wang, L., Pan, D., Yan, Q. & Song, Y. Activation mechanisms of alphaVbeta3 integrin by binding to fibronectin: A computational study. *Protein Sci* **26**, 1124-1137, doi:10.1002/pro.3163 (2017).

17 Kollman, P. A. *et al.* Calculating structures and free energies of complex molecules: combining molecular mechanics and continuum models. *Accounts of chemical research* **33**, 889-897 (2000).

18 Wang, W. *et al.* An analysis of the interactions between the Sem-5 SH3 domain and its ligands using molecular dynamics, free energy calculations, and sequence analysis. *Journal of the American Chemical Society* **123**, 3986-3994 (2001).

19 Ganoth, A., Friedman, R., Nachliel, E. & Gutman, M. A molecular dynamics study and free energy analysis of complexes between the Mlc1p protein and two IQ motif peptides. *Biophysical journal* **91**, 2436-2450 (2006).

20 Suever, J. D., Chen, Y., McDonald, J. M. & Song, Y. Conformation and free energy analyses of the complex of calcium-bound calmodulin and the Fas death domain. *Biophysical journal* **95**, 5913-5921 (2008).

21 Efron, B. & Tibshirani, R. J. *An Introduction to the Bootstrap*. (Chapman & Hall, 1998).

22 Baker, N. A., Sept, D., Joseph, S., Holst, M. J. & McCammon, J. A. Electrostatics of nanosystems: application to microtubules and the ribosome. *Proc Natl Acad Sci U S A* **98**, 10037–10041, doi:10.1073/pnas.181342398 (2001).

23 Jurrus, E. *et al.* Improvements to the APBS biomolecular solvation software suite. *Protein Sci* **27**, 112-128, doi:10.1002/pro.3280 (2018).

24 Kabsch, W. & Sander, C. Dictionary of protein secondary structure: pattern recognition of hydrogen-bonded and geometrical features. *Biopolymers* **22**, 2577–2637, doi:10.1002/bip.360221211 (1983).

25 Hayward, S., Kitao, A. & Go, N. Harmonicity and anharmonicity in protein dynamics: a normal mode analysis and principal component analysis. *Proteins* **23**, 177-186 (1995).

26 Berendsen, H. J. & Hayward, S. Collective protein dynamics in relation to function. *Current opinion in structural biology* **10**, 165-169 (2000).

27 Ester, M., Kriegel, H.-P., Sander, J. & Xu, X. in *Proceedings of 2nd International Conference on Knowledge Discovery and Data Mining* 226–231 (1996).

28 Dolinsky, T. J. *et al.* PDB2PQR: expanding and upgrading automated preparation of biomolecular structures for molecular simulations. *Nucleic Acids Res* **35**, W522-525, doi:10.1093/nar/gkm276 (2007).

29 Dolinsky, T. J., Nielsen, J. E., McCammon, J. A. & Baker, N. A. PDB2PQR: an automated pipeline for the setup of Poisson-Boltzmann electrostatics calculations. *Nucleic Acids Res* **32**, W665-667, doi:10.1093/nar/gkh381 (2004).

30 Gafencu, A. V. *et al.* Inflammatory signaling pathways regulating ApoE gene expression in macrophages. *J Biol Chem* **282**, 21776-21785, doi:10.1074/jbc.M611422200 (2007).

31 Kaplan, R., Gan, X., Menke, J. G., Wright, S. D. & Cai, T. Q. Bacterial lipopolysaccharide induces expression of ABCA1 but not ABCG1 via an LXR-independent pathway. *J Lipid Res* **43**, 952-959 (2002).
